# Supplementary material for: Phylogenetic position of the enigmatic genus Atherospio and description of Atherospio aestuarii sp. nov. (Annelida: Spionidae) from Japan
Source: PeerJ. 2022 Aug 25;10:e13909. doi: 10.7717/peerj.13909 (PMC9420407; doi:10.7717/peerj.13909)
Supplement: Supplemental Information 1 [file peerj-10-13909-s001.pdf]

Supplementary file S1
Multiple sequence alignment of concatenated 16S, 18S, and 28S rRNA gene sequences used for molecular phylogenetic analysis.

Data partitions
16S: 1-434
18S: 435-2137
28S: 2138-2800

>Atherospio_aestuarii_P861
CGCCTTTAGATTAAAGGTATATTCTGCCCTATGTAAATGGCCGCGGTACCCTGACCGTGCTAAGGT
AGCATAATAATTTGCCTCCTAATTAGAGGCTGGAATGAAGGAACTCACGAAAGTTTAACTGTTTCA
TTTTTACTAAAAACTATCATTCAAGTGAAGAGACTTGTATTTTATAAAAAGACAAAAAGACCCTAT
TGAGCTTTATTCTACTTATTACCCTGATAAAGAATTTTGTTGGGGCGACAAGAGAAGACTAAATCT
TCTCAACCTCCTAAAAAGATCCGAAACGATCAAAAATCTAGCTACCCTAGGGATAACAGGCTAATC
TTCTTTGAGAGCCCAAATTGACAAGAAGGATTGGCACCTCGATGTTGGCTTAGGGTACCAAATAAA
TGCAGAAGTAAGTAGGTTTGTTCAACCTTTAATACCCTATATGCTTGTCTCAAAGACTAAGCCATG
CATGTCTAAGTACAAGCTCAACATGGTGAAACCGCGAATGGCTCATTAAATCAGTTATGGTTCCTT
AGATCGTACTATCCTACTTGGATAACTGTAGTAATTCTAGAGCTAATACATGCAATCAAGCTCCGA
CCTCGCGGGAGGAGCGCTTTTATTAGACCAAAACCAATGGGGGCGACTAACTTCTGGTGACTCTGG
ATAACTTTGAGCTGATCGCACGGCCGCGCCGGCGACGTATCTTTCAAATGTCTGCCCTATCAAGTT
TCGACGGTTGGTGACTTGCCAACCGTGCTGATAACGGGTAGCGGGGAATCAGGGTTCGATTCCGGA
GAGGGAGCATGAGAAACGGCTACCACATCCAAGGAAGGCAGCAGGCGCGCAAATTACCCACTCCTA
GCTCGGGGAGGTAGTGACGAAAAATAACAATACGGGACTCTTACGAGGCCCCGTAATTGGAATGAG
TACACTTTAAATCCTTTAACGAGGATCTATTGGAGGGCAAGTCTGGTGCCAGCAGCCGCGGTAATT
CCAGCTCCAATAGCGTATATTAAAGTTGTTGCAGTTAAAAAGCTCGTAGTTGGATCTCGGGTGCAG
GCTGGCGGTCCGCCTCGCGGCGGTTTACTGCCTCCTGCTCCTCTTGCCGGTTTCCCTTGGTGCTCT
TCGCTGGGGTGGCCGGGACGTTTACTTTGAAAAAATTAGAGTGTTCAAAGCAGGCCCTGAATACTG
GTGCATGGAATAATGGAATAGGACCTCGGATTTTGTTGGTTTTCGGAAGTCGAGGTAATGATTAAT
AGGGACGGCGGGGGCATTTGTATTGCGTCGTTAGAGGTGAAATTCTTGGATCGACGCAAGACAGAC
TACAGCGAAGGCGTTTGCCAAGCACGTCTTCTTTAATCAAGAGCGACAGTCAGAGGTTCGAAGACG
ATCAGATACCGTCGTAGTTCTGACCATAAACGATGCCAGCTAGGGATTCGCCGGAGTTGTCTGACT
CGGCGAGCACCTTCCGGGAAACCAAAGCTTTTGGGTTCCGGGGGAAGTATGGTTGCAAAGCTGAAA
CTTAAAGGAATTGACGGAAGGGCACCACCAGGAGTGGAGCCTGCGGCTTAATTTGACTCAACACGG
GGAAACTCACCCGGCCCGGACACCGTAAGGATTGACAGATTGAGAGCTCTTTCTTGATTCGGTGGG
TGGTGGTGCATGGCCGTTCTTAGTTGGTGGAGCGATTTGTCTGGTTAATTCCGATAACGAACGAGA

CTCTAGCCTATTAAATAGTTCGCCGATTCGTCGGCGAAACTTCTTAGAGGGACAAGTGGCGTTCAG
CCACGCGAGATTGAGCAATAACAGGTCTGTGATGCCCTTAGATGTTCGGGGCCGCACGCGCGCTAC
ACTGAAGGACTCAGCGTGTCTTCTCCTGGCCAGAAATGGTCGGGCAACCCGTTGAACCTCCTTCGT
GCTAGGGATTGGGGCTTGCAATTCTTCCCCATGAACGAGGAATTCCCAGTAAGCGCGAGTCATCAG
CTCGCGTTGATTACGTCCCTGCCCTTTGTACACACCGCCCGTCGCTACTACCGATTGGATGGTTTA
GTGAGACCCTCGGATTGGTCCCGACGGTGGGCGACCGCCGTCGTGGTGCCGAGAAGACGGTCAAAC
TTGACTATCTAGAGGAAGTAAAAGTTCACTAAGCGGAGGAAAAGAAACTAACCAGGATTCCCCTAG
TAACGGCGAGTGAAGCGGGAAGAGCCCAGCACTGAATCCCCTGCATCGACTGTAGAGGGAACTGTA
GTGTTCGGGAGAGCCTCTGGGGCGACGGCGGCGTCCGAGTCCTCCTGATCGGGGTTACCCAGAGCG
GGTGTCAGGCCTCTACGGCGGCTTCCGTCGTCCCTCTGTGCCTCCTAGGAGTCGGGTTGTTTGAGA
ATGCAGCCCAAAGCGGGTGGTAAACTCCATCTAAGGCTAAATACTGGCACGAGACCGATAGCGGAC
AAGTACCGTGAGGGAAAGTTGAAAAGAACTTTGAAGAGAGAGTTCAAGAGTACGTGAAACCGTATA
GAGGCAAACGGATGGGACCGCAGTCGATCCGCGGGATTCAGCTTCTGCCGGGCGGGCTGCGGACCT
TCACGGGGAGTGCACTTTCCGCGGGGAGAGCGCCACGACCGCTTCTCGGGCCGTCAAGCTCCGGCG
GAAGGTAGCTCGAGTGTTATAGCCGCCGGTAGTGTCGGCCGGGGGGAGCGAGGAATGCCCGCCGGA
GACCGGTCGACTGGCGAGGACTGCTCGCAGTGCTCGCCGACTGCGCCGTTCGGGCCACCCAGGGTC
TGTGGCGAAGTCGGTTTCCCATCCGACC

>Atherospio_aestuarii_P862
CGCCTTTAGATTAAAGGTATATTCTGCCCTATGTAAATGGCCGCGGTACCCTGACCGTGCTAAGGT
AGCATAATAATTTGCCTCCTAATTAGAGGCTGGAATGAAGGAACTCACGAAAGTTTAACTGTTTCA
TTTTTACTAAAAACTATCATTCAAGTGAAGAGACTTGTATTTTATAAAAAGACAAAAAGACCCTAT
TGAGCTTTATTCTACTTATTACCCTGATAAAGAATTTTGTTGGGGCGACAAGAGAAGACTAAATCT
TCTCAACCTCCTAAAAAGATCCGAGACGATCAAAAATCTAGCTACCCTAGGGATAACAGGCTAATC
TTCTTTGAGAGCCCAAATTGACAAGAAGGATTGGCACCTCGATGTTGGCTTAGGGCACCAAATAAA
TGCAGAAGTAAGTGGGTTTGTTCAACCTTTAATACCCTATATGCTTGTCTCAAAGACTAAGCCATG
CATGTCTAAGTACAAGCTCAACATGGTGAAACCGCGAATGGCTCATTAAATCAGTTATGGTTCCTT
AGATCGTACTATCCTACTTGGATAACTGTAGTAATTCTAGAGCTAATACATGCAATCAAGCTCCGA
CCTCGCGGGAGGAGCGCTTTTATTAGACCAAAACCAATGGGGGCGACTAACTTCTGGTGACTCTGG
ATAACTTTGAGCTGATCGCACGGCCGCGCCGGCGACGTATCTTTCAAATGTCTGCCCTATCAAGTT
TCGACGGTTGGTGACTTGCCAACCGTGCTGATAACGGGTAGCGGGGAATCAGGGTTCGATTCCGGA
GAGGGAGCATGAGAAACGGCTACCACATCCAAGGAAGGCAGCAGGCGCGCAAATTACCCACTCCTA
GCTCGGGGAGGTAGTGACGAAAAATAACAATACGGGACTCTTACGAGGCCCCGTAATTGGAATGAG
TACACTTTAAATCCTTTAACGAGGATCTATTGGAGGGCAAGTCTGGTGCCAGCAGCCGCGGTAATT
CCAGCTCCAATAGCGTATATTAAAGTTGTTGCAGTTAAAAAGCTCGTAGTTGGATCTCGGGTGCAG
GCTGGCGGTCCGCCTCGCGGCGGTTTACTGCCTCCTGCTCCTCTTGCCGGTTTCCCTTGGTGCTCT

TCGCTGGGGTGGCCGGGACGTTTACTTTGAAAAAATTAGAGTGTTCAAAGCAGGCCCTGAATACTG
GTGCATGGAATAATGGAATAGGACCTCGGATTTTGTTGGTTTTCGGAAGTCGAGGTAATGATTAAT
AGGGACGGCGGGGGCATTTGTATTGCGTCGTTAGAGGTGAAATTCTTGGATCGACGCAAGACAGAC
TACAGCGAAGGCGTTTGCCAAGCACGTCTTCTTTAATCAAGAGCGACAGTCAGAGGTTCGAAGACG
ATCAGATACCGTCGTAGTTCTGACCATAAACGATGCCAGCTAGGGATTCGCCGGAGTTGTCTGACT
CGGCGAGCACCTTCCGGGAAACCAAAGCTTTTGGGTTCCGGGGGAAGTATGGTTGCAAAGCTGAAA
CTTAAAGGAATTGACGGAAGGGCACCACCAGGAGTGGAGCCTGCGGCTTAATTTGACTCAACACGG
GGAAACTCACCCGGCCCGGACACCGTAAGGATTGACAGATTGAGAGCTCTTTCTTGATTCGGTGGG
TGGTGGTGCATGGCCGTTCTTAGTTGGTGGAGCGATTTGTCTGGTTAATTCCGATAACGAACGAGA
CTCTAGCCTATTAAATAGTTCGCCGATTCGTCGGCGAAACTTCTTAGAGGGACAAGTGGCGTTCAG
CCACGCGAGATTGAGCAATAACAGGTCTGTGATGCCCTTAGATGTTCGGGGCCGCACGCGCGCTAC
ACTGAAGGACTCAGCGTGTCTTCTCCTGGCCAGAAATGGTCGGGCAACCCGTTGAACCTCCTTCGT
GCTAGGGATTGGGGCTTGCAATTCTTCCCCATGAACGAGGAATTCCCAGTAAGCGCGAGTCATCAG
CTCGCGTTGATTACGTCCCTGCCCTTTGTACACACCGCCCGTCGCTACTACCGATTGGATGGTTTA
GTGAGACCCTCGGATTGGTCCCGACGGTGGGCGACCGCCGTCGTGGTGCCGAGAAGACGGTCAAAC
TTGACTATCTAGAGGAAGTAAAAGTTCACTAAGCGGAGGAAAAGAAACTAACCAGGATTCCCCTAG
TAACGGCGAGTGAAGCGGGAAGAGCCCAGCACTGAATCCCCTGCATCGACTGTAGAGGGAACTGTA
GTGTTCGGGAGAGCCTCTGGGGCGACGGCGGCGTCCGAGTCCTCCTGATCGGGGTTACCCAGAGCG
GGTGTCAGGCCTCTACGGCGGCTTCCGTCGTCCCTCTGTGCCTCCTAGGAGTCGGGTTGTTTGAGA
ATGCAGCCCAAAGCGGGTGGTAAACTCCATCTAAGGCTAAATACTGGCACGAGACCGATAGCGGAC
AAGTACCGTGAGGGAAAGTTGAAAAGAACTTTGAAGAGAGAGTTCAAGAGTACGTGAAACCGTATA
GAGGCAAACGGATGGGACCGCAGTCGATCCGCGGGATTCAGCTTCTGCCGGGCGGGCTGCGGACCT
TCACGGGGAGTGCACTTTCCGCGGGGAGAGCGCCACGACCGCTTCTCGGGCCGTCAAGCTCCGGCG
GAAGGTAGCTCGAGTGTTATAGCCGCCGGTAGTGTCGGCCGGGGGGAGCGAGGAATGCCCGCCGGA
GACCGGTCGACTGGCGAGGACTGCTCGCAGTGCTCGCCGACTGCGCCGTTCGGGCCACCCAGGGTC
TGTGGCGAAGTCGGTTTCCCATCCGACC

>Atherospio_aestuarii_P863
CGCCTTTAGATTAAAGGTATATTCTGCCCTATGTAAATGGCCGCGGTACCCTGACCGTGCTAAGGT
AGCATAATAATTTGCCTCCTAATTAGAGGCTGGAATGAAGGAACTCACGAAAGTTTAACTGTTTCA
TTTTTACTAAAAACTATCATTCAAGTGAAGAGACTTGTATTTTATAAAAAGACAAAAAGACCCTAT
TGAGCTTTATTCTACTTATTACCCTGATAAAGAATTTTGTTGGGGCGACAAGAGAAGACTAAATCT
TCTCAACCTCCTAAAAAGATCCGAAACGATCAAAAATCTAGCTACCCTAGGGATAACAGGCTAATC
TTCTTTGAGAGCCCAAATTGACAAGAAGGATTGGCACCTCGATGTTGGCTTAGGGTACCAAATAAA
TGCAGAAGTAAGTAGGTTTGTTCAACCTTTAATACCCTATATGCTTGTCTCAAAGACTAAGCCATG
CATGTCTAAGTACAAGCTCAACATGGTGAAACCGCGAATGGCTCATTAAATCAGTTATGGTTCCTT

AGATCGTACTATCCTACTTGGATAACTGTAGTAATTCTAGAGCTAATACATGCAATCAAGCTCCGA
CCTCGCGGGAGGAGCGCTTTTATTAGACCAAAACCAATGGGGGCGACTAACTTCTGGTGACTCTGG
ATAACTTTGAGCTGATCGCACGGCCGCGCCGGCGACGTATCTTTCAAATGTCTGCCCTATCAAGTT
TCGACGGTTGGTGACTTGCCAACCGTGCTGATAACGGGTAGCGGGGAATCAGGGTTCGATTCCGGA
GAGGGAGCATGAGAAACGGCTACCACATCCAAGGAAGGCAGCAGGCGCGCAAATTACCCACTCCTA
GCTCGGGGAGGTAGTGACGAAAAATAACAATACGGGACTCTTACGAGGCCCCGTAATTGGAATGAG
TACACTTTAAATCCTTTAACGAGGATCTATTGGAGGGCAAGTCTGGTGCCAGCAGCCGCGGTAATT
CCAGCTCCAATAGCGTATATTAAAGTTGTTGCAGTTAAAAAGCTCGTAGTTGGATCTCGGGTGCAG
GCTGGCGGTCCGCCTCGCGGCGGTTTACTGCCTCCTGCTCCTCTTGCCGGTTTCCCTTGGTGCTCT
TCGCTGGGGTGGCCGGGACGTTTACTTTGAAAAAATTAGAGTGTTCAAAGCAGGCCCTGAATACTG
GTGCATGGAATAATGGAATAGGACCTCGGATTTTGTTGGTTTTCGGAAGTCGAGGTAATGATTAAT
AGGGACGGCGGGGGCATTTGTATTGCGTCGTTAGAGGTGAAATTCTTGGATCGACGCAAGACAGAC
TACAGCGAAGGCGTTTGCCAAGCACGTCTTCTTTAATCAAGAGCGACAGTCAGAGGTTCGAAGACG
ATCAGATACCGTCGTAGTTCTGACCATAAACGATGCCAGCTAGGGATTCGCCGGAGTTGTCTGACT
CGGCGAGCACCTTCCGGGAAACCAAAGCTTTTGGGTTCCGGGGGAAGTATGGTTGCAAAGCTGAAA
CTTAAAGGAATTGACGGAAGGGCACCACCAGGAGTGGAGCCTGCGGCTTAATTTGACTCAACACGG
GGAAACTCACCCGGCCCGGACACCGTAAGGATTGACAGATTGAGAGCTCTTTCTTGATTCGGTGGG
TGGTGGTGCATGGCCGTTCTTAGTTGGTGGAGCGATTTGTCTGGTTAATTCCGATAACGAACGAGA
CTCTAGCCTATTAAATAGTTCGCCGATTCGTCGGCGAAACTTCTTAGAGGGACAAGTGGCGTTCAG
CCACGCGAGATTGAGCAATAACAGGTCTGTGATGCCCTTAGATGTTCGGGGCCGCACGCGCGCTAC
ACTGAAGGACTCAGCGTGTCTTCTCCTGGCCAGAAATGGTCGGGCAACCCGTTGAACCTCCTTCGT
GCTAGGGATTGGGGCTTGCAATTCTTCCCCATGAACGAGGAATTCCCAGTAAGCGCGAGTCATCAG
CTCGCGTTGATTACGTCCCTGCCCTTTGTACACACCGCCCGTCGCTACTACCGATTGGATGGTTTA
GTGAGACCCTCGGATTGGTCCCGACGGTGGGCGACCGCCGTCGTGGTGCCGAGAAGACGGTCAAAC
TTGACTATCTAGAGGAAGTAAAAGT-CACTAAGCGGAGGAAAAGAAACTAACCAGGATTCCCCTAG
TAACGGCGAGTGAAGCGGGAARAGCCCAGCACTGAATCCCCTGCATCGACTGTAGAGGGAACTGTA
GTGTTCGGGAGAGCCTCTGGGGCGACGGCGGCGTCCGAGTCCTCCTGATCGGGGTTACCCAGAGCG
GGTGTCAGGCCTCTACGGCGGCTTCCGTCGTCCCTCTGTGCCTCCTAGGAGTCGGGTTGTTTGAGA
ATGCAGCCCAAAGCGGGTGGTAAACTCCATCTAAGGCTAAATACTGGCACGAGACCGATAGCGGAC
AAGTACCGTGAGGGAAAGTTGAAAAGAACTTTGAAGAGAGAGTTCAAGAGTACGTGAAACCGTATA
GAGGCAAACGGATGGGACCGCAGTCGATCCGCGGGATTCAGCTTCTGCCGGGCGGGCTGCGGACCT
TCACGGGGAGTGCACTTTCCGCGGGGAGAGCGCCACGACCGCTTCTCGGGCCGTCAAGCTCCGGCG
GAAGGTAGCTCGAGTGTTATAGCCGCCGGTAGTGTCGGCCGGGGGGAGCGAGGAATGCCCGCCGGA
GACCGGTCGACTGGCGAGGACTGCTCGCAGTGCTCGCCGACTGCGCCGTTCGGGCCACCCAGGGTC
TGTGGCGAAGTCGGTTTCCCATCCGAC-

>Atherospio_aestuarii_P864

CGCCTTTAGATTAAAGGTATATTCTGCCCTATGTAAATGGCCGCGGTACCCTGACCGTGCTAAGGT
AGCATAATAATTTGCCTCCTAATTAGAGGCTGGAATGAAGGAACTCACGAAAGTTTAACTGTTTCA
TTTTTACTAAAAACTATCATTCAAGTGAAGAGACTTGTATTTTATAAAAAGACAAAAAGACCCTAT
TGAGCTTTATTCTACTTATTACCCTGATAAAGAATTTTGTTGGGGCGACAAGAGAAGACTAAATCT
TCTCAACCTCCTAAAAAGATCCGAGACGATCAAAAATCTAGCTACCCTAGGGATAACAGGCTAATC
TTCTTTGAGAGCCCAAATTGACAAGAAGGATTGGCACCTCGATGTTGGCTTAGGGCACCAAATAAA
TGCAGAAGTAAGTGGGTTTGTTCAACCTTTAATACCCTATATGCTTGTCTCAAAGACTAAGCCATG
CATGTCTAAGTACAAGCTCAACATGGTGAAACCGCGAATGGCTCATTAAATCAGTTATGGTTCCTT
AGATCGTACTATCCTACTTGGATAACTGTAGTAATTCTAGAGCTAATACATGCAATCAAGCTCCGA
CCTCGCGGGAGGAGCGCTTTTATTAGACCAAAACCAATGGGGGCGACTAACTTCTGGTGACTCTGG
ATAACTTTGAGCTGATCGCACGGCCGCGCCGGCGACGTATCTTTCAAATGTCTGCCCTATCAAGTT
TCGACGGTTGGTGACTTGCCAACCGTGCTGATAACGGGTAGCGGGGAATCAGGGTTCGATTCCGGA
GAGGGAGCATGAGAAACGGCTACCACATCCAAGGAAGGCAGCAGGCGCGCAAATTACCCACTCCTA
GCTCGGGGAGGTAGTGACGAAAAATAACAATACGGGACTCTTACGAGGCCCCGTAATTGGAATGAG
TACACTTTAAATCCTTTAACGAGGATCTATTGGAGGGCAAGTCTGGTGCCAGCAGCCGCGGTAATT
CCAGCTCCAATAGCGTATATTAAAGTTGTTGCAGTTAAAAAGCTCGTAGTTGGATCTCGGGTGCAG
GCTGGCGGTCCGCCTCGCGGCGGTTTACTGCCTCCTGCTCCTCTTGCCGGTTTCCCTTGGTGCTCT
TCGCTGGGGTGGCCGGGACGTTTACTTTGAAAAAATTAGAGTGTTCAAAGCAGGCCCTGAATACTG
GTGCATGGAATAATGGAATAGGACCTCGGATTTTGTTGGTTTTCGGAAGTCGAGGTAATGATTAAT
AGGGACGGCGGGGGCATTTGTATTGCGTCGTTAGAGGTGAAATTCTTGGATCGACGCAAGACAGAC
TACAGCGAAGGCGTTTGCCAAGCACGTCTTCTTTAATCAAGAGCGACAGTCAGAGGTTCGAAGACG
ATCAGATACCGTCGTAGTTCTGACCATAAACGATGCCAGCTAGGGATTCGCCGGAGTTGTCTGACT
CGGCGAGCACCTTCCGGGAAACCAAAGCTTTTGGGTTCCGGGGGAAGTATGGTTGCAAAGCTGAAA
CTTAAAGGAATTGACGGAAGGGCACCACCAGGAGTGGAGCCTGCGGCTTAATTTGACTCAACACGG
GGAAACTCACCCGGCCCGGACACCGTAAGGATTGACAGATTGAGAGCTCTTTCTTGATTCGGTGGG
TGGTGGTGCATGGCCGTTCTTAGTTGGTGGAGCGATTTGTCTGGTTAATTCCGATAACGAACGAGA
CTCTAGCCTATTAAATAGTTCGCCGATTCGTCGGCGAAACTTCTTAGAGGGACAAGTGGCGTTCAG
CCACGCGAGATTGAGCAATAACAGGTCTGTGATGCCCTTAGATGTTCGGGGCCGCACGCGCGCTAC
ACTGAAGGACTCAGCGTGTCTTCTCCTGGCCAGAAATGGTCGGGCAACCCGTTGAACCTCCTTCGT
GCTAGGGATTGGGGCTTGCAATTCTTCCCCATGAACGAGGAATTCCCAGTAAGCGCGAGTCATCAG
CTCGCGTTGATTACGTCCCTGCCCTTTGTACACACCGCCCGTCGCTACTACCGATTGGATGGTTTA
GTGAGACCCTCGGATTGGTCCCGACGGTGGGCGACCGCCGTCGTGGTGCCGAGAAGACGGTCAAAC
TTGACTATCTAGAGGAAGTAAAAGTTCACTAAGCGGAGGAAAAGAAACTAACCAGGATTCCCCTAG
TAACGGCGAGTGAAGCGGGAAAAGCCCAGCACTGAATCCCCTGCATCGACTGTAGAGGGAACTGTA
GTGTTCGGGAGAGCCTCTGGGGCGACGGCGGCGTCCGAGTCCTCCTGATCGGGGTTACCCAGAGCG

GGTGTCAGGCCTCTACGGCGGCTTCCGTCGTCCCTCTGTGCCTCCTAGGAGTCGGGTTGTTTGAGA
ATGCAGCCCAAAGCGGGTGGTAAACTCCATCTAAGGCTAAATACTGGCACGAGACCGATAGCGGAC
AAGTACCGTGAGGGAAAGTTGAAAAGAACTTTGAAGAGAGAGTTCAAGAGTACGTGAAACCGTATA
GAGGCAAACGGATGGGACCGCAGTCGATCCGCGGGATTCAGCTTCTGCCGGGCGGGCTGCGGACCT
TCACGGGGAGTGCACTTTCCGCGGGGAGAGCGCCACGACCGCTTCTCGGGCCGTCAAGCKCCGGCG
GAAGGTAGCTCGAGTGTTATAGCCGCCGGTAGTGTCGGCCGGGGGGAGCGAGGAATGCCCGCCGGA
GACCGGTCGACTGGCGAGGACTGCTCGCAGTGCTCGCCGACTGCGCCGTTCGGGCCACCCAGGGTC
TGTGGCGAAGTCGGTTTCCCATCCGAC-

>Atherospio_aestuarii_H858
CGCCTTTAGATTAAAGGTATATTCTGCCCTATGTAAATGGCCGCGGTACCCTGACCGTGCTAAGGT
AGCATAATAATTTGCCTCCTAATTAGAGGCTGGAATGAAGGAACTCACGAAAGTTTAACTGTTTCA
TTTTTACTAAAAACTATCATTCAAGTGAAGAGACTTGTATTTTATAAAAAGACAAAAAGACCCTAT
TGAGCTTTATTCTACTTATTACCCTGATAAAGAATTTTGTTGGGGCGACAAGAGAAGACTAAATCT
TCTCAACCTCCTAAAAAGATCCGAAGCGATCAAAAATCTAGCTACCCTAGGGATAACAGGCTAATC
TTCTTTGAGAGCCCAAATTGACAAGAAGGATTGGCACCTCGATGTTGGCTTAGGGTACCAAATAAA
TGCAGAAGTAAGTAGGTTTGTTCAACCTTTAATACCCTATATGCTTGTCTCAAAGACTAAGCCATG
CATGTCTAAGTACAAGCTCAACATGGTGAAACCGCGAATGGCTCATTAAATCAGTTATGGTTCCTT
AGATCGTACTATCCTACTTGGATAACTGTAGTAATTCTAGAGCTAATACATGCAATCAAGCTCCGA
CCTCGCGGGAGGAGCGCTTTTATTAGACCAAAACCAATGGGGGCGACTAACTTCTGGTGACTCTGG
ATAACTTTGAGCTGATCGCACGGCCGCGCCGGCGACGTATCTTTCAAATGTCTGCCCTATCAAGTT
TCGACGGTTGGTGACTTGCCAACCGTGCTGATAACGGGTAGCGGGGAATCAGGGTTCGATTCCGGA
GAGGGAGCATGAGAAACGGCTACCACATCCAAGGAAGGCAGCAGGCGCGCAAATTACCCACTCCTA
GCTCGGGGAGGTAGTGACGAAAAATAACAATACGGGACTCTTACGAGGCCCCGTAATTGGAATGAG
TACACTTTAAATCCTTTAACGAGGATCTATTGGAGGGCAAGTCTGGTGCCAGCAGCCGCGGTAATT
CCAGCTCCAATAGCGTATATTAAAGTTGTTGCAGTTAAAAAGCTCGTAGTTGGATCTCGGGTGCAG
GCTGGCGGTCCGCCTCGCGGCGGTTTACTGCCTCCTGCTCCTCTTGCCGGTTTCCCTTGGTGCTCT
TCGCTGGGGTGGCCGGGACGTTTACTTTGAAAAAATTAGAGTGTTCAAAGCAGGCCCTGAATACTG
GTGCATGGAATAATGGAATAGGACCTCGGATTTTGTTGGTTTTCGGAAGTCGAGGTAATGATTAAT
AGGGACGGCGGGGGCATTTGTATTGCGTCGTTAGAGGTGAAATTCTTGGATCGACGCAAGACAGAC
TACAGCGAAGGCGTTTGCCAAGCACGTCTTCTTTAATCAAGAGCGACAGTCAGAGGTTCGAAGACG
ATCAGATACCGTCGTAGTTCTGACCATAAACGATGCCAGCTAGGGATTCGCCGGAGTTGTCTGACT
CGGCGAGCACCTTCCGGGAAACCAAAGCTTTTGGGTTCCGGGGGAAGTATGGTTGCAAAGCTGAAA
CTTAAAGGAATTGACGGAAGGGCACCACCAGGAGTGGAGCCTGCGGCTTAATTTGACTCAACACGG
GGAAACTCACCCGGCCCGGACACCGTAAGGATTGACAGATTGAGAGCTCTTTCTTGATTCGGTGGG
TGGTGGTGCATGGCCGTTCTTAGTTGGTGGAGCGATTTGTCTGGTTAATTCCGATAACGAACGAGA

CTCTAGCCTATTAAATAGTTCGCCGATTCGTCGGCGAAACTTCTTAGAGGGACAAGTGGCGTTCAG
CCACGCGAGATTGAGCAATAACAGGTCTGTGATGCCCTTAGATGTTCGGGGCCGCACGCGCGCTAC
ACTGAAGGACTCAGCGTGTCTTCTCCTGGCCAGAAATGGTCGGGCAACCCGTTGAACCTCCTTCGT
GCTAGGGATTGGGGCTTGCAATTCTTCCCCATGAACGAGGAATTCCCAGTAAGCGCGAGTCATCAG
CTCGCGTTGATTACGTCCCTGCCCTTTGTACACACCGCCCGTCGCTACTACCGATTGGATGGTTTA
GTGAGACCCTCGGATTGGTCCCGACGGTGGGCGACCGCCGTCGTGGTGCCGAGAAGACGGTCAAAC
TTGACTATCTAGAGGAAGTAAAAGTTCACTAAGCGGAGGAAAAGAAACTAACCAGGATTCCCCTAG
TAACGGCGAGTGAAGCGGGAAGAGCCCAGCACTGAATCCCCTGCATCGACTGTAGAGGGAACTGTA
GTGTTCGGGAGAGCCTCTGGGGCGACGGCGGCGTCCGAGTCCTCCTGATCGGGGTTACCCAGAGCG
GGTGTCAGGCCTCTACGGCGGCTTCCGTCGTCCCTCTGTGCCTCCTAGGAGTCGGGTTGTTTGAGA
ATGCAGCCCAAAGCGGGTGGTAAACTCCATCTAAGGCTAAATACTGGCACGAGACCGATAGCGGAC
AAGTACCGTGAGGGAAAGTTGAAAAGAACTTTGAAGAGAGAGTTCAAGAGTACGTGAAACCGTATA
GAGGCAAACGGATGGGACCGCAGTCGATCCGCGGGATTCAGCTTCTGCCGGGCGGGCTGCGGACCT
TCACGGGGAGTGCACTTTCCGCGGGGAGAGCGCCACGACCGCTTCTCGGGCCGTCAAGCTCCGGCG
GAAGGTAGCTCGAGTGTTATAGCCGCCGGTAGTGTCGGCCGGGGGGAGCGAGGAATGCCCGCCGGA
GACCGGTCGACTGGCGAGGACTGCTCGCAGTGCTCGCCGACTGCGCCGTTCGGGCCACCCAGGGTC
TGTGGCGAAGTCGGTTTCCCATCCGACC

>Atherospio_aestuarii_P865
CGCCTTTAGATTAAAGGTATATTCTGCCCTATGTAAATGGCCGCGGTACCCTGACCGTGCTAAGGT
AGCATAATAATTTGCCTCCTAATTAGAGGCTGGAATGAAGGAACTCACGAAAGTTTAACTGTTTCA
TTTTTACTAAAAACTATCATTCAAGTGAAGAGACTTGTATTTTATAAAAAGACAAAAAGACCCTAT
TGAGCTTTATTCTACTTATTACCCTGATAAAGAATTTTGTTGGGGCGACAAGAGAAGACTAAATCT
TCTCAACCTCCTAAAAAGATCCGAAGCGATCAAAAATCTAGCTACCCTAGGGATAACAGGCTAATC
TTCTTTGAGAGCCCAAATTGACAAGAAGGATTGGCACCTCGATGTTGGCTTAGGGTACCAAATAAA
TGCAGAAGTAAGTAGGTTTGTTCAACCTTTAATACCCTATATGCTTGTCTCAAAGACTAAGCCATG
CATGTCTAAGTACAAGCTCAACATGGTGAAACCGCGAATGGCTCATTAAATCAGTTATGGTTCCTT
AGATCGTACTATCCTACTTGGATAACTGTAGTAATTCTAGAGCTAATACATGCAATCAAGCTCCGA
CCTCGCGGGAGGAGCGCTTTTATTAGACCAAAACCAATGGGGGCGACTAACTTCTGGTGACTCTGG
ATAACTTTGAGCTGATCGCACGGCCGCGCCGGCGACGTATCTTTCAAATGTCTGCCCTATCAAGTT
TCGACGGTTGGTGACTTGCCAACCGTGCTGATAACGGGTAGCGGGGAATCAGGGTTCGATTCCGGA
GAGGGAGCATGAGAAACGGCTACCACATCCAAGGAAGGCAGCAGGCGCGCAAATTACCCACTCCTA
GCTCGGGGAGGTAGTGACGAAAAATAACAATACGGGACTCTTACGAGGCCCCGTAATTGGAATGAG
TACACTTTAAATCCTTTAACGAGGATCTATTGGAGGGCAAGTCTGGTGCCAGCAGCCGCGGTAATT
CCAGCTCCAATAGCGTATATTAAAGTTGTTGCAGTTAAAAAGCTCGTAGTTGGATCTCGGGTGCAG
GCTGGCGGTCCGCCTCGCGGCGGTTTACTGCCTCCTGCTCCTCTTGCCGGTTTCCCTTGGTGCTCT

TCGCTGGGGTGGCCGGGACGTTTACTTTGAAAAAATTAGAGTGTTCAAAGCAGGCCCTGAATACTG
GTGCATGGAATAATGGAATAGGACCTCGGATTTTGTTGGTTTTCGGAAGTCGAGGTAATGATTAAT
AGGGACGGCGGGGGCATTTGTATTGCGTCGTTAGAGGTGAAATTCTTGGATCGACGCAAGACAGAC
TACAGCGAAGGCGTTTGCCAAGCACGTCTTCTTTAATCAAGAGCGACAGTCAGAGGTTCGAAGACG
ATCAGATACCGTCGTAGTTCTGACCATAAACGATGCCAGCTAGGGATTCGCCGGAGTTGTCTGACT
CGGCGAGCACCTTCCGGGAAACCAAAGCTTTTGGGTTCCGGGGGAAGTATGGTTGCAAAGCTGAAA
CTTAAAGGAATTGACGGAAGGGCACCACCAGGAGTGGAGCCTGCGGCTTAATTTGACTCAACACGG
GGAAACTCACCCGGCCCGGACACCGTAAGGATTGACAGATTGAGAGCTCTTTCTTGATTCGGTGGG
TGGTGGTGCATGGCCGTTCTTAGTTGGTGGAGCGATTTGTCTGGTTAATTCCGATAACGAACGAGA
CTCTAGCCTATTAAATAGTTCGCCGATTCGTCGGCGAAACTTCTTAGAGGGACAAGTGGCGTTCAG
CCACGCGAGATTGAGCAATAACAGGTCTGTGATGCCCTTAGATGTTCGGGGCCGCACGCGCGCTAC
ACTGAAGGACTCAGCGTGTCTTCTCCTGGCCAGAAATGGTCGGGCAACCCGTTGAACCTCCTTCGT
GCTAGGGATTGGGGCTTGCAATTCTTCCCCATGAACGAGGAATTCCCAGTAAGCGCGAGTCATCAG
CTCGCGTTGATTACGTCCCTGCCCTTTGTACACACCGCCCGTCGCTACTACCGATTGGATGGTTTA
GTGAGACCCTCGGATTGGTCCCGACGGTGGGCGACCGCCGTCGTGGTGCCGAGAAGACGGTCAAAC
TTGACTATCTAGAGGAAGTAAAAGT---CTAAGCGGAGGAAAAGAAACTAACCAGGATTCCCCTAG
TAACGGCGAGTGAAGCGGGAAGAGCCCAGCACTGAATCCCCTGCATCGACTGTAGAGGGAACTGTA
GTGTTCGGGAGAGCCTCTGGGGCGACGGCGGCGTCCGAGTCCTCCTGATCGGGGTTACCCAGAGCG
GGTGTCAGGCCTCTACGGCGGCTTCCGTCGTCCCTCTGTGCCTCCTAGGAGTCGGGTTGTTTGAGA
ATGCAGCCCAAAGCGGGTGGTAAACTCCATCTAAGGCTAAATACTGGCACGAGACCGATAGCGGAC
AAGTACCGTGAGGGAAAGTTGAAAAGAACTTTGAAGAGAGAGTTCAAGAGTACGTGAAACCGTATA
GAGGCAAACGGATGGGACCGCAGTCGATCCGCGGGATTCAGCTTCTGCCGGGCGGGCTGCGGACCT
TCACGGGGAGTGCACTTTCCGCGGGGAGAGCGCCACGACCGCTTCTCGGGCCGTCAAGCTCCGGCG
GAAGGTAGCTCGAGTGTTATAGCCGCCGGTAGTGTCGGCCGGGGGGAGCGAGGAATGCCCGCCGGA
GACCGGTCGACTGGCGAGGACTGCTCGCAGTGCTCGCCGACTGCGCCGTTCGGGCCACCCAGGGTC
TGTGGCGAAGTCGGTTTCCCATCCGACC

>Atherospio_aestuarii_P866
CGCCTTTAGATTAAAGGTATATTCTGCCCTATGTAAATGGCCGCGGTACCCTGACCGTGCTAAGGT
AGCATAATAATTTGCCTCCTAATTAGAGGCTGGAATGAAGGAACTCACGAAAGTTTAACTGTTTCA
TTTTTACTAAAAACTATCATTCAAGTGAAGAGACTTGTATTTTATAAAAAGACAAAAAGACCCTAT
TGAGCTTTATTCTACTTATTACCCTGATAAAGAATTTTGTTGGGGCGACAAGAGAAGACTAAATCT
TCTCAACCTCCTAAAAAGATCCGAAGCGATCAAAAATCTAGCTACCCTAGGGATAACAGGCTAATC
TTCTTTGAGAGCCCAAATTGACAAGAAGGATTGGCACCTCGATGTTGGCTTAGGGTACCAAATAAA
TGCAGAAGTAAGTAGGTTTGTTCAACCTTTAATACCCTATATGCTTGTCTCAAAGACTAAGCCATG
CATGTCTAAGTACAAGCTCAACATGGTGAAACCGCGAATGGCTCATTAAATCAGTTATGGTTCCTT

AGATCGTACTATCCTACTTGGATAACTGTAGTAATTCTAGAGCTAATACATGCAATCAAGCTCCGA
CCTCGCGGGAGGAGCGCTTTTATTAGACCAAAACCAATGGGGGCGACTAACTTCTGGTGACTCTGG
ATAACTTTGAGCTGATCGCACGGCCGCGCCGGCGACGTATCTTTCAAATGTCTGCCCTATCAAGTT
TCGACGGTTGGTGACTTGCCAACCGTGCTGATAACGGGTAGCGGGGAATCAGGGTTCGATTCCGGA
GAGGGAGCATGAGAAACGGCTACCACATCCAAGGAAGGCAGCAGGCGCGCAAATTACCCACTCCTA
GCTCGGGGAGGTAGTGACGAAAAATAACAATACGGGACTCTTACGAGGCCCCGTAATTGGAATGAG
TACACTTTAAATCCTTTAACGAGGATCTATTGGAGGGCAAGTCTGGTGCCAGCAGCCGCGGTAATT
CCAGCTCCAATAGCGTATATTAAAGTTGTTGCAGTTAAAAAGCTCGTAGTTGGATCTCGGGTGCAG
GCTGGCGGTCCGCCTCGCGGCGGTTTACTGCCTCCTGCTCCTCTTGCCGGTTTCCCTTGGTGCTCT
TCGCTGGGGTGGCCGGGACGTTTACTTTGAAAAAATTAGAGTGTTCAAAGCAGGCCCTGAATACTG
GTGCATGGAATAATGGAATAGGACCTCGGATTTTGTTGGTTTTCGGAAGTCGAGGTAATGATTAAT
AGGGACGGCGGGGGCATTTGTATTGCGTCGTTAGAGGTGAAATTCTTGGATCGACGCAAGACAGAC
TACAGCGAAGGCGTTTGCCAAGCACGTCTTCTTTAATCAAGAGCGACAGTCAGAGGTTCGAAGACG
ATCAGATACCGTCGTAGTTCTGACCATAAACGATGCCAGCTAGGGATTCGCCGGAGTTGTCTGACT
CGGCGAGCACCTTCCGGGAAACCAAAGCTTTTGGGTTCCGGGGGAAGTATGGTTGCAAAGCTGAAA
CTTAAAGGAATTGACGGAAGGGCACCACCAGGAGTGGAGCCTGCGGCTTAATTTGACTCAACACGG
GGAAACTCACCCGGCCCGGACACCGTAAGGATTGACAGATTGAGAGCTCTTTCTTGATTCGGTGGG
TGGTGGTGCATGGCCGTTCTTAGTTGGTGGAGCGATTTGTCTGGTTAATTCCGATAACGAACGAGA
CTCTAGCCTATTAAATAGTTCGCCGATTCGTCGGCGAAACTTCTTAGAGGGACAAGTGGCGTTCAG
CCACGCGAGATTGAGCAATAACAGGTCTGTGATGCCCTTAGATGTTCGGGGCCGCACGCGCGCTAC
ACTGAAGGACTCAGCGTGTCTTCTCCTGGCCAGAAATGGTCGGGCAACCCGTTGAACCTCCTTCGT
GCTAGGGATTGGGGCTTGCAATTCTTCCCCATGAACGAGGAATTCCCAGTAAGCGCGAGTCATCAG
CTCGCGTTGATTACGTCCCTGCCCTTTGTACACACCGCCCGTCGCTACTACCGATTGGATGGTTTA
GTGAGACCCTCGGATTGGTCCCGACGGTGGGCGACCGCCGTCGTGGTGCCGAGAAGACGGTCAAAC
TTGACTATCTAGAGGAAGTAAAAGT-CACTAAGCGGAGGAAAAGAAACTAACCAGGATTCCCCTAG
TAACGGCGAGTGAAGCGGGAAGAGCCCAGCACTGAATCCCCTGCATCGACTGTAGAGGGAACTGTA
GTGTTCGGGAGAGCCTCTGGGGCGACGGCGGCGTCCGAGTCCTCCTGATCGGGGTTACCCAGAGCG
GGTGTCAGGCCTCTACGGCGGCTTCCGTCGTCCCTCTGTGCCTCCTAGGAGTCGGGTTGTTTGAGA
ATGCAGCCCAAAGCGGGTGGTAAACTCCATCTAAGGCTAAATACTGGCACGAGACCGATAGCGGAC
AAGTACCGTGAGGGAAAGTTGAAAAGAACTTTGAAGAGAGAGTTCAAGAGTACGTGAAACCGTATA
GAGGCAAACGGATGGGACCGCAGTCGATCCGCGGGATTCAGCTTCTGCCGGGCGGGCTGCGGACCT
TCACGGGGAGTGCACTTTCCGCGGGGAGAGCGCCACGACCGCTTCTCGGGCCGTCAAGCTCCGGCG
GAAGGTAGCTCGAGTGTTATAGCCGCCGGTAGTGTCGGCCGGGGGGAGCGAGGAATGCCCGCCGGA
GACCGGTCGACTGGCGAGGACTGCTCGCAGTGCTCGCCGACTGCGCCGTTCGGGCCACCCAGGGTC
TGTGGCGAAGTCGGTTTCCCATCCGACC

>Amphicorina_mobilis

TGCTTTCTGATAAAAAGTAATTCCTGCCCAGTGTAAACGGCCGCAGTACCCTGACTGTGCAAAGGT
AGCATAATCGTTTGCCTCTTAATTGGAGGCTTGTATGAACGGATTCACGAGCGTCTAGCTATATTT
ACCTT-ACGAAACCTAATATACCGGTGAAAAAGCCGGTATTAAACTGAAAGACAAGAAGACCCTAT
TGAGCTTTAACCAAACATTTTTTATAGTTCAAAGTTTAGTTGGGGCAACTAAAATTAATCAAATCC
TACGCCTTCTAATACCTGATCCT--AAGATATCAGAAATAGCTACCTTAGGGATAACAGGCTAATT
TCTTTTAAGAGTACAAATTAACAAAGAAGTTTGGCACCTCGATGTTGGCTTAGGGACCCTGTTTAG
TGCAGCTGCAGGTGGGCTTGTTCATCCCTTAATACCCT----------------------------
---ATTCATTAGACCGTCAC
AGCCTCGGTTGTCTGACGTGGATAACTGTGGTAATTCTAGAGCTAATACATGCATACAAGCTCCGA
CCTTCGGGGAAGAGCGCTTTTATTAGACCAAAACCCGCGGCGTCGGCCCCCTTTTGGTGACTCTGG
ATAACCTTGGGCTGATCGCACGGCCGCGCCGGCGACGAGTCTTTCAAGCGTATGCCCTATCAACTG
TCGATGGTACGTGCTATGCCTACCATGGTTTTTACGGGTAACGGGGAATCAGGGTTCGATTCCGGA
GAGGGAGCATGAGAAACGGCTACCACATCCAAGGAAGGCAGCAGGCGCGCAAATTACCCACTCCCG
ACACGGGGAGGTAGTGACGAAAAATAACAATACGGGACTCTTTCGAGGCCCCGTAATTGGAATGAG
TACACTCTAAATCCTTTAACGAGGATCTATTGGAGGGCAAGTCTGGTGCCAGCAGCCGCGGTAATT
CCAGCTCCAATAGCGTATATTAAAGTTGTTGCAGTTAAAAAGCTCGTAGTTGGATCTCGGGTCCGG
GCTGGCGGTCCCGTTCGCGCGGGC--ACTGCCTCCCG-----CCTACCGGCCTCCCTTGGTGCTCT
TCGCTGGGGTGGCCGGCGCGTTTACTTTGAAAAAATTAGAGTGTTCAAAGCAGGCCCTGAATACCG
ATGCATGGAATAATGGAATAGGACCTCGGATTTTGCTGGTTTCTGGATCTCGAGGTAATGATTAAG
AGGGACGGCGGGGGCGTTAGTATTACGGTGTTAGAGGTGAAATTCTTGGATCGCCGTAAGACTCAC
GGTTGCGAAAGCGTTCGCCAAGAACGTTTTCATTGATCAAGAACGAAAGTCAGAGGTTCGAAGACG
ATCAGATACCGTCGTAGTTCTGACCATAAACGATGCCAGCTAGGGATCGGCGGGCGTTGTGTGACT
CCGCCGGCACCTTCCGGGAAACCAAAGCTTTTGGGTTCCGGGGGAAGTATGGTTGCAAAGCTGAAA
CTTAAAGGAATTGACGGAAGGGCACCACCAGGAGTGGAGCCTGCGGCTTAATTTGACTCAACACGG
GAAAACTCACCCGGCCCGGACACCGTAAGGATTGACAGATTGAGAGCTCTTTCTTGATTCGGTGGG
TGGTGGTGCATGGCCGTTCTTAGTTGGTGGAGCGATTTGTCTGGTTAATTCCGATAACGAACGAGA
CTCTAGCCTATTAAATAGTTCGCGGTTGCGTCGGCGTAACTTCTTAGAGGGACAAGTGGCGTTTAG
CCACGCGAGATTGAGCAATAACAGGTCTGTGATGCCCTTAGATGTTCGGGGCCGCACGCGCGCTAC
ACTGAAGGAATCAACGTGTC-TCTCCCGGTCCGGCAGGTCCGGGTAACCCGTTGAACCTCCTTCGT
GCTAGGGATTGGGGCTTGTAACTGTTCCCCATGAACGAGGAATTCCCAGTAAGCGCGAGTCATAAG
CTCGCGTTGATTACGTCCCTGCCCTTTGTACACACCGCCCGTCGCTACTACCGATTGGATGATTTA
GTGAGGCCCTCGGATTGGTCCGCGCCGGGGGTCTTCGGAGGCACTGTGCCGAGAAGACGGCCAAAC
TTGATTATCTAGAGGAAGTAAAAGT-----------------------------------CCACAG
TAACGGCGAGTGAAGAGGGAAGAGCCCAGCACCGAATCCCCCGCCTTGCGGCGCTGGGGAACTGTG
GTGTTTGGGAGAGCCTCTGGTGCGTCGCCTGCGTCCGAGTCCTCCTGATTGGGGTCGCCCGTAGCG

GGTGTCAGGCCTCTACGGCGGCGGCGGCCGTGCCTCTGAGCCTCCCCGGAGTCGGGTTGTTTGAGA
ATGCAGCCCAAAGTGGGTGGTAAACTCCATCTAAGGCTAAATACCGACACGAGACCGATAGCGGAC
AAGTACCGTGAGGGAAAGTTGAAAAGAACTTTGAAGAGAGAGTTCAAGAGTACGTGAAACCGTTTA
GAGGCAAACGGATAGGACCGCAGTCCGCCCGCGGAATTCAACCTCGCCGGCGCGGTGCCTTCGGAT
CGTCCGTGGGCGCACTTTCCGCGGGGGGAGCGCCACGACCGGTTCCTGGGCCGTCGAGCCCCGGCG
GAAGGTAGCCGTGGTGTTACAGACGTCGGTGG--TGGGCCGGGGGGGACCGAGGATTCCCGCCGGG
GCGTGTCGCACTGGGCGGGACTGCTCGCAGTGCCGTCCGACCGCGGCTTCCTTGGCTGCCAGGGTC
TGTGGCGACGTCGGCACCCTATCCGACC

>Aonidella_cf_dayi
-------------------------------------TTAATGGCCGCGGTACCCTGACCGTGCAAAGGT
AGCATAATCATTTGCCTTTTAATTGGAGGCTAGTATGAATGGATATACGAGAGTAAAGTTGTCTCA
TTTTTAATAAAAATTAACCTTTGGGTGAAGAGGCCCAAATCTATAAAAAAGACGAAAAGACCCTAT
GGAGCTTTACTGCTACACTACACCTTAAAACCAGTTTAGTTGGGGCGACTAAGGAACATATAAAAC
TTCCTGCCCTCAAATAAGATCCAAAGTGA-TAAAAAATCAGCTACCCTAGGGATAACAGACTAATT
TTCCTCTAGAGCCCAAATTGACAGGAAAGATTGGCACCTCGATGTTGGCTTAAGACTCCCCCAAAA
TGCAGAAGTTGGTAGGTTTGTTCAACCTT---
---------------------TTAACATGGTGAAACCGCGAATGGCTCATTAAATCAGTTATGGTTCCTT
AGATCGTACAATCCTACTTGGATAACTGTAGTAATTCTAGAGCTAATACATGCAAGCAAGCTCCGA
CCTTCGGGAAAGAGCGCTTTTATTAGACCAAAACCAAT--GGGCAACCTCTCGTTGGTGACTCTGG
ATAACTTTGAGCTGATCGCATGGCCGAGCCGGCGACGTATCTTTCAAATGTCTGCCCTATCAACTT
TCGATGGTACGTGATATGCCTACCATGGTTGTAACGGGTAACGGGGAATCAGGGTTCGATTCCGGA
GAGGGAGCATGAGAAACGGCTACCACATCCAAGGAAGGCAGCAGGCGCGCAAATTACCCACTCCTG
GCACGGGGAGGTAGTGACGAAAAATAACAATACGGGACTCTTTCGAGGCCCCGTAATTGGAATGAG
TACACTTTAAATCCTTTAACGAGGATCTATTG--------------------------------------
----

--------------------------??
??
??
??
??
??
??
??
??
??
????????????????????????????

>Aonides_oxycephala

-GCATAATCATTTGCCTTTTAATTGGAGGCTAGTATGAATGGACACACGAGGGCTCTACTGTCTCC
TAACTATTATAAATTAGTCTTTAAGTGAAGAGACTTAAATATAATAAAAAGACGAAAAGACCCTAT
GGAGCTTTATTATGGTTATATGGTTTAAGTGTAATTCAGTTGGGGCGACTGAGGAAAAAATAAATC
TTCCTCTAAGAAAAATTGATCCGTAACGA-TAAAGATCTAGCTACCCTAGGGATAACAGGCTTATT
CTTCTTGAGAGTTCAAATCGACAGAAGAGA--

---------------------------------------GAATGGCTCATTAAATCAGTTATGGTTCCTT
AGATCGTACAATCCTACTTGGATAACTGTAGTAATTCTAGAGCTAATACATGCAAGCAAGCTCCGA
CCTCCGGGGAAGAGCGCACTTATTAGACCAAAACCAAT--GGGCAACCTCTCGTTGGTGACTCTGG
ATAACTTTGAGCTGATCGCATGGCCGAGCCGGCGACGTATCTTTCAAATGTCTGCCCTATCAACTT
TCGATGGTACGTGATATGCCTACCATTGTGATAACGGGTAACGGGGAATCAGGGTTCGATTCCGGA
GAGGGAGCATGAGAAACGGCTACCACATCCAAGGAAGGCAGCAGGCGCGCAAATTACCCACTCCTG
GCACGGGGAGGTAGTGACGAAAAATAACAATACGGGACTCTTTCGAGGCCCCGTAATTGGAATGAG
TACACTTTAAATCCTTTAACGAGGATCTATTGGAGGGCAAGTCTGGTGCCAGCAGCCGCGGTAATT
CCAGCTCCAATAGCGTATATWAAAGTTGTTGCAGTTAAAAAGCTCGTAGTTGGATCTCGGGCGCAG
GCTGGCGGTCCACCTCGCGGTGG-CTACTGCCTGTCCTGCCCCCTGCCGGTTTCCCCTGGTGCTCT

TCACTGGGGTGGCCGGAACGTTTACTTTGAAAAAATTAGAGTGTTCAAAGCAGGCCCTGAATAATG
GTGCATGGAATAATGGAATAGGACCTCGGATTTTGTTGGTTTTCGGAACTTGAGGTAATGATTAAG
AGGGACAGCGGGGGCATTCGTATTGCGGTGTTAGAGGTGAAATTCTTGGATCGCCGCAAGACGAAC
TACTGCGAAAGCATTTGCCAAGCATGTTTTCATTAATCAAGAACGAAAGTCAGAGGTTCGAAGACG
ATCAGATACCGTCGTAGTTCTGACCATAAACGATGCCAGCTAGGGATTCGCCGGAGTTGTATGACT
CGGCGAGCACCTTCCGGGAAACCAAAGCTTTTGGGTTCCGGGGGAAGTATGGTTGCAAAGCTGAAA
CTTAAAGGAATTGACGGAAGGGCACCACCAGGAGTGGAGCCTGCGGCTTAATTTGACTCAACACGG
GAAAACTCACCCGGCCCGGACACTGTAAGGATTGACAGATTGAGAGCTCTTTCTTGATTCGGTGGG
TGGTGGTGCATGGCCGTTCTTAGTTGGTGGAGCGATTTGTCTGGTTAATTCCGATAACGAACGAGA
CTCTAGCCTATTAAATAGTTCGCCGATACGTCGGCGCAACTTCTTAGAGGGACAAGTGGCGTTTAG
CCACGCGAGATTGAGCAATAACAGGTCTGTGATGCCCTTAGATGTTCGGGGCCGCACGCGCGCTAC
ACTGAAGGAATCAGCGTGTGTTCTCCTGGCCAGAAATGGTCGGGTAACCCGTTGAACCTCCTTCGT
GCTAGGGATTGGGGCTTGCAATTCTTCCCCATGAACGAGGAATTCCCAGTAAGCGCGAGTCATCAG
CTCGCGTTGATTACGTCCCTGCCCTTTGTACACACCGCCCGTCGCTACTACCGATTGGATGGTTTA
GTGAGACCCTCGGATTGGTCCCGACGGTGGGCAACCGCCGTCGTGGCGCCGAAAAGACGGTCAAAC
TTGACTATCTAGAGGAAGTAAAAGT-CACTAAGCGGAGGAAAAGAAACTAACCAGGATTCCCCTAG
TAACGGCGAGTGAAGCGGGAAGAGCCCAGCACCGAATCCCCTGCATTGACTGTAGCGGGACCTGTG
GTGTTTGGGAGAGCCTCTGGGGCGCCGGCGGCGTCCGAGTCCTCCTGATCGGGGTCACCCAGAGCG
GGTGTCAGGCCTCTACGGCTTCCGCCGTCGTCCCTCTGTGCCTCCTAGGAGTCGGGTTGTTTGGGA
ATGCAGCCCAAAGTGGGTGGTAAACTCCATCTAAGGCTAAATACTGGCACGAGTCCGATAGCGGAC
AAGTACCGTGAGGGAAAGTT--

>Aurospio_dibranchiata
----------------------------------GTGTTAACGGCCGCGGTACCCTGACCGTGCTAAGGT
AGCATAATCATTTGCCTTTTAATTGGAGGCTTGTATGAACGGACACACGAAAACGCAACTTTCTTA
TCGAGGATAAAAATTAACCTTCAAGTGAAGAGACTTGAGTATCATAAAAAGACGAAAAGACCCTAT
GGAGCTTTACTTAAATTTTTTTTTACTAAAAAAGTTTAGTTGGGGCGACTAAGGAGCAACACCACC
CTCCTACTGACAAAAAAGATCCAACGTGA-TAAAAATCCAGCTACCCTAGGGATAACAGGCTAATC
CTCCTTAAGAGCTCAAATTGACAGGAGGGCTTGGCACCTCGATGTTGGCTTAAGATCTCTACTAAG
TGCAGCAGCTAGTAGGTTTGTTCAACCTTTAAAATCTTATATGCTTGTCTCAAAGACTAAGCCATG
CATGTCTAAGTACAAGCTTAACATGGTGAAACCGCGAATGGCTCATTAAATCAGTTATGGTTCCTT

AGATCGTACAATCCTACTTGGATAACTGTAGTAATTCTAGAGCTAATACATGCAAGCAAGCTCCGA
CCTTCGGGAAAGAGCGCTTTTATTAGACCAAAACCAAT--GGGCAACCTCTCATTGGTGACTCTGG
ATAACTTTGAGCTGATCGCATGGCCGAGCCGGCGACGTATCTTTCAAATGTCTGCCCTATCAACTT
TCGACGGTACGTGATATGCCTACCGTGGTTGTAACGGGTAACGGGGAATCAGGGTTCGATTCCGGA
GAGGGAGCATGAGAAACGGCTACCACATCCAAGGAAGGCAGCAGGCGCGCAAATTACCCACTCCTG
GCACGGGGAGGTAGTGACGAAAAATAACAATACGGGACTCTTTCGAGGCCCCGTAATTGGAATGAG
TACACTTTAAATCCTTTAACGAGGATCTATTGGAGGGCAAGTCTGGTGCCAGCAGCCGCGGTAATT
CCAGCTCCAATAGCGTATATTAAAGTTGTTGCAGTTAAAAAGCTCGTAGTTGGATCTCGGGAGCAG
GCCGGCGGTCCGCCTCGCGGCGG-TTACTGCCTGTCCTGCCCCCTGCCGGTTTCCCCCGGTGCTCT
TCACTGGGGTGGCCGGAACGTTTACTTTGAAAAAATTAGAGTGTTCAAAGCAGGCCCTGAATAATG
GTGCATGGAATAATGGAATAGGACCTCGGATTTTGTTGGTTTTCGGAACTTGAGGTAATGATTAAG
AGGGACAGCGGGGGCATTCGTATTACGCTGTTAGAGGTGAAATTCTTGGATCGGCGTAAGACGAAC
TACTGCGAAAGCATTTGCCAAGAATGTTTTCATTAATCAAGAACGAAAGTCAGAGGTTCGAAGACG
ATCAGATACCGTCGTAGTTCTGACCATAAACGATGCCAGCTAGGGATTCGCCGGAGTTGTATGACT
CGGCGAGCACCTTCCGGGAAACCAAAGCTTTTGGGTTCCGGGGGAAGTATGGTTGCAAAGCTGAAA
CTTAAAGGAATTGACGGAAGGGCACCACCAGGAGTGGAGCCTGCGGCTTAATTTGACTCAACACGG
GGAAACTCACCCGGCCCGGACACTGTAAGGATTGACAGATTGAGAGCTCTTTCTTGATTCGGTGGG
TGGTGGTGCATGGCCGTTCTTAGTTGGTGGAGCGATTTGTCTGGTTAATTCCGATAACGAACGAGA
CTCTAGCCTATTAAATAGTTCGCCGATTCGTCGGCGCAACTTCTTAGAGGGACAAGTGGCGTTTAG
CCACGCGAGATTGAGCAATAACAGGTCTGTGATGCCCTTAGATGTTCGGGGCCGCACGCGCGCTAC
ACTGAAGGAATCAGCGTGTGTTCTCCTGGCCCGAAAGGGTCGGGTAACCCGTTGAACCTCCTTCGT
GCTAGGGATTGGGGCTTGCAATTATTCCCCATGAACGAGGAATTCCCAGTAAGCGCGAGTCATCAG
CTCGCGTTGATTACGTCCCTGCCCTTTGTACACACCGCCCGTCGCTACTACCGATTGGATGGTTTA
GTGAGACCCTCGGATTGGTCCCGACGGTGGGCAACCACCGTCGTGGCGCCGAAAAGACGGTCAAAC
TTGACTACCTAGAGGAAGTAAAAGT???????????????????????????????????
??
??
??
??
??
??
??
??
??
??
?????????????????????????

```
>Aurospio_foodbancsia
CGCCACAGGATCTGAGGTCCATCCTGCCCAGTGTTAACGGCCGCGGTACCCTGACCGTGGTAAGGT
AGCATAATCATTTGCCTTTTAATTGGAGGCTAGTATGAATGGATACACGAGGGTATAGTTGTCTCC
TTTATGCTGTAAATTATCCTTTAGGTGAAGAGACCTAAATACTATAAAAAGACGAAAAGACCCTAT
GGAGCTTTACTTAACTTGTATAAAAGAGAAAAAGTTCAGTTGGGGCGACTAAGGAGCAAAAAAARC
CTCCTCTTATTACAAAAGATCCACCGTGA-TATAGATCCAGCTACCCTAGGGATAACAGGCTAATC
CTCCTCGAGAGCCCAAATTGACAGGAGGGGTCCCCACCTCGATGTTGGCTTAAGGTCCCTCCAAGG
TGCAGCTGCAAGTAGGTTTGTTCACCCTTTAAAACCTTATATGCTTGTCTCAAAGACTAAGCCATG
CATGTTTAAGTACCAGCCTAACAAGGTGAAACCGCGAATGGCTCATTAAATCAGTTATGGTTCCTT
AGATCGTACAATCCTACTTGGATAACTGTAGTAATTCTAGAGCTAATACATGCAAGCAAGCTCCGA
CCTTCGGGAAAGAGCGCTTTTATTAGACCAAAACCAAT--GGGCAACCTCTCGTTGGTGACTCTGG
ATAACTTTGAGCTGATCGCACGGCCGAGCCGGCGACGTATCTTTCAAATGTCTGCCCTATCAACTT
TCGACGGTACGTGATATGCCTACCGTGGTTGTAACGGGTAACGGGGAATCAGGGTTCGATTCCGGA
GAGGGAGCATGAGAAACGGCTACCACATCCAAGGAAGGCAGCAGGCGCGCAAATTACCCACTCCTG
GCACGGGGAGGTAGTGACGAAAAATAACAATACGGGACTCTTTCGAGGCCCCGTAATTGGAATGAG
TACACTTTAAATCCTTTAACGAGGATCTATTGGAGGGCAAGTCTGGTGCCAGCAGCCGCGGTAATT
CCAGCTCCAATAGCGTATATTAAAGTTGTTGCAGTTAAAAAGCTCGTAGTTGGATCTCGGGAGCAG
GCCGGCGGTCCGCCTCGCGGCGG-TTACTGCCTGTCCTGCCCCCTGCCGGTTTCCCCCGGTGCTCT
TCGCTGGGGTGGCCGGAACGTTTACTTTGAAAAAATTAGAGTGTTCAAAGCAGGCCCTGAATAATG
GTGCATGGAATAATGGAATAGGACCTCGGATTTTGTTGGTTTTCGGAACTTGAGGTAATGATTAAG
AGGGACAGCGGGGGCATTCGTATTACACTGTTAGAGGTGAAATTCTTGGATCGGTGTAAGACGAAC
TACTGCGAAAGCATTTGCCAAGAATGTTTTCATTAATCAAGAACGAAAGTCAGAGGTTCGAAGACG
ATCAGATACCGTCGTAGTTCTGACCATAAACGATGCCAGCTAGGGATTCGCCGGAGTTGTATGACT
CGGCGAGCACCTTCCGGGAAACCAAAGCTTTTGGGTTCCGGGGGAAGTATGGTTGCAAAGCTGAAA
CTTAAAGGAATTGACGGAAGGGCACCACCAGGAGTGGAGCCTGCGGCTTAATTTGACTCAACACGG
GGAAACTCACCCGGCCCGGACACTGTAAGGATTGACAGATTGAGAGCTCTTTCTTGATTCGGTGGG
TGGTGGTGCATGGCCGTTCTTAGTTGGTGGAGCGATTTGTCTGGTTAATTCCGATAACGAACGAGA
CTCTAGCCTATTAAATAGTTCGCCGATTCGTCGGCGCAACTTCTTAGAGGGACAAGTGGCGTTTAG
CCACGCGAGATTGAGCAATAACAGGTCTGTGATGCCCTTAGATGTTCGGGGCCGCACGCGCGCTAC
ACTGAAGGAATCAGCGTGTGTTCTCCTGGCCAGAAATGGTCGGGTAACCCGTTGAACCTCCTTCGT
GCTAGGGATTGGGGTTTGCAATTATCCCCCATGAACGAGGAATTCCCAGTAAGCGCGAGTCATCAG
CTCGCGTTGATTACGTCCCTGCCCTTTGTACACACCGCCCGTCGCTACTACCGATT-GATGGTTTA
GTGAGACCCTCGGATGGGTCCCGACGGTGGGCAACCGCCGTCGTGGCGCCGAAAAGACGGTC-AAC
TTGACTATCTAGAGGAAGTAAA---?????????????????????????????????????????
??????????????????????????????????????????????????????????????????
??????????????????????????????????????????????????????????????????
```

??
??
??
??
??
??
??
????????????????????????????

>Boccardia_proboscidea
CGCCTCTAGATTAGAGGTCCATTCTGCCCTATGTAAATGGCCGCGGTAGCCTGACCGTGCTAAGGT
AGCATAATCATTTGCCGTTTAATTGATGGCTGGAATGAATGAATATACGTAAGCTCAACTGTCTCC
TTTTTAATAAAAATTGACCCCCAAGTGAAGAGACTTGGATTGACCAAAAAGACGAAAAGACCCTAT
AGAGCTTCACCTGACCTACTCGTCCAAGAGAAGGTTTAGTTGGGGCAACTAGAGAGTACTAAAACC
TCTCTTCCAAAAAATTAGATCCGACACGACAGCAGAGAAAGCTACCTTAGGGATAACAGGCTAATC
CTCTTTGAGAGACCATATTGACAAGAGGGGTTGGCACCTCGATGTTGGCTCAAGGTTACAGCTTGA
TGCAGCAGTTAGTGGGTTTGTTCACCCTTTAAATCCTTATATGCTTGTCTCAAAGACTAAGCCATG
CATGTCTAAGTATAAGCTCTACACGGTGAAACCGCGAATGGCTCATTAAATCAGTTATGGTTCCTT
AGATCGTAAAATCCTACTTGGATAACTGTAGTAATTCTAGAGCTAATACATGCACCTGAGCTCCGA
CCCTCGGGAAAGAGCGCTTTTATTAGACCAAAACCAATGGCTCGCGCCAACCTTTGGTGACTCTGG
ATAACTTC-AGCCGATCGCACGGCCGCGCCGGCGACGTATCTTTCAAATGTCTGCCCTATCAAGTT
TCGATGGTTGGTGACTTGCCAACCATGCTTATAACGGGTAGCGGGGAATCAGGGTTCGATTCCGGA
GAGGGAGCATGAGAAACGGCTACCACATCCAAGGAAGGCAGCAGGCGCGCAAATTACCCACTCCTA
GCTCGGGGAGGTAGTGACGAAAAATAACAATACGGGACTCTTACGAGGCCCCGTAATTGGAATGAG
TACACTTTAAATCCTTTAACGAGGATCTATTGGAGGGCAAGTCTGGTGCCAGCAGCCGCGGTAATT
CCAGCTCCAATAGCGTATATTAAAGTTGTTGCAGTTAAAAAGCTCGTAGTTGGATCTCGGGGGCGG
TCCGGCGGTCCTCCTCGCGGAGGTT-ACTGTC-ACTGCTCTCCTCACCGGCTTCCCCGGGTGCTCT
TCGCTGAGGCGGCCGGAATGTTTACTTTGAAAAAATTAGAGTGTTCAAAGCAGGCCCTGAATATTG
GCGCATGGAATAATGGAATAGGACCTCGGATTTTGTTGGTTTTCGGAAGTCGAGGTAATGATTAGT
AGGGACGACGGGGGCATTCGTATTGCGCGGTTAGAGGTGAAATTCTTGGATCCGCGCAAGACGAAC
TACAGCGAAGGCGTCTGCCAAGTGCGTCTTCATTAATCAAGAGCGACAGTCAGAGGTTCGAAGACG
ATCAGATACCGTCGTAGTTCTGACCATAAACGATGCCAGCTAGGGATGGGCGGACGTTGCACGACT
CCGCCCGGACCTTCCGGGAAACCAAAGCTTTTGGGTTCCGGGGGAAGTATGGTTGCAAAGCTGAAA
CTTAAAGGAATTGACGGAAGGGCACCACCAGGAGTGGAGCCTGCGGCTTAATTTGACTCAACACGG
GGAAACTCACCCGGCCCGGACACCGTAAGGATTGACAGATTGAAAGCTCTTTCTTGATTCGGTGGG
TGGTGGTGCATGGCCGTTCTTAGTTGGTGGAGCGATTTGTCTGGTTAATTCCGATAACGAACGAGA

CTCTAGCCTGCTAAATAGTTCGTCGGTTAGCCGGCGTAACTTCTTAGAGGGACAAGTGGCGTTCAG
CCACGCGAGATTGAGCAATAACAGGTCTGTGATGCCCTTAGATGTTCGGGGCCGCACGCGCGCTAC
ACTGAAGGACTCAGCTGGT--TCTCCTGGCCTGAAAAGGTCGGGCAAACCGTTGAACCTCCTTCGT
GCTAGGGATTGGGGCTTGCAATTATTCCCCATGAACGAGGAATTCCCAGTAGGCGCGAGTCATCAG
CTCGCGTCGATTACGTCCCTGCCCTTTGTACACACCGCCCGTCGCTACTACCGATCGGATGGTTTA
GTGAGACCCTCGGACTGGCCCCGTCGGCAGGCAACTGCTGACGTGTTGCCGGGAAGAAGGTCGAAC
TTGACTATCTAGAGGAAGTAAAAGTTCACTAAGCGGAGGAAAAGAAACTAACCAGGATTCCCTTAG
TAACGGCGAGTGAAACGGGAAGGGCCCAGCACCGAATCCCCTGCTTTT--AGTAGCGGGAACTGTG
GTGTTTAGGAGAGCCTGTGGAGCTGCGG-AGCGTCCGAGTCCTTATGAAAGGGGGCGGCCAGAGCG
GGTGTCAGTCCTCTAGGACGCCCCGTCCAGTTGCTCAGTACCTCCTCGGAGTCGGGTTGTTTGAGA
TTGCAGCCCAAAGCGGGTGGTAAACTCCATCTAAGGCTAAATACTGGCACGAGACCGATAGCAAAC
AAGTACCGTGAGGGAAAGTTGAAAAGAACTTTGAAGAGAGAGTTCAAGAGTACGTGAAACCGTATA
GAAGTAAACGGATGGGACCGCAGTGGACCGACGGGATTCAGTTCTTTC-GGGCGGATCGGAGACCT
TCACCGGGAATGCACTTTCCGTCGGGACAGCGCCACGACCGGTTCTTGGGCCGCCAGGCCCGGGGG
GAAGGTAGCTCGAGTGTTATAGCCCTCCGGTGTTCCGGCCGGGGGGACCGAGGAATACCCGCCGGA
GACTGGTGGACTGGCGAGGACTGCTCGCAGTGCTCGTCGACCGCGCTGTTCGGGCCACCCAGGGTC
TGTGGCGAAGTTGGCTACCCATCCGACC

>Dipolydora_bidentata
CGCCTCCAGATTGGAGGTCCATTCTGCCCTATGTAAATGGCCGCGGTAGCCTGACCGTGCTAAGGT
AGCATAATCATTTGCCGTTTAATTGGTGGCTGGAATGAATGAATATACGAGAGCTCTACTGTCTCA
TCTCTAATAAAAATTGACCTTCAGGTGAAGAGACCTGAATTCCTTAAAAAGACGAAAAGACCCTAT
AGAGCTTTACCTAAATTATTCTTCAAGACAAAGGTTTAGTTGGGGCAACTAGAGAGAACTAAAACC
TCTCTTTCTAAAAAATTGATCCGTTACGATATCAGAAAAAGCTACCTTAGGGATAACAGGCTAATC
CTCTTTAAGAGACCATATTGACAAGAGGGTTTGGCACCTCGATGTTGGCTCAAGGTTACAGTCTGA
TGCAGCAGTAAGTAGGTTTGTTCACCCTTTAAATCCTT----------------------------
------------TAAGCTCTACATGGTGAAACCGCGAATGGCTCATTAAATCAGTTATGGTTCCTT
AGATCTTAAAATCCTACTTGGATAACTGTAGTAATTCTAGAGCTAATACATGCACCTGAGCTCCGA
CCCGAGGGAAAGAGCGCTTTTATTAGACCAAAACCAATGGTTCGCGCCAACCTTTGGTGACTCTGG
ATAACTTC-AGCTGATCGCAAGGCCGAGCCGGCGACGTATCTTTCAAATGTCTGCCCTATCAAGTT
AAGATGGTTGGTGACTTGCCAACCATGCTCGTAACGGGTAGCGGGGAATCAGGGTTCGATTCCGGA
GAGGGAGCATGAGAAACGGCTACCACATCCAAGGAAGGCAGCAGGCGCGCAAATTACCCACTCCTG
GATCGGGGAGGTAGTGACGAAAAATAACAATACGGGACTCTTACGAGGCCCCGTAATTGGAATGAG
TACACTTTAAATCCTTTAACGAGGATCTATTGGAGGGCAAGTCTGGTGCCAGCAGCCGCGGTAATT
CCAGCTCCAATAGCGTATATTAAAGTTGTTGCAGTTAAAAAGCTCGTAGTTGGATCTCGGGAGCGG
TCCGGCGGTCCTCCGCAAGGAGGTTCACTGCCCGCCGCTTCCCTCACCGGCTTGCCCGGGTGCTCT

TCGTTGGGTTGGCCGGAATGTTTACTTTGAAAAAATTAGAGTGTTCAAAGCAGGCCCTGAATATTG
GCGCATGGAATAATGGAATAGGACCTCGGATTTTGTTGGTTTTCGGAAGTCGAGGTAATGATTAGT
AGGGACGACGGGGGCATTCGTATTGCACGGTTAGAGGTGAAATTCTTGGATCCGTGCAAGACGAAC
TACA--

-------------------------TCACTAAGCGGAGGAAAAGAAACTAACCAGGATTCCCTTAG
TAACGGCGAGTGAAACGGGAAATGCCCAGCACCGAATCCCCTGCCCGTC-GGCAGCGGGAACTGTG
GTGTTTAGGAGAGCCTGTGGAGCGACGG-AGCGTCCGAGTCCTTATGAAAGGGGGCAGCCAGAGCG
GGTGTTAGTCCTCTAGGACGCCCCGTCCCGTTGCTCAGTACCTCCTCGGAGTCGGGTTGTTTGTGA
ATGCAGCCCTAAGTGGGTGGTAAACTCCATCTAAGGCTAAATACTGGCACGAGACCGATAGCAAAC
AAGTACCGTGAGGGAAAGTT--

>Dispio_remanei
??
??
??
??
??
??
??????????????????????????????????????----------------------------
----

---AATGAAA
CTTAAAGGAATTGACGGAAGGGCACCACCAGGAGTGGAGCCTGCGGCTTAATTTGACTCAACACGG
GGAAACTCACCCGGCCCGGACACCGTAAGGATTGACAGATTGAGAGCTCTTTCTTGATTCGGTGGG
TGGTGGTGCATGGCCGTTCTTAGTTGGTGGAGCGATTTGTCTGGTTAATTCCGATAACGAACGAGA
CTCTAGCCTATTAAATAGTTCGCCGACTCGTCGGCGGAACTTCTTAGAGGGACAAGTGGCGTTCAG
CCACGCGAGATTGAGCAATAACAGGTCTGTGATGCCCTTAGATGTTCGGGGCCGCACGCGCGCTAC
ACTGAAGGACTCAGCGTGTCTTCTCCTGGCCAGAAATGGTCGGGCAACCCGTTGAACCTCCTTCGT
GCTAGGGATTGGGGCTTGTAATTCTTCCCCATGAACGAGGAATTCCCAGTAAGCGCGAGTCATCAG
CTCGCGTTGATTACGTCCCTGCCCTTTGTACACACCGCCCGTCGCTACTACCGATTGGATGGTTTA
GTGAGACCCTCGGATTGGTCCCGGCGGTGGGCGACTGCCGCCGCGGTGCCGAGAAGACGGTCAAAC
TTGACTATCTAGAGGAAGTAAAAGTTCACTAAGCGGAGGAAAAGAAACTAACAAGGATTCCCCTAG
TAACGGCGAGTGAAGCGGGAAGAGCCCAGCACCGAATCCCCTGCATTGACTGTAGAGGGAACTGTG
GTGTGTGGGAGAGCCTCTGGCGTCGCCGCCTCGTCCGAGTCCTCCTGATCGGGGTTACCCAGAGCG
GGTGTTAGGCCTCTACGGCGCGGGCGGCGGCGTCTCTGAGCCTCCTCGGAGTCGGGTTGTTTGAGA
ATGCAGCCCAAAGTGGGTGGTAAACTCCATCTAAGGCTAAATACTGGCACGAGGCCGATAGCGGAC
AAGTACCGTGAGGGAAAGTTGAAAAGAACT------------------------------------
----

>Glandulospio_orestes
------------------CAATTCTGCTCGCTGTAAAGGGCCGCGTTAGCGTGAGCGTGCAAAGGT
AGCATAATAATTTGCCTAATAATTGTAGGC-AGGATCAACGAACGTACGCGGGCAGTGCTGTCTCG
GATTAGTTTGAAGTTAGCCCAAGGGTGAATAGACCCCTATAGCATTAAAAGACAAAAAGACCCTAC
-GAGCTTTATTCTATCTAACTGATCAGTGGGGGAGTTGGTTGGGGCAACTGGAGACCTCTAAAAAT
TCTACCCTAGTATA--TGAACCACGGTGA-CAAGTGTGGAGCTACCATAGGGATAACAGGCCAATC
CCCGTTAAGAGACCGTATTGACATGGGGGTTTGGTACCTCGATGTTGGCTTAAGGTGGCTGTGTGA
TGCAGGCGTAAGTGGGCTTGTTCTGCCCT-------------------------------------
--TTATGGTTCCTT
AGATCAT-CTATCCTACTTGGATAACTGTAGTAATTCTAGAGCTAATACATGCACGGAAGCTCCGA
CCCGAGGGGAAGAGCGCTTTTATTAGACCAAAATCAATGGGGAAGCTCAACGTTTGGTGACTCTGG
ATAACTTC-AGCTGATCGCACGGCCGCGCCGGCGACGTATCTTTCAAATGTCTGCCCTATCAAGTT
TCGACGGTTGGTGACCTGCCAACCGTGCTGATAACGGGTAGCGGGGAATCAGGGTTCGATTCCGGA
GAGGGAGCATGAGAAACGGCTACCACATCCAAGGAAGGCAGCAGGCGCGCAAATTACCCACTCCTA
GCTCGGGGAGGTAGTGACGAAAAATAACAATACGGGACT---------------------------
----

-------------------------???
??
??

??
??
??
??
??
??
??
????????????????????????????

>Laonice_sp_VR_2006

-GCATAATCATTTGCCTCTTAATTGGTGGCTAGTATGAATGGACACACGAAAATAAGACTGTCTCC
TTAATAATAAAAATTAACCCCCACGTGAAGAGGCGTGGATACTACAGAAAGACAAAAAGACCCTAT
GGAGCTTTATTTCACCGTAATACATAACCTAAAATTCAGTTGGGGCGACTGAGGAACAAACAAACC
TTCCTTAAAACAAAAAAGATCCGCTATGC-AACAGACTAAGCTACCCTAGGGATAACAGGCTAATT
CTTCTTAAGAGCCCAAATTGACAGAAGAGA------------------------------------
----
------TGCGTACGAGCCTCGAACAGCGAAACCGCGAATGGCTCATTAAATCAGTTATGGTTCATT
AGAGCTTTGCCAGTTACATGGATAACTGTAGTAATTCTAGAGCTAATACATGCCACCGAGCTCCGA
CCCCGGGGGAAGAGCGCACTTATTAGACCACGATCAAC--GGGCAACCAGCATCTGGTGACTCTGG
ATAACTTGAAGCGGATCGCAGGGCCGCGCCGGCGATTCATCTTACAACTGTCTGACCTATCAA--C
TAGTAGGTAGGGTATACGCCTACCTAGGTGATGACGGGTAACGGGGAATCAGGGTTCGATTCCGGA
GAGGGAGCATGAGAAACGGCTACCACATCCAAGGAAGGCAGCAGGCACGCAAATTACCCACTCCTG
GCAAGGGGAGGTAGTGACGAAAAATAACAATACGGGACTCTTTCGAGGCCCCGTAATTGGAATGAG
TACGATGTAAAATCCCTAACGAGGAACTAATGGAGGGCAAGTCTGGTGCCAGCAGCCGCGGTAATT
CCAGCTCCATTAGCGTATATTAAAGCTGTTGCAGTTAAAAAGCTCGTAGTTGGATCTGGGGAGCAG
GCTGGCGGACCGCTTCGCTGCGGG--ACTGCCTCCTGCCCT-TCTGCCGGGCATGCAGGTTGCTCT
TCGATGCGTGGCCCGGCACTCTTACTTTGAAAAAATTAGAGTGCTCCAAACAGGCCCTGAATAATG
CTGCATGGAATAATGGAACAGGACCTCGGGTGAAGTTGGTCGTCGGAACCCGAAGTAATGATCAAT
AGGGGCTGCGGGGGCATCAGTATTCCAGGGCGAGAGGTGAAATTCTATGACCCTTGGAAGACTCCC
AAAAGCGAAAGCATTTGCCAAGAATGCCTTCTTTAATCAAGAACGAAAGTCAGAGGATCGAAGACG
ATCAGATACCGTCGTAGTTCTGACCGTAAACGCTGGCGTCTGGTGATTGTCGGGCGTGGGCGACCC
TGACAGCAACCCCTCGGGAAACCAAAGATTTTGGCTTCCGGGGGGAGTATGGTTGCAAAGCTGAAA
CTTAAAGGAATTGACGGAAGGGCACCACCAGGAGTGGAGCCTGCGGCTTAATTTGACTCAACACGG
GGAACCTCACCCGGCCCGGACACCGTAAGGATTGACAGATTGAGAGCTCTTTCTTGATTCGGTGGG
TGGTGGTGCATGGCCGTTCTTAGTTGGTGGAGTGATTTGTCTGGTTAATTCCGATAACGAACGAGA

CTCTAGCCTGCTAAATAGTTCGCCGATGCGTCGGCGCAACTTCTTAGAGGGACAGGCGGCTTTTAG
CCGCATGAGATTGAGCAATAACAGGTCTGTGATGCCCTTAGATGTCTGGGGCCGCACGCGCGCTAC
ACTGAAGGGATCAGCGTGCGT--CTCCTGTCCGAAAGGATCGGGTAACCCGTTGAACCTCCTTCGT
GCTAGGGATAAGGGCCTGAAATTGCTCCCTTTGAACGAGGAATTCCCAGTAAGCGCAAGTCATCAG
CTTGCGTTGATTACGTCCCTGCCCTTTGTACACACCGCCCGTCGCTACTACCGATTGGATGGTTTA
GTGAGACCCTCGGATCGACGCCCGGCCGGCTCGTTCGGC----GGGAGTTGAGAAGAAGGTCAAAC
TTGAAT-------------------TCACTAAGCGGAGGAAAAGAAACTAACCAGGATTCCCCTAG
TAACGGCGAGTGAAGAGGGAAGAGCCCAGCACCGAATCCCCTACCTCGCGGGTTGCGGGAACTGTG
GTGTTTGGGAGAGCCTCTGAGGCGCCGGCGGCTTCCGAGTCTCCTTGACGGGGGTCGCCCAGAGCG
GGTGTCAGGCCTCTAGGTTGCCCGCCGCCGTCTCTCTGAGCCTCCCCGGAGTCGGGTTGTTTGAGA
TTGCAGCCCAAAGTGGGTGGTAAACTCCATCTAAGGCTAAATACTGGCACGAGACCGATAGCAAAC
AAGTACTGTGAAGGAAAGTT--

>Malacoceros_cf_indicus
CGCCTCTAGATTAGAGGTCCATCCTGCCCAATGTAAATGGCCGCGGTACCCTGACCGTGCAAAGGT
AGCATAATAATTTGCCCCCTAATTAGGGGCTAGAATGAATGGACAAACGAGAGTTATACTGTCTCA
CTATAAATAAAAATTAACCTTCAAGTGAAGAGGCTTG-ACAAGAAAAAAAGACAAGAAGACCCTAT
TGAGCTTCATTTTATACCCCACAAGAGGAAAAAATTTAGTTGGGGCGACTAAGGAGCATAAAAACC
CTCCTTACTCCACAAAAGATCCGTAGCGATAGAAGAACTAGCTACCATAGGGATAACAGGCTAATC
CTTCTCGAGAGCCCAAATTGACAGAAGGGATTGGCACCTCGATGTTGGCTTAGGAAAACACCAAAA
TGCAGAAGTCAGCGGGTTTGTTCAACCCTTAATATCCTATATGCTTGTCTCAAAGACTAAGCCATG
CATGTCTAAGTACAAGCTCTACACGGTGAAACCGCGAATGGCTCATTAAATCAGTTATGGTTCCTT
AGATCGTACAATCCTACTTGGATAACTGTAGTAATTCTAGAGCTAATACATGCAAGCAAGCTCCGA
CCTCCGGGAAGGAGCGCTTTTATTAGACCAAAACCAATGTGGGCAACCTCTCGTTGGTGACTCTGG
ATAACTTTGAGCTGATCGCATGGCCGAGCCGGCGACGTATCTTTCAAATGTCTGCCCTATCAACTT
TCGATGGTACGTGATATGCCTACCATGGTAGTAACGGGTAGCGGGGAATCAGGGTTCGATTCCGGA
GAGGGAGCATGAGAAACGGCTACCACATCCAAGGAAGGCAGCAGGCGCGCAAATTACCCACTCCTG
GCACGGGGAGGTAGTGACGAAAAATAACAATACGGGACTCTTTCGAGGCCCCGTAATTGGAATGAG
TACACTTTAAATCCTTTAACGAGGATCTATTGGAGGGCAAGTCTGGTGCCAGCAGCCGCGGTAATT
CCAGCTCCAATAGCGTATATTAAAGTTGTTGCAGTTAAAAAGCTCGTAGTTGGATCTCGGGAGCAG
GCTGGCGGTCCGCCTCGCGGCGGTTTACTGCCCGTCCTGCCCCCAGCCGGTTTCCCCTGGTGCTCT

TGGTTGGGGTGGCCGGAACGTTTACTTTGAAAAAATTAGAGTGTTCAAAGCAGGCCCTGAATAATG

GTGCATGGAATAATGGAATAGGACCTCGGATTTTGTTGGTTTTCGGAACTGGAGGTAATGATTAAG

AGGGACGGCGGGGGCATTCGTATTGCGGTGTTAGAGGTGAAATTCTTGGATCGCCGCAAGACGAAC

TACTGCGAAAGCGTTTGCCAAGCACGTTTTCATTAGTCAAGAACGAAAGTCAGAGGTTCGAAGACG

ATCAGATACCGTCGTAGTTCTGACCATAAACGATGCCAGCTAGGGATTCGTCGGCGTTGTATGACT

CGACGAGCACCTTCCGGGAAACCAAAGCTTTTGGGTTCCGGGGGAAGTATGGTTGCAAAGCTGAAA

CTTAAAGGAATTGACGGAAGGGCACCACCAGGAGTGGAGCCTGCGGCTTAATTTGACTCAACACGG

GGAAACTCACCCGGCCCGGACACTGTAAGGATTGACAGATTGAGAGCTCTTTCTTGATTCGGTGGG

TGGTGGTGCATGGCCGTTCTTAGTTGGTGGAGCGATTTGTCTGGTTAATTCCGATAACGAACGAGA

CTCTAGCCTATTAAATAGTTCGCCGATTCGTCGGCGCAACTTCTTAGAGGGACAAGTGGCGTTTAG

CCACGCGAGATTGAGCAATAACAGGTCTGTGATGCCCTTAGATGTTCGGGGCCGCACGCGCGCTAC

ACTGAAGGAATCAGCGTGTGTTCTCCTGGCCTGAAAAGGTCGGGCAACCCGTTGAACCTCCTTCGT

GCTAGGGATTGGGGCTTGCAATTCTTCCCCATGAACGAGGAATTCCCAGTAAGCGCGAGTCATCAG

CTCGCGTTGATTACGTCCCTGCCCTTTGTACACACCGCCCGTCGCTACTACCGATTGGATGGTTTA

GTGAGACCCTCGGATTGAACTTGACGGTGGGCAACTGCCGTCGAGGTG-CGAGAAGACGGTCAAAC

TTGACTATCTAGAGGAAGTAAAAGT???

??

??

??

??

??

??

??

??

??

????????????????????????????

>Malacoceros_fuliginosus

---------------------AATTAGAGGCTGGAATGAATGAATATACGAGAGTTAAGCTGTTTCA

CTTCTAATAAAAACTAATCTTTAGGTGAAGAAGCCTAAATCATATAAAAAGACAAAAAGACCCTAT

TGAGCTTTACTAAAATAAAATTTAATTTTGCTAGTTTTGTTGGGGCGACAAAAGAAGACTAAATCT

TCTTTATA-TAAAAAATGATCCGAATCGATAACAAAAACAGCTACCATAGGGATAACAGGCTAATC

TTCTTTGAGAGCCCAAATTGACAAGAAGGATTGGCACCTCGATGTTGGCTTAGGATACCTGTTAGA

TGCAGAAGTAAGTTGGTTTGTTCAACCTTTAATATCCT---------TCTCAAAGATTAAGCCATG

CATGTCTAAGTACAAGCTCCACAAGGTGAAACCGCGAATGGCTCATTAAATCAGTTATGGTTCCTT

AGATCGTCTTATCCTACTTGGATAACTGTAGTAATTCTAGAGCTAATACATGCAATCAAGCTCCGA
CCTCGGGGGAAGGGCGCTTTTATTAGACCAAAACCAATGGGGGCGACTCGTTCTTGGTGACTCTGG
ATAACCTGCAGCTGATCGCACGGCCGCGCCTGCGACGTATCTTTCAAATGTCTGCCCTATCAGGTC
TCGACGGTTGGTGATATGCCAACCGTGCTTTTGACGGGTAGCGGGGAATCAGGGTTCGATTCCGGA
GAGGGAGCATGAGAAACGGCTACCACATCCAAGGAAGGCAGCAGGCGCGCAAATTACCCACTCCTG
GCTCGGGGAGGTAGTGACGAAAAATAACAATACGGGACTCTATTGAGGCCTCGTAATTGGAATGAG
TACACTTTAAATCCTTTAACAAGGATCCATTGGAGGGCAAGTCTGGTGCCAGCAGCCGCGGTAATT
CCAGCTCCAATAGCGTATATTAAAGTTGTTGCAGTTAAAAAGCTCGTAGTTGGATCATAGGGTCTG
GCTGGCGGTCCACGTCGCGAGGCGCTATCGCCTCCAGGCCCACTCGTCGGTCTCCATCGGTGCTCT
TCGCTGGGCCGGCCGTCACGTTTACTTTGAAAAAATTAGGGTGTTCAAAGCAGGCCCTGTATATCT
GCGCATGGAATAATTGAATAGGACCTCGGGTTTTGTTGGTTTCTGGAGTTCGAGGTAATGATCAAT
AGGGACTGCGGGGGCATCTGTATTGCAGCGGGAGAGGTGAAATTCATTGATCGTTGCAAGACAAAC
AAGTGCGAAGGCATTTGCCAAGAATGTCTTCATTAATCAAGAGCGAAAGTCAGAGGTTCGAAGACG
ATCAGATACCGTCGTAGTTCTGACCATAAATGATGCCAGCTATGGCTTTGCCGGCGTAGTTTGACT
CGGCGAGGCCCCCCCCGGAAACATAAGCTTATGGGTTCCGGGGGAAGTATGGTTGCAAAACTGAAA
CTTAAAGGAATTGACGGAAGGGCACCACCAGGAGTGGAGCCTGCGGCTTAATTTGACTCAACACGG
GGAAACTCACCCGGCCCGGACACCGTTAGGATTGACAGATTGAGAGCTCTTTCTTGATTCGGTGGG
TGGTGGTGCATGGCCGTTCTTAGTTGGTGGAGCGATTTGTCTGGTTAATTCCGATAACGAACGAGA
CTCTAGCCTATTAACTAGTTCGTCGATGCGTCGACGCAACTTCTTAGAGGGACTGGTGGCGTTTAG
CCACACGAGAATGAGCAATAACAGGTCTGTGATGCCCTTAGATGTCCGGGGCCGCACGCGCGCTAC
ACTGAAGGACTCAGCGAGTA---TCCTGGCCAGAAATGGTCGGGCAACCCATTGAACCTCCTTCGT
GCTAGGGATTGGGGTTTGCAATTCTCCCCCATGAACGAGGAATTCCCAGTAAGCGCGAGTCATCAG
CTCGCGTTGATTACGTCCCTGCCCTTTGTACACACCGCCCGTCGCTACTACCGATTGGATGGCTTA
GTGAGACCCTTGGACTGGTCTCGAGGGCTGGAAACAGCCGCCCTGCTGCCGGGAAGAAGGTCAAAC
TTGGCCATCTAGAGGAAGTAAAAGT???
??
??
??
??
??
??
??
??
??
????????????????????????????

>Malacoceros_sp_V040
??
??
??
??
??
??
??????????????????????????????????????----------------------------
----

--GCGAGGCCCCCCCCGGAAACATAAGCTTATGGGTTCCGGGGGAAGTATGGTTGCAAAACTGAAA
CTTAAAGGAATTGACGGAAGGGCACCACCAGGAGTGGAGCCTGCGGCTTAATTTGACTCAACACGG
GGAAACTCACCCGGCCCGGACACCGTTAGGATTGACAGATTGAGAGCTCTTTCTTGATTCGGTGGG
TGGTGGTGCATGGCCGTTYTTAGTTGGTGGAGCGATTTGTCTGGTTAATTCCGATAACGAACGAGA
CTCTAGCCTATTAACTAGTTCGTCGATGCGTCGACGCAACTTCTTAGAGGGACTGGTGGCGTTTAG
CCACACGAGAATGAGCAATAACAGGTCTGTGATGCCCTTAGATGTCCGGGGCCGCACGCGCGCTAC
ACTGAAGGACTCAGCGAGTA---TCCTGGCCAGAAATGGTCGGGCAACCCATTGAACCTCCTTCGT
GCTAGGGATTGGGGTTTGCAATTCTCCCCCATGAACGAGGAATTCCCAGTAAGCGCGAGTCATCAG
CTCGCGTTGATTACGTCCCTGCCCTTTGTACACACCGCCCGTCGCTACTACCGATTGGATGGCTTA
GTGAGACCCTTGGACTGGTCTCGAGGGCTGGAAACAGCCGCCCTGCTGCC----------------
----
---GACTGTAGAGGGAACTGTG
GTGTTAGGGAGAGCCTCTGGGGCGTTGGCGGCGTCCAAGTCCTCCTGATCGGGGTTGCCCATAGCG

GGTGTCAGGCCTTTACGATGGCTGCCGTCGTCCCTCTGTGCCTCCTAGGAGTCGGGTTGTTTGAGA
ATGCAGCCCAAAGTGGGAGGTAAATTCCTTCTAAGGCTAAATACCAGCACGAGTCCGATAGCAAAC
AAGTACCGTGAGGGAAAGTTGAAAAGAACTTTGAAGAGAGAGTTCAATAGTACGTGAAACCATGTA
GAAGTAAACGGATGGGACCGCAGTCGAACCTCGGGATTCAGCTCTCGCTT-GCTCGGTGAGGGACC
TCTCGGGGAGTGCACTTTCCGTGGTGAGAGCGCCAGGACCGTTTTCTGGGCCGCCAAGCCCCGGGG
GAAGGTAGCTCGAGTGTTATAGCCCCTGGAGGTGTTGGCCGGGGGGACCGCGGACTACCCGCCCGG
CCCTGCCGTACTGTGGAGGACTGCTTGCAGTGCTCTGCGACCGCGTTGTTCGGGTGTCCCAGGGTT
ACTGGCGTAGTCGGCAACCCATCCGACC

>Marenzelleria_arctia

-GCATAATAATTTGCCTCTTAATTAGAGGCTGGAATGAATGAATATACGAGAGTCTAGCTGTTTCA
TTTTTACTAAAAATTATCCTTTAAGTGAAGAGACTTAGATAGCATAAAAAGACAAAAAGACCCTAT
TGAGCTTTATTTTACAAAATCTTTTCTCTAAAAATTTTGTTGGGGCGACAAGAGAAGACTAAATCT
TCTCTATTTTACAAAATGATCCGTAACGATAACAGAAACAGCTACCATAGGGATAACAGGCTAATC
TTCTTTGAGAGCCCAAATTGACAAGAAGGA------------------------------------
---TAAGCCATG
CATGTCTAAGTATAAGCTCGACAAGGTGAAACCGCGAATGGCTCATTAAATCAGTTATGGTTCCTT
AGATCGTACTATCCTACTTGGATACCTGTAGTAATTCTAGAGCTAATACATGCACGGAAGCTCCGA
CCCTCGGGGAAGAGCGCTTTTATTAGACCAAAACCAACGGGGGCAACCAATCTTTGGTGACTCTGG
ATAACTTCGAGCGGATCGCACGGCCGCGCCGGCGACGTATCTTTCAAATGTCTGCCCTATCAAGTT
TCGACGGTCGGCGACATGCCTACCGTGCTTTTAACGGGTAGCGGGGAATCAGGGTTCGATTCCGGA
GAGGGAGCATGAGAAACGGCTACCACATCCAAGGAAGGCAGCAGGCGCGCAAATTACCCACTCCTG
GCTCGGGGAGGTAGTGACGAAAAATAACAATACGGGACTCTTACGAGGCCCCGTAATTGGAATGAG
TACACTTTAAATCCTTTAACGAGGATCCATTGGAGGGCAAGTCTGGTGCCAGCAGCCGCGGTAATT
CCAGCTCCAATAGCGTATATTAAAGTTGCTGCAGTTAAAAAGCTCGTAGTTGGATCTCGGGCGCGG
GCCGGCGGTCCCCCTCGCGGGGGTCAACTGCCTCCCGCGCTCCTGGTCGGTGGCTCCGGATGCCCT
TCGCTGGCGCAGCCGGCTGGTTTACTTTGAAAAAATTAGAGTGTTCAAAGCAGGCCCTGTATACCG
TCGCATGGAATAATGGAATAGGACCTCGGATTTTGTTGGTTGTTGGAG-TCGAGGTAATGATCAAT
AGGGACGGCGGGGGCATTCGTATTGCGCCGTTAGAGGTGAAATTCTTGGATCGGCGCAAGACGAAC
TAAAGCGAAAGCAATTGCCAAGAGCGTCTTCTTTAATCAAGAGCGACAGTCAGAGGTTCGAAGACG
ATCAGATACCGTCGTAGTTCTGACCATAAACGATGCCAGCTGGCGATGTGCCGGAGTTGTATGACT
CTGCACGCAGTCTCCGGGAAACCAAAGCTTTTGGGTTCCGGGGGAAGTATGGTTGCAAAGCTGAAA
CTTAAAGGAATTGACGGAAGGGCACCACCAGGAGTGGAGCCTGCGGCTTAATTTGACTCAACACGG
GGAAACTCACCCGGCCCGGACACCGTAAGGATTGACAGATTGAGAGCTCTTTCTTGATTCGGTGGG
TGGTGGTGCATGGCCGTTCTTAGTTGGTGGAGCGATTTGTCTGGTTAATTCCGATAACGAACGAGA

CTCTAGCCTATTAAATAGTTCGCCGGTTTGCCGGCGTAACTTCTTAGAGGGACAAGTGGCGTTCAG
CCACGCGAGAGTGAGCAATAACAGGTCTGTGATGCCCTTAGATGTTCGGGGCCGCACGCGCGCTAC
ACTGAAGGACTCAGCACGTCTTTTCCCGGCCAGAAATGGTCGGGCAACCCGTTGAACCTCCTTCGT
GCTAGGGATTGGGGCTTGCAATTCTTCCCCATGAACGAGGAATTCCCAGTAAGCGCGAGTCATCAG
CTCGCGTTGATTACGTCCCTGCCCTTTGTACACACCGCCCGTCGCTACTACCGATTGGATGGTTTA
GTGAGACCCTCGGACTGGTCCCGGCGGCGGGCGACCGCTGCCGAGGTGCTGGGAAGACGGTCGAAC
TTGACTATCTAGAGGAAGTAAAAGTTCACTAAGCGGAGGAAAAGAAACTAACCAGGATTCCCCTAG
TAACGGCGAGTGAAGAGGGAAAAGCCCAGCACCGAATCCCCTGCATAGACTGTAGCGGGAACTGTG
GTGTTCGGGAGAGCCTCTGGAGCGGGAC-GGCGTCCGAGTCCTTCTGATCGGGGTTACCCAGAGCG
GGTGTTAGGCCTCTACGGCGCCGCTTTCCGTTCCTCTGTGCCTCCCAGGAGTCGGGTTGTTTGCGA
ATGCAGCCCAAAGTGGGTGGTAAACTCCATCTAAGGCTAAATACTGGCGCGAGTCCGATAGCGGAC
AAGTACCGTGAGGGAAAGTT--

>Marenzelleria_viridis

--------------------AATTAGAGGCTGGAATGAATGAATATACGAGAGTTTAGCTGTTTCA
TTTTTACTAAAAATTATCCTTTAAGTGAAGAGACTTAAATAAAATAAAAAGACAAAAAGACCCTAT
TGAGCTTTATTTCATAAAACTTTACCTACAGAAATTTTGTTGGGGCAACAAGAGAAGACTAAATCT
TCTCTATACTACAAAATGATCCGAAACGATAACAGAACCAGCTACCATAGGGATAACAGGCTAATC
TTCTTTGAGAGCCCAAATTGACAAGAAGGATTGGCACCTCGATGTTGGCTTAGGATACCTGATAGA
TGCAGAAGTAAGTGGGTTTGTTCAACCTTTAATATCCTATATGCTTGTCTCAAAGACTAAGCCATG
CATGTCTAAGTATAAGCTCGACAAGGTGAAACCGCGAATGGCTCATTAAATCAGTTATGGTTCCTT
AGATCGTACTATCCTACTTGGATACCTGTAGTAATTCTAGAGCTAATACATGCACGGAAGCTCCGA
CCCCCGGGGAAGAGCGCTTTTATTAGACCAAAACCAACGGGGGCGACTAAACTTTGGTGACTCTGG
ATAACTTCGAGCGGATCGCACGGCCGCGCCGGCGACGTATCTTTCAAATGTCTGCCCTATCAAGTT
TCGACGGTCGGCGACATGCCTACCGTGCTTATTACGGGTAGCGGGGAATCAGGGTTCGATTCCGGA
GAGGGAGCATGAGAAACGGCTACCACATCCAAGGAAGGCAGCAGGCGCGCAAATTACCCACTCCTG
GCTCGGGGAGGTAGTGACGAAAAATAACAATACGGGACTCTTACGAGGCCCCGTAATTGGAATGAG
TACACTTTAAATCCTTTAACGAGGATCCATTGGAGGGCAAGTCTGGTGCCAGCAGCCGCGGTAATT
CCAGCTCCAATAGCGTATATTAAAGTTGCTGCAGTTAAAAAGCTCGTAGTTGGATCTCGGGCGCGG
GCCGGCGGTCCCCCTCGCGGGGGTTCACTGCCTCCCGCGCCCCTGGCCGGTACGCCCGGGTGCCCT

TCGCTGGTGCAGCCGGCTGGTTTACTTTGAAAAAATTAGAGTGTTCAAAGCAGGCCCTGCATACCG
TCGCATGGAATAATGGAATAGGACCTCGGATTTTGTTGGTTGTTGGAG-TCGAGGTAATGATCAAT
AGGGACGGCGGGGGCATTCGTATTGCGCCGTTAGAGGTGAAATTCTTGGATCGGCGCAAGACGAAC
TAGAGCGAAAGCAATTGCCAAGAGCGTCTTCTTTAATCAAGAGCGACAGTCAGAGGTTCGAAGACG
ATCAGATACCGTCGTAGTTCTGACCATAAACGATGCCAGCTGGCGATGTGCCGGAGTTGTATGACT
CTGCACGCAGCCCCCGGGAAACCAAAGCTTTTGGGTTCCGGGGGAAGTATGGTTGCAAAGCTGAAA
CTTAAAGGAATTGACGGAAGGGCACCACCAGGAGTGGAGCCTGCGGCTTAATTTGACTCAACACGG
GGAAACTCACCCGGCCCGGACACCGTAAGGATTGACAGATTGAGAGCTCTTTCTTGATTCGGTGGG
TGGTGGTGCATGGCCGTTCTTAGTTGGTGGARCGATTTGTCTGGTTAATTCCGATAACGAACGAGA
CTCTAGCCTATTAAATAGTTCGCCGGTTCGCCGGCGTAACTTCTTAGAGGGACAAGTGGCGTTCAG
CCACGCGAGAGTGAGCAATAACAGGTCTGTGATGCCCTTAGATGTTCGGGGCCGCACGCGCGCTAC
ACTGAAGGACTCAGCACGTCTTCTCCCGGCCAGAAATGGTCGGGCAACCCGCTGAACCTCCTTCGT
GCTAGGGATTGGGGCTTGCAATTCTTCCCCATGAACGAGGAATTCCCAGTAAGCGCGAGTCATCAG
CTCGCGTTGATTACGTCCCTGCCCTTTGTACACACCGCCCGTCGCTACTACCGATTGGATGGTTTA
GTGAGACCCTCGGACCGGTCCCGGCGGCGGGCGACCGCTGCCGAGGTGCTGGGAAGACGGTCGAAC
TTGACTATCTAGAGGAAGTAAAAGTTCACTAAGCGGAGGAAAAGAAACTAACCAGGATTCCCCTAG
TAACGGCGAGTGAAGAGGGAAAAGCCCAGCACCGAATCCCCGGCGTCGACCGTCGAGGGAACTGTG
GTGTTCGGGAGAGCCTCTGGAGCGGGAC-GGCGTCCGAGTCCTTCTGATAGGGGTTACCCAGAGCG
GGTGTTAGGCCTCTACGGCGCCGCTTTCCGTTCCTCTGCGCCTCCCAGGAGTCGGGTTGTTTGCGA
ATGCAGCCCAAAGTGGGTGGTAAACTCCATCTAAGGCTAAATACTGGCGCGAGTCCGATAGCGGAC
AAGTACCGTGAGGGAAAGTTGAAAAGAACTTTGAAGAGAGAGTTCAAGAGTACGTGAAACCGTATA
GAGGCAAACGGATGGGACCGCAGTCGACCCTCGGAATTCAGCTTTCGCCCGGCTAGCTAGGGACCT
TCACCGGGAGTGCACTTTTCGGGGGGAGAGCGCCGCGACCGGTTCCCGGGCCGTCAGCTCCGGCGG
GAAGGTAGCTCGAGTGTTACAGCCCCCGGCAG--TTGGCCGGGGGGACCGAGGAATACCCGCCAAA
GACTGGTCGACTGGCGAGGACTGCTCGCAGTGCTCGCCGACTGCGCCGCTCGAGACCACTCGGGTC
TGCGGCGAAGTCGGTTTCCCATCCGACC

>Microspio_granulata
--AGGT
AGCATAATCATTTGTCTTTTAATTGGAGACTAGTATGAACGAGAATATGCCTCACCTACTTTTCTA
ATTTAAACTGAAATTAATCTATAGGTGAATAAGCCTATATTATAAATAAAGACAGAAAGACCCTAT
AGAGCTTTATCCTCATAATCATATGAATAAAGGATTTTATTGGGGCAATATAAGATTACATAAACA
TCTTAATATACAG-----------------TCACTAGATAGCTACCTTAGGGATAACAGACTAATA
TATTAATAAAGCTCTTATTTGTTAATTTGCTTAGTACCTCGATGTTGGTTTAGGGTTTCCAATTGG
TGTAGCCGCGGGATGGGTTGTTCACCCATTAA----------------------------------
---------GTATAAGCTCTACACGGCGAAACCGCGAATGGCTCATTAAATCAGTTATGGTTCCTT

AGATCGTACCATCCTACTTGGATAACTGTAGTAATTCTAGAGCTAATACATGCCAAGAAGCTCCGA
CCCTCGGGGAAGAGCGCTTTTATTAGACCAAAACCAGAGCTCGCGGCCAACCTTTGGTGACTCTGG
ATAACTTG-AGCAGATCGCACGGCCGAGCCGGCGACGTATCTTTCAAATGTCTGCCCTATCAACTG
TCGACGGTTGGTGACATGCCAACCGTGGTTATAACGGGTAGCGGGGAATCAGGGTTCGATTCCGGA
GAGGGAGCATGAGAAACGGCTACCACATCCAAGGAAGGCAGCAGGCGCGCAAATTACCCACTCCTA
GCTCGGGGAGGTAGTGACGAAAAATAACAATACGGGACTCTTACGAGGCCCCGTAA----------
----

-------------------------???
??
??
??
??
??
??
??
??
??
????????????????????????????

>Paraprionospio_coora

CGCCTCTAGATTAGAGGTCCATCCTGCCCAGTGTTAACGGCCGCGGTATCCTGACCGTGCTAAGGT

AGCATAATCGTTTGCCTTTTAATTGGAGGCTAGTATGAACGGACACACGAGAGTATATCTATATCG

TTAAAGATGAAAACTATCTTTTAGGTGAAGAGACCTGAATTTTATAAAAAGACGAAAAGACCCTAT

GGAGCTTTACTTAGCCTAAATATAACAAGAAAAGTTTAGTTGGGGCGACTAAGGAACAATATTAGC

TTCCTAATAATAATATATATCCGCAGCGA-CATGAAATCAGCTACCCTAGGGATAACAGGCTAATC

CTCTTCGAAAGTTCAAATTGACAAGAGGGGTTGGCACCTCGATGTTGGCTTAAGGCTTCTTCTTGG

CGCAGCAGCTAGTGGGTTTGTTCAACCCTTAAAGCCTTATATGCTTGTCTCAAAGACTAAGCCATG

CATGTCTAAGTACAAGCTTAACACGGTGAAACCGCGAATGGCTCATTAAATCAGTTATGGTTCCTT

AGATCGTACAATCCTACTTGGATAACTGTAGTAATTCTAGAGCTAATACATGCAAGCAAGCTCCGA

CCTTCGGGAAAGAGCGCTTTTATTAGACCAAAACCAAT--GGGCAACTTCTTGTTGGTGACTCTGG

ATAACTTTGAGCTGATCGCACGGCCGAGCCGGCGACGTATCTTTCAAATGTCTGCCCTATCAACTT

TCGACGGTACGTGATATGCCTACCGTGGTTGTAACGGGTAACGGGGAATCAGGGTTCGATTCCGGA

GAGGGAGCATGAGAAACGGCTACCACATCCAAGGAAGGCAGCAGGCGCGCAAATTACCCACTCCTG

GCACGGGGAGGTAGTGACGAAAAATAACAATACGGGACTCTTTCGAGGCCCCGTAATTGGAATGAG

TACACTTTAAATCCTTTAACGAGGATCTATTGGAGGGCAAGTCTGGTGCCAGCAGCCGCGGTAATT

CCAGCTCCAATAGCGTATATTAAAGTTGTTGCAGTTAAAAAGCTCGTAGTTGGATCTCGGGAGCAG

GCCGGCGGTCCGCCTCGCGGCGG-CTACTGCCTGTCCTGCCCCCTGCCGGTTTCCCTCGGTGCTCT

TCATTGGGGTGGCCGGAACGTTTACTTTGAAAAAATTAGAGTGTTCAAAGCAGGCCCTGAATAATG

GTGCATGGAATAATGGAATAGGACCTCGGATTTTGTTGGTTTTCGGAGCTCGAGGTAATGATTAAG

AGGGACGGCGGGGGCATTCGTATTGCACTGTTAGAGGTGAAATTCTTGGATCGGTGCAAGACGCTC

GACTGCGAAAGCATTTGCCAAGAATGTTTTCATTAATCAAGAACGAAAGTCAGAGGTTCGAAGACG

ATCAGATACCGTCGTAGTTCTGACCATAAACGATGCCAGCTAGGGATTCGTCGGAGTTGTATGACT

CGACGAGCACCTTCCGGGAAACCAAAGCTTTTGGGTTCCGGGGGAAGTATGGTTGCAAAGCTGAAA

CTTAAAGGAATTGACGGAAGGGCACCACCAGGAGTGGAGCCTGCGGCTTAATTTGACTCAACACGG

GGAAACTCACCCGGCCCGGACACCGTAAGGATTGACAGATTGAGAGCTCTTTCTTGATTCGGTGGG

TGGTGGTGCATGGCCGTTCTTAGTTGGTGGAGCGATTTGTCTGGTTAATTCCGATAACGAACGAGA

CTCTAGCCTATTAAATAGTTCGCCGATTCGTCGGCGCAACTTCTTAGAGGGACAAGTGGCGTTTAG

CCACGCGAGATTGAGCAATAACAGGTCTGTGATGCCCTTAGATGTTCGGGGCCGCACGCGCGCTAC

ACTGAAGGAATCAGCGTGTGTTCTCCTGGCCAGAAATGGTCGGGTAACCCGTTGAACCTCCTTCGT

GCTAGGGATTGGGGTTTGCAATTATCCCCCATGAACGAGGAATTCCCAGTAAGCGCGAGTCATCAG

CTCGCGTTGATTACGTCCCTGCCCTTTGTACACACCGCCCGTCGCTACTACCGATTGGATGGTTTA

GTGAGACCCTCGGATTGGTCCCGGAGGCGGGTCACCGCCACCGTGGCGCCGAAAAGACGGTCAAAC

TTGACTATCTAGAGGAAGTAAAAGT???

??

??

??
??
??
??
??
??
??
????????????????????????????

>Paraprionospio_patiens
CGCCTCTAGATTAGAGGTTCATCCTGCCCAGTGTCAACGGCCGCGGTATCCTGACCGTGCTAAGGT
AGCATAATCGTTTGCCTTTTAATTGAAGGCTGGTATGAATGGACAAACGAAAGTACATCTGTATCA
TTAAAGATGAAAATTATCTTTTAGGTGAAGAGCCCTGAATTTTTTAAAAAGACGAAAAGACCCTAT
GGAGCTTTACTTATCTTATTCGTAGTTAATAAAGTTTAGTTGGGGCGACTAAGGAACAATGAAAGC
TTCCTATAAATTTAATTTATCCGTTACGA-TATGGAACCAGCTACCCTAGGGATAACAGGCTAATC
CTCTTCGAGAGTTCAAATTTACAAGAGGGGTTGGCACCTCGATGTTGGCTTAAGGCTTCTTTTTGG
CGCAGCAGCAAGTGGGTTTGTTCAACCTTTAAAGCCTT---------------------------G
CATGTCTAAGTACAAGCTTAACACGGTGAAACCGCGAATGGCTCATTAAATCAGTTATGGTTCCTT
AGATCGTACAATCCTACTTGGATAACTGTAGTAATTCTAGAGCTAATACATGCAAGCAAGCTCCGA
CCTTCGGGAAAGAGCGCTTTTATTAGACCAAAACCAAT--GGGCAACTTCTAGTTGGTGACTCTGG
ATAACTTTGAGCTGATCGCACGGCCGAGCCGGCGACGTATCTTTCAAATGTCTGCCCTATCAACTT
TCGACGGTACGTGACATGCCTACCGTGGTTGTAACGGGTAACGGGGAATCAGGGTTCGATTCCGGA
GAGGGAGCATGAGAAACGGCTACCACATCCAAGGAAGGCAGCAGGCGCGCAAATTACCCACTCCTG
GCACGGGGAGGTAGTGACGAAAAATAACAATACGGGACTCTTTCGAGGCCCCGTAATTGGAATGAG
TACACTTTAAATCCTTTAACGAGGATCTATTGGAGGGCAAGTCTGGTGCCAGCAGCCGCGG-----
-------CAATAGCGTATATTAAAGTTGTTGCAGTTAAAAAGCTCGTAGTTGGATCTCGGGAGCAG
GCCGGCGGTCCGCCTCGCGGCGG-CTACTGCCTGTCCTGCCCCCTGCCGGTTTCCCTCGGTGCTCT
TCGCTGGGGTGGCCGGAACGTTTACTTTGAAAAAATTAGAGTGTTCAAAGCAGGCCCTGAATAATG
GTGCATGGAATAATGGAATAGGACCTCGGATTTTGTTGGTTTTCGGAGCTCGAGGTAATGATTAAG
AGGGACGGCGGGGGCATTCGTATTGCACTGTTAGAGGTGAAATTCTTGGATCGGTGCAAGACGAAC
TACTGCGAAAGCATTTGCCAAGAATGTTTTCATTAATCAAGAACGAAAGTCAGAGGTTCGAAGACG
ATCAGATACCGTCGTAGTTCTGACCATAAACGATGCCAGCTAGGGATTCGCCGGAGTTGTATGACT
CGGCGAGCACCTTCCGGGAAACCAAAGCTTTTGGGTTCCGGGGGAAGTATGGTTGCAAAGCTGAAA
CTTAAAGGAATTGACGGAAGGGCACCACCAGGAGTGGAGCCTGCGGCTTAATTTGACTCAACACGG
GGAAACTCACCCGGCCCGGACACCGTAAGGATTGACAGATTGAGAGCTCTTTCTTGATTCGGTGGG
TGGTGGTGCATGGCCGTTCTTAGTTGGTGGAGCGATTTGTCTGGTTAATTCCGATAACGAACGAGA

CTCTAGCCTATTAAATAGTTCGCCGATTCGTCGGCGCAACTTCTTAGAGGGACAAGTGGCGTTTAG
CCACGCGAGATTGAGCAATAACAGGTCTGTGATGCCCTTAGATGTTCGGGGCCGCACGCGCGCTAC
ACTGAAGGAATCAGCGTGTGTTCTCCTGGCCAGAAATGGTCGGGTAACCCGTTGAACCTCCTTCGT
GCTAGGGATTGGGGTTTGCAATTATCCCCCATGAACGAGGAATTCCCAGTAAGCGCGAGTCATCAG
CTCGCGTTGATTACGTCCCTGCCCTTTGTACACACCGCCCGTCGCTACTACCGATTGGATGGTTTA
GTGAGACCCTCGGATTGGTCCCGGAGGTGGGCAACCGCCACCGTGGCGCCGAAAAGACGGTCAAAC
TTGACTATCTAGAGGAAGTAAAAGT???
??
??
??
??
??
??
??
??
??
????????????????????????????

>Poecilochaetus_serpens
CGCCTCCAGATTGGAGGTCCATCCTGCCCAATGTAAATGGCCGCGGTACCCTGACCGTGCAAAGGT
AGCATAATCATTTGCCTCCTAATTAGAGGCTAGTATGAATGGACACACGAGAGTCAAGCTGTTTCA
CTCTTAATAAAAATTAATCTTCAGGTGAAAAAGCCTGAATTAAATAAAAAGACAAAAAGACCCTAT
TGAGTTTTATTGGAACCAGAAAAAGCAGAACCAATTTAGTTGGGGCGACTGAGGAAGAATTAAATC
TTCCTTACAAATAAAATGATCCGATACGATAAAAGAAAGAACTACCATAGGGATAACAGGCTAATT
TTCTTTGAGAGTCCATATTGACAAGAAAGTTTGGCACCTCGATGTTGGCTTAGAATACCTATAA--
--------------------------------------ATATGCTTGTCTCAAAGACTAAGCCATG
CATGTCTAAGTACAAGCTTAACAAGGTGAAACCGCGAATGGCTCATTAAATCAGTTATGGTTCCTT
AGATCGTTCCATCCTACTTGGATAACTGTAGTAATTCTAGAGCTAATACATGCCAGCAAGCTCCGA
CCTTACGGGAAGAGCGCTTTTATTAGACCAAAACCAAC--GGGCAACCAAAGCTTGGTGACTCTGG
ATAACTTTGTGCCGATCGCACGGCCGCGCCGGCGACGTATCTTTCAAATGTCTGCCCTATCAACTT
TCGATGGTACGTGCTATGCCTACCATGGTTGTAACGGGTAACGGGGAATCAGGGTTCGATTCCGGA
GAGGGAGCATGAGAAACGGCTACCACATCCAAGGAAGGCAGCAGGCGCGCAAATTACCCACTCCTG
GCACGGGGAGGTAGTGACGAAAAATAACAATACGGGACTCTTTCGAGGCCCCGTAATTGGAATGAG
TACACTTTAAATCCTTTAACGAGGATCTATTGGAGGGCAAGTCTGGTGCCAGCAGCCGCGGTAATT
CCAGCTCCAATAGCGTATATCAAAGTTGTTGCAGTTAAAAAGCTCGTAGTTGGATCTCGGGAGCAG
GCGTGCGGTCCACCTCGCGGTGG-TCACTGCGTCCTGCCCTATTTGCCGGACTCCCCCGGTGCCCT

TCGCTGGGGTGGCCGGAACGTTTACTTTGAAAAAATTAGAGTGTTCAAAGCAGGCCCTGAATAATG
GTGCATGGAATAATGGAATAGGACCTCGGATTTTGTTGGTTTCCGGAGCTCGAGGTAATGATTAAG
AGGGACGGCGGGGGCATTTGTATTGCGGCGTTAGAGGTGAAATTCTTGGATCGTCGC-AGACAAAC
CACAGCGAAAGCATTTGCCAAGAATGTTTTCATTAATCAAGAACGAAAGTCAGAGGTTCGAAGACG
ATCAGATACCGTCGTAGTTCTGACCATAAACGATGCCAGCTAGGGATCCGCCGGCGTTGAATGACC
CGGCGAGCACCTTCCGGGAAACC-AAGCTTTTGGGTTCCGGGGGAAGTATGGTTGCAAAGCTGAAA
CTTAAAGGAATTGACGGAAGGGCACCACCAGGAGTGGAGCCTGCGGCTTAATTTGACTCAACACGG
GAAAACTCACCCGGCCCGGACACTGTAAGGATTGACAGATTGAGAGCTCTTTCTTGATTCGGTGGG
TGGTGGTGCATGGCCGTTCTTAGTTGGTGGAGCGATTTGTCTGGTTAATTCCGATAACGAACGAGA
CTCTAGCCTGCTAAATAGTCCGCCGGTTCGCCGGTGCGACTTCTTAGAGGGACAAGTGGCGTTTAG
CCACGCGAGATTGAGCAATAACAGGTCTGTGATGCCCTTAGATGTTCGGGGCCGCACGCGCGCTAC
ACTGAAAGAATCAACGTGTGTCCCCCTGGCCTGAAAAGGTCGGGCAACCCGCTGAACCTCTTTCGT
GCTAGGGATTGGGGCTTGCAATTGTTCCCCATGAACGAGGAATTCCCAGTAAGCGCGAGTCATCAG
CTCGCGTTGATTACGTCCCTGCCCTTTGTACACACCGCCCGTCGCTACTACCGATTGGATGGTTTA
GTGAGACCCTCGGATTGGTCCCGACGGTGGGCAACCGCCGTCGTGGCGCCGAAAAGACGGTCAAAC
TTGACTATCTAGAGGAAGTAAAAGT???
??
??
??
??
??
??
??
??
??
????????????????????????????

>Poecilochaetus_sp_VR_2006

-GCATAATCATTTGCCTCTTAATTAGAGGCTTGTATGAATGGACACACGAGAGTCATACTGTTTCA
CTTTTAATAAAAATTAATCTTTAGGTGAAAAAGCCTAAATCAAATAAAAAGACAAAAAGACCCTAT
TGAGTTTTATTGAAACCAATGAAAATATAATCAATTTAGTTGGGGCGACTAAGGAAGAATTAAATC
TTCCTCTAACATAAACTGATCCACCGTGATAAAAGATCTAACTACCATAGGGATAACAGGCTAATC
TTCTTTGAGAGCCCTTATTGACAAGAAGGA------------------------------------
----
-------AAGTACAAGTTCAAAACGGTGAAACCGCGAATGGCTCATTAAATCAGTTATGGTTCCTT

AGATCGTCCAATCCTACTTGGATAACTGTAGTAATTCTAGAGCTAATACATGCAAGCAAGCTCCGA
CCTTCGGGGAAGAGCGCTTTTATTAGACCAAAACCAAC--GGGCAACCAAACCTTGGTGACTCTGG
ATAACTTTGAGCAGATCGCACGGCCGCGCCGGCGACGTATCTTTCAAATGTCTGCCCTATCAACTT
TCGATGGTACGTGCTATGCCTACCATGGTTGTAACGGGTAACGGGGAATCAGGGTTCGATTCCGGA
GAGGGAGCATGAGAAACGGCTACCACATCCAAGGAAGGCAGCAGGCGCGCAAATTACCCACTCCTG
GCACGGGGAGGTAGTGACGAAAAATAACAATACGGGACTCTTTCGAGGCCCCGTAATTGGAATGAG
TACACTTTAAATCCTTTAACGAGGATCTATTGGAGGGCAAGTCTGGTGCCAGCAGCCGCGGTAATT
CCAGCTCCAATAGCGTATATCAAAGTTGTTGCAGTTAAAAAGCTCGTAGTTGGATCTCGGGAGCAG
GCGTGCGGTCCGCCTCGCGGCGGTTCACTGCGTCCTGCCCTATCTGCCGGACTCCCCCGGTGCCCT
TCGCTGGGGTGGCCGGGACGTTTACTTTGAAAAAATTAGAGTGTTCAAAGCAGGCCCTGAATAATG
GTGCATGGAATAATGGAATAGGACCTCGGATTTTGTTGGTTTCCGGAGCTCGAGGTAATGATTAAG
AGGGACGGCGGGGGCATTTGTATTGCGGCGTTAGAGGTGAAATTCTTGGATCGTCGCAAGACAGAC
CACAGCGAAAGCATTTGCCAAGAATGTTTTCATTAATCAAGAACGAAAGTCAGAGGTTCGAAGACG
ATCAGATACCGTCGTAGTTCTGACCATAAACGATGCCAGCTAGGGATCCGTCGGCGTTGAATGACT
CGGCGAGCACTTTCCGGGAAACCAAAGCTTTTGGGTTCCGGGGGAAGTATGGTTGCAAAGCTGAAA
CTTAAAGGAATTGACGGAAGGGCACCACCAGGAGTGGAGCCTGCGGCTTAATTTGACTCAACACGG
GAAAACTCACCCGGCCCGGACACTGTAAGGATTGACAGATTGAGAGCTCTTTCTTGATTCGGTGGG
TGGTGGTGCATGGCCGTTCTTAGTTGGTGGAGCGATTTGTCTGGTTAATTCCGATAACGAACGAGA
CTCTAGCCTGCTAAATAGTCCGCCGGTGTGCCGGCGCGACTTCTTAGAGGGACAAGTGGCGTTTAG
CCACGCGAGATTGAGCAATAACAGGTCTGTGATGCCCTTAGATGTTCGGGGCCGCACGCGCGCTAC
ACTGAAAGAATCAACGTGTGTTCCCCTGGCCTGAAAAGGTCGGGCAACCCGCTGAACCTCTTTCGT
GCTAGGGATTGGGGCTTGCAATTGTTCCCCATGAACGAGGAATTCCCAGTAAGCGCGAGTCATCAG
CTCGCGTTGATTACGTCCCTGCCCTTTGTACACACCGCCCGTCGCTACTACCGATTGGATGGTTTA
GTGAGACCCTCGGATTGGTCCCGACGGTGGGCAACCGCCGTCGTGGCGCCGAAAAGACGGTCAAAC
T------------------------TCACTAAGCGGAGGAAAAGAAACTAACAAGGATTCCCCTAG
TAACGGCGAGTGAAGCGGGAAGAGCCCAGCACCGAATCCCCCGCGTCGACCGTGGCGGGAACTGTG
GTGTGTGGGAGAGCCTCTGGGCCGGCGGTGGCGTCCGAGTCCTCCTGATCGGGGACATCCATAGCG
GGTGTCAGGCCTCTACGGCCTCCGCCGCCGGCCCTCTGCGCCTCCTCGGAGTCGGGTTGTTTGTGA
ATGCAGCCCAAAGTGGGTGGTAAACTCCATCTAAGGCTAAATACTGGCACGAGACCGATAGCGGAC
AAGTACCGTGAGGGAAAGTT--

>Polydora_cornuta

CGCCTCTAGATTAGAGGTACATTCTGCCCTATGTAAATGGCCGCGGTAGCCTGACCGTGCTAAGGT

AGCATAATCATTTGCCGTTTAATTGATGGCTAGAATGAATGAATATACGAGAGCTTAACTGTCTCA

TCTTTAATATAAATTGACCTTCAAGTGAAGAGACTTGAATTACAAAAAAAGACGAAAAGACCCTAT

AGAGCTTTATTTTACCCTTTCTCACCTCAGAAAATTTAGTTGGGGCAACTAGAGAGAACTAAAACC

TCTCTGAAAA----TTTGATCCGACACGAAAAAAGAAAAAGCTACCTTAGGGATAACAGGCTAATC

CTCTTTTAGAGACCATATTGACAAGAGGGATTGGCACCTCGATGTTGGCTCAGGGTTACAGTTTGA

TGCAGCAGTAAGTAGGTTTGTTCACCCTTTAAACCCTTATATGCTTGTCTCAAAGACTAAGCCATG

CATGTCTAAGTATAAGCTCAACATGGTGAAACCGCGAATGGCTCATTAAATCAGTTATGGTTCCTT

AGATCTTAACATCCTACTTGGATAACTGTAGTAATTCTAGAGCTAATACATGCACCCGAGCTCCGA

CCCTCGGGAAAGAGCGCTTTTATTAGACCAAAACCAATTGCTCGTGCCAACCTTTGGTGACTCTGG

ATAACTTC-AGCTGATCGCATGGCCGCGCCGGCGACGTATCTTTCAAATGTCTGCCCTATCAAGTT

TCGATGGTTGGTGACTTGCCAACCATGCTCGTAACGGGTAGCGGGGAATCAGGGTTCGATTCCGGA

GAGGGAGCATGAGAAACGGCTACCACATCCAAGGAAGGCAGCAGGCGCGCAAATTACCCACTCCTA

GCTCGGGGAGGTAGTGACGAAAAATAACAATACGGGACTCTTACGAGGCCCCGTAATTGGAATGAG

TACACTTTAAATCCTTTAACGAGGATCTATTGGAGGGCAAGTCTGGTGCCAGCAGCCGCGGTAATT

CCAGCTCCAATAGCGTATATTAAAGTTGTTGCAGTTAAAAAGCTCGTAGTTGGATCTCGGGAGCGG

TCCGGCGGTCCTCCTCACGGAGGTTCACTGCCYGCCGCTCTCCTCACCGGCTTCCCCGGGTGCTCT

TCGCTGAGGCGGCCGGAATGTTTACTTTGAAAAAATTAGAGTGTTCAAAGCAGGCCCTGAATATTG

GCGCATGGAATAATGGAATAGGACCTCGGATTTTGTTGGTTTTCGGAAGTCGAGGTAATGATTAGT

AGGGACGACGGGGGCATTCGTATTGCGCGGTTAGAGGTGAAATTCTTGGATCCGCGCAAGACGAAC

TACAGCGAAGGCGTCTGCCAAGTGCGTCTTCATTAATCAAGAGCGACAGTCAGAGGTTCGAAGACG

ATCAGATACCGTCGTAGTTCTGACCATAAACGATGCCAGCTAGGGATGGGCGGACGTTGTTCGACT

CCGCCCGGACCTTCCGGGAAACCAAAGCTTTTGGGTTCCGGGGGAAGTATGGTTGCAAAGCTGAAA

CTTAAAGGAATTGACGGAAGGGCACCACCAGGAGTGGAGCCTGCGGCTTAATTTGACTCAACACGG

GGAAACTCACCCGGCCCGGACACCGTAAGGATTGACAGATTGAAAGCTCTTTCTTGATTCGGTGGG

TGGTGGTGCATGGCCGTTCTTAGTTGGTGGAGCGATTTGTCTGGTTAATTCCGATAACGAACGAGA

CTCTAGCCTGCTAAATAGTTCGTCGGTTTGCCGGCGCAACTTCTTAGAGGGACAAGTGGCGTTTAG

CCACGCGAGATTGAGCAATAACAGGTCTGTGATGCCCTTAGATGTTCGGGGCCGCACGCGCGCTAC

ACTGAAGGACTCAGCTGGT--TCTCCTGGCCTGAAAAGGTCGGGCAACCCGTTGAACCTCCTTCGT

GCTAGGGATTGGGGTTTGCAATTATTCCCCATGAACGAGGAATTCCCAGTAGGCGCGAGTCATCAG

CTCGCGTCGATTACGTCCCTGCCCTTTGTACACACCGCCCGTCGCTACTACCGATCGGATGGTTTA

GTGAGACCCTTGGACTGGTCCCGTCAGTGGGCAACCGCCGACGCGTTGCCGGGAAGAAGGTCGAAC

TT------------------------CACTAAGCGGAGGAAAAGAAACTAACCAGGATTCCCTTAG

TAACGGCGAGTGAAACGGGAAGAGCCCAGCACCGAATCCCCTGCCCTG--TGCAGCGGGAACTGTG

GTGTTTAGGAGAGCCTGTGGAGCGGCGG-AGCGTCCGAGTCCTTATGAAAGGGGACAGCCAGAGCG

GGTGTCAGTCCTCTAGGACGCCCCGCCCCGTTGCTCAGTACCTCCTCGGAGTCGGGTTGTTTGTGA
ATGCAGCCCAAAGTGGGTGGTAAACTCCATCTAAGGCTAAATACTGGCACGAGACCGATAGCAAAC
AAGTACCGTGAGGGAAAGTTGAAAAGAACTTTGAAGAGAGAGTTCAAGAGTACGTGAAACCGTATA
GAAGTAAACGGATGGGACCGCAGTGGACCGGCGGGATTCAGTTTTTGCGGGGCGGCTCGGTGACCT
TCACGGGGAATGCACTTTCCGTCGGGACAGCGCCACGACCGGTTTCTCGGCCGCCAGGCCCGTGGG
GAAGGTAGCTCGAGTATTATAGCCCCGCGGTGTTCCGGCCGGAGAGACCGAGGAATACCCGCCGGA
GACCGGTGGACTGGTGAGGACTGCTCGCAGTGCTCGCCGACCGCGCCGTTCGGGCCCCCCAGGGTC
AGTGGCGAAGTTGGCTCCCCATCCGACC

>Polydora_hoplura
CGCCTCTAGATTAGAGGTCCATTCTGCCCTATGTAAATGGCCGCGGTAGCCTGACCGTGCTAAGGT
AGCATAATCATTTGCCGTTTAATTGATGGCTGGAATGAATGAATATACGAGAGCTCAACTGTCTCA
TTTTTAATATAAATTGACCCTCAAGTGAAGAGACTTGAATTCTTAAAAAAGACGAAAAGACCCTAT
AGAGCTTTACCTGAAATATCTCATATAATAAAGGTTTAGTTGGGGCAACTAGAGAGCACTAAAACC
TCTCTTTAAATTTAAGTGATCCGTTACGATAGTAGAAAAAGCTACCTTAGGGATAACAGGCTAATC
CTCTTTAAGAGACCATATTGACAAGAGGGATTGGCACCTCGATGTTGGCTCAAGGTTACAGTTTGA
TGCAGCAGTAAGTAGGTTTGTTCACCCTTTAAATCCTTATATGCTTGTCTCAAAGACTAAGCCATG
CATGTCTAAGTATAAGCTCTACATGGTGAAACCGCGAATGGCTCATTAAATCAGTTATGGTTCCTT
AGATCTTAAAATCCTACTTGGATAACTGTAGTAATTCTAGAGCTAATACATGCACCTGAGCTCCGA
CCCTCGGGAAAGAGCGCTTTTATTAGACCAAAACCAATGGTCCGTGCCAACCTTTGGTGACTCTGG
ATAACTTC-AGCTGATCGCATGGCCGAGCCGGCGACGTATCTTTCAAATGTCTGCCCTATCAAGTT
TCGATGGTTGGTGACTTGCCAACCATGCTCGTAACGGGTAGCGGGGAATCAGGGTTCGATTCCGGA
GAGGGAGCATGAGAAACGGCTACCACATCCAAGGAAGGCAGCAGGCGCGCAAATTACCCACTCCTA
GCACGGGGAGGTAGTGACGAAAAATAACAATACGGGACTCTTACGAGGCCCCGTAATTGGAATGAG
TACACTTTAAATCCTTTAACGAGGATCTATTGGAGGGCAAGTCTGGTGCCAGCAGCCGCGGTAATT
CCAGCTCCAATAGCGTATATTAAAGTTGTTGCAGTTAAAAAGCTCGTAGTTGGATCTCGGGAGCGG
TCCGGCGGTCCTCCTCACGGAGGTTCACTGCCCGCCGCTCTCCTCACCGGCTTCCCCGGGTGCTCT
TTGCTGAGGCGGCCGGAATGTTTACTTTGAAAAAATTAGAGTGTTCAAAGCAGGCCCTGAATATTG
GCGCATGGAATAATGGAATAGGACCTCGGATTTTGTTGGTTTTCGGAAGTCGAGGTAATGATTAGT
AGGGACGACGGGGGCATTCGTATTGCACGGTTAGAGGTGAAATTCTTGGATCCGTGCAAGACGAAC
TACAGCGAAGGCGTCTGCCAAGTGCGTCTTCATTAATCAAGAGCGACAGTCAGAGGTTCGAAGACG
ATCAGATACCGTCGTAGTTCTGACCATAAACGATGCCAGCTAGGGATGGGCGGACGTTGTTCGACT
CCGCCCGGACCTTCCGGGAAACCAAAGCTTTTGGGTTCCGGGGGAAGTATGGTTGCAAAGCTGAAA
CTTAAAGGAATTGACGGAAGGGCACCACCAGGAGTGGAGCCTGCGGCTTAATTTGACTCAACACGG
GGAAACTCACCCGGCCCGGACACCGTAAGGATTGACAGATTGAAAGCTCTTTCTTGATTCGGTGGG
TGGTGGTGCATGGCCGTTCTTAGTTGGTGGAGCGATTTGTCTGGTTAATTCCGATAACGAACGAGA

CTCTAGCCTGCTAAATAGTTCGCCGGTTAGCCGGCGCAACTTCTTAGAGGGACAAGTGGCGTTTAG
CCACGCGAGATTGAGCAATAACAGGTCTGTGATGCCCTTAGATGTTCGGGGCCGCACGCGCGCTAC
ACTGAAGGACTCAGCTGGT--TCTCCTGGCTTGAAAAAGTCGGGCAACCCGTTGAACCTCCTTCGT
GCTAGGGATTGGGGTTTGCAATTCTTCCCCATGAACGAGGAATTCCCAGTAGGCGCGAGTCATCAG
CTCGCGTCGATTACGTCCCTGCCCTTTGTACACACCGCCCGTCGCTACTACCGATCGGATGGTTTA
GTGAGACCCTTGGACTGGTCCCGTCAGTGGGCAACCACCGACGCGTTGCCGGGAAGAAGGTCGAAC
TTGACTATCTAGAGGAAGTAAAAGTTCACTAAGCGGAGGAAAAGAAACTAACCAGGATTCCCTTAG
TAACGGCGAGTGAAACGGGAAGAGCCCAGCACCGAATCCCCTGCCTGC---GCAGCGGGAACTGTG
GTGTTTAGGAGAGCCTGTGGAGCGGCGG-AGCGTCCGAGTCCTTATGAAAGGGGACAGCCAGAGCG
GGTGTCAGTCCTCTAGGACGCCCCGTCCCGTTGCTCAGTACCTCCTCGGAGTCGGGTTGTTTGTGA
ATGCAGCCCAAAGTGGGTGGTAAACTCCATCTAAGGCTAAATACTGGCACGAGACCGATAGCAAAC
AAGTACCGTGAGGGAAAGTTGAAAAGAACTTTGAAGAGAGAGTTCAAGAGTACGTGAAACCGTATA
GAAGTAAACGGATGGGACCGCAGTGGACCGGCGGGATTCAGTTCCTTCGGGGCGGATTGGAGACCT
TCACCGGGAATGCACTTTCCGCCGGGACAGCGCCACGACCGGTTTCCCGGCCGCCAGGCCCGTTGG
GAAGGTAGCTCGAGTGTTATAGCCCTTCGGTGTTCCGGCCGGGGAGACCGAGGAATTCCCGCCGGA
GACCGGCGGACTGGTGAGGACTGCTCGCAGTGCTCGCCGACCGCGCCGTTCGGGCCCCCCAGGGTC
AGTGGCGAAGTTGGCTCCCCATCCGACC

>Polydora_onagawaensis
CGCCTCCAGATTGAAGGTCCATTCTGCCCTATGTAAATGGCCGCGGTAGCCTGACCGTGCTAAGGT
AGCATAATCATTTGCCGTTTAATTGATGGCTGGAATGAATGAATATACGAGAGCTAAGCTGTCTCA
TTTTTAATAAAAATTGACCTTTAAGTGAAGAGACTTAAATCTTAAAAAAAGACGAAAAGACCCTAT
AGAGCTTTATCTAAACTAATATTTATAGTAAAGATTTAGTTGGGGCAACTAGAGAGAACTAAAACC
TCTCTTTCTTTAAAAATGATCCGTTACGATGGAAGAAAAAGCTACCTTAGGGATAACAGGCTAATC
CTCTTTAAGAGACCATATTGACAAGAGGGATTGGCACCTCGATGTTGGCTCAAGGTTACAGTTTGA
TGCAGCAGTAAGTAGGTTTGTTCACCCTTTAAATCCTTATATGCTTGTCTCAAAGACTAAGCCATG
CATGTCTAAGTATAAGCTCTACATGGTGAAACCGCGAATGGCTCATTAAATCAGTTATGGTTCCTT
AGATCTTAAAATCCTACTTGGATAACTGTAGTAATTCTAGAGCTAATACATGCACCTGAGCTCCGA
CCCTCGGGAAAGAGCGCTTTTATTAGACCAAAACCAATGGTTCGTGCCAACCTTTGGTGACTCTGG
ATAACTTC-AGCTGATCGCATGGCCGTGCCGGCGACGTATCTTTCAAATGTCTGCCCTATCAAGTT
TCGATGGTTGGTGACTTGCCAACCATGCTCGTAACGGGTAGCGGGGAATCAGGGTTCGATTCCGGA
GAGGGAGCATGAGAAACGGCTACCACATCCAAGGAAGGCAGCAGGCGCGCAAATTACCCACTCCTA
GTTCGGGGAGGTAGTGACGAAAAATAACAATACGGGACTCTTTCGAGGCCCCGTAATTGGAATGAG
TACACTTTAAATCCTTTAACGAGGATCTATTGGAGGGCAAGTCTGGTGCCAGCAGCCGCGGTAATT
CCAGCTCCAATAGCGTATATTAAAGTTGTTGCAGTTAAAAAGCTCGTAGTTGGATCTCGGGAGCGG
TCCGGCGGTCCTCCTCGCGGAGGTTCACTGCCTGCCGCTCTCCTCACCGGCTTCTCCGGGTGCTCT

TCGCTGGGGCGGCCGGAATGTTTACTTTGAAAAAATTAGAGTGTTCAAAGCAGGCCCTGAATATTG
GCGCATGGAATAATGGAATAGGACCTCGGATTTTGTTGGTTTTCGGAAGTCGAGGTAATGATTAGT
AGGGACGACGGGGGCATTCGTATTGCACGGTTAGAGGTGAAATTCTTGGATCCGTGCAAGACGAAC
TACAGCGAAGGCGTCTGCCAAGTGCGTCTTCATTAATCAAGAGCGACAGTCAGAGGTTCGAAGACG
ATCAGATACCGTCGTAGTTCTGACCATAAACGATGCCAGCTAGGGATGAGAGGACGTTGTTCGACT
CCTCTCGGACCTTTCGGGAAACCAAAGCTTTTGGGTTCCGGGGGAAGTATGGTTGCAAAGCTGAAA
CTTAAAGGAATTGACGGAAGGGCACCACCAGGAGTGGAGCCTGCGGCTTAATTTGACTCAACACGG
GGAAACTCACCCGGCCCGGACACCGTAAGGATTGACAGATTGAAAGCTCTTTCTTGATTCGGTGGG
TGGTGGTGCATGGCCGTTCTTAGTTGGTGGAGCGATTTGTCTGGTTAATTCCGATAACGAACGAGA
CTCTAGCCTGCTAAATAGTTCGCCGGTTTGCCGGTGTAACTTCTTAGAGGGACAAGTGGCGTTTAG
CCACGCGAGATTGAGCAATAACAGGTCTGTGATGCCCTTAGATGTTCGGGGCCGCACGCGCGCTAC
ACTGAAGGACTCAGCTGGT--TCTCCTGGCCTGAAAAGGTCGGGCAACCCGTTGAACCTCCTTCGT
GCTAGGGATTGGGGTTTGCAATTCTTCCCCATGAACGAGGAATTCCCAGTAGGCGCGAGTCATCAG
CTCGCGTCGATTACGTCCCTGCCCTTTGTACACACCGCCCGTCGCTACTACCGATCGGATGGTTTA
GTGAGATCCTTGGACTGGTCCCGTCAGTGGGCAACCGCCGACGCGTTGCCGGGAAGAAGATCAAAC
TTGATTATCTAGAGGAAGTAAAAGT-CACTAAGCGGAGGAAAAGAAACTAACCAGGATTCCCTTAG
TAACGGCGAGTGAAACGGGAAGAGCCCAGCACCGAATCCCCTGCCTGT---GCAGCGGGAACTGTG
GTGTTTAGGAGAGCCTGTGGAGCGGCGG-AGCGTCCGAGTCCTTATGAAAGGGGACAGCCAGAGCG
GGTGTCAGTCCTCTAGGACGCCCCGTGCCGTTGCTCAGTACCTCCTCGGAGTCGGGTTGTTTGTGA
ATGCAGCCCAAAGTGGGTGGTAAACTCCATCTAAGGCTAAATACTGGCACGAGACCGATAGCAAAC
AAGTACCGTGAGGGAAAGTTGAAAAGAACTTTGAAGAGAGAGTTCAAGAGTACGTGAAACCGTATA
GAAGTAAACGGATGTGACCGCAATGGACCGGCGGGATTCAGTTTTCACGGGGCGGGACGGAGACCT
TCACCGGGAATGCACTTTCCGCCGGGACAGCGCCACGACCGGTTTTTCGGCCGCCAGGCCCGTGGG
GAAGGTAGCTCGAGTATTATAGCCCCGCGGTGTTCCGGCCGGAGAGACCGAGGAATACCCGCCGGA
GACCGGTGGACTGGTGAGGACTGCTCGCAGTGCTCGTCGACCGCGCCGTTCGGGCCCCCCAGGGTC
AGTGGCGAAGTTGGTTCCATATCCGACC

>Polydorella_dawydoffi

-GCATAATCATTTGCCGTTTAATTGATGGCTGGAATGAACGAATATACGAAAGCTTCCCTGTCTCA
ATTTTAATAAAAACTGACCTCCAAGTGAAGAGACTTGGGTAAACAAAAAAGACGAAAAGACCCTAT
AGAGCTTTACTTAAACCAATCCTCCAGGAGAAAGTTTAGTTGGGGCAACTAGAGAAAACTAAAACT
TCTCTTTACATAACTAAGATCCGATACGATAACAGAAAAAGCTACCTTAGGGATAACAGGCTAATC
CTCTTTGAGAGACCATATTGACAAGAGGGG------------------------------------
--------------------------------------????????????????????????????
??

??
??
??
??
??
??
??
??
??
??
??
??
??
??
??
??
??
??
??
??
??
??
??
??
?????????????????????????TCACTAAGCGGAGGAAAAGAAACTAACCAGGATTCCCTTAG
TAACGGCGAGTGAACCGGGAAGAGCCCAGCACCGAATCCCCTGCATCGACTGCAGCGGGAACTGTG
GTGTTTAGGAGAGCCTGTGGAGCGGCGG-AGCGTCCGAGTCCTCATGAACGGGGTTCGCCAGAGCG
GGTGTCAGTCCTCTAGGATGCACCGTCCCGTTGCTCAGTACCTCCTCGGAGTCGGGTTGTTTGAGA
TTGCAGCCCAAAGTGGGTGGTAAACTCCATCTAAGGCTAAATACTGGCACGAGACCGATAGCAAAC
AAGTACCGTGAGGGAAAGTT--

>Prionospio_dubia

??

??

??

??

??

??

????????????????????????????????ATATGCTTGTCTCAAAGATTAAGCCATG

CATGTCTAAGTACAAGCTTAACACGGTGAAACCGCGAATGGCTCATTAAATCAGTTATGGTTCCTT

AGATCGTACAATCCTACTTGGATAACTGTAGTAATTCTAGAGCTAATACATGCAAGCAAGCTCCGA

CCTTCGGGAAAGAGCGCTTTTATTAGACCAAAACCAAT--GGGCAACCTCTCGTTGGTGACTCTGG

ATAACTTCGAGCTGATCGCACGGCCGAGCCGGCGACGTATCTTTCAAATGTCTGCCCTATCAACTT

TCGTCGGTAGGCGCCATGCCTACGGAGGTAGTAACGGGTAACGGGGAATCAGGGTTCGATTCCGGA

GAGGGAGCATGAGAAACGGCTACCACATCCAAGGAAGGCAGCAGGCGCGCAAATTACCCACTCCTG

GCACGGGGAGGTAGTGACGAAAAATAACAATACGGGACTCTTTCGAGGCCCCGTAATTGGAATGAG

TACACTTTAAATCCTTTAACGAGGATCTATTGGAGGGCAAGTCTGGTGCCAGCAGCCGCGGTAATT

CCAGCTCCAATAGCGTATATTAAAGTTGTTGCAGTTAAAAAGCTCGTAGTTGGATCTCGGGAGCAG

GCCGGCGGTCCGCCTCGCGGCGG-CCACTGCCGGCCCTGCCCCCTGCCGGCTTCCCCCGGTGCTCT

TCGCTGGGGTGGCTGGTACGTTTACTTTGAAAAAATTAGAGTGTTCAAAGCAGGCCCTGAATAATG

GTGCATGGAATAATGGAATAGGACCTCGGATTTTGTTGGTTTTCGGAACTGGAGGTAATGATTAAG

AGGGACAGCGGGGGCATTCGTATTGCGCCGTTAGAGGTGAAATTCTTGGATCGGCGCAAGACGAAC

TACTGCGAAAGCATTTGCCAAGAATGTTTTCATTAATCAAGAACGAAAGTCAGAGGTTCGAAGACG

ATCAGATACCGTCGTAGTTCTGACCATAAACGATGCCAGCTAGGGATTCGCCGGAGTTGTATGACT

CTGCGAGCACCTTCCGGGAAACCAAAGCTTTTGGGTTCCGGGGGAAGTATGGTTGCAAAGCTGAAA

CTTAAAGGAATTGACGGAAGGGCACCACCAGGAGTGGAGCCTGCGGCTTAATTTGACTCAACACGG

GGAAACTCACCCGGCCCGGACACTGTAAGGATTGACAGATTGAGAGCTCTTTCTTGATTCGGTGGG

TGGTGGTGCATGGCCGTTCTTAGTTGGTGGAGCGATTTGTCTGGTTAATTCCGATAACGAACGAGA

CTCTAGCCTATTAAATAGTTCGCGGGTTCGCCCGCGCAACTTCTTAGAGGGACAAGTGGCGTTTAG

CCACGCGAGATTGAGCAATAACAGGTCTGTGATGCCCTTAGATGTTCGGGGCCGCACGCGCGCTAC

ACTGAAGGAATCAGCGTGTGTTCTCCCGGCCGGAAATGGTCGGGTAACCCGTTGAACCTCCTTCGT

GCTAGGGATTGGGGCTTGAAATTCTTCCCCATGAACGAGGAATTCCCAGTAAGCGCGAGTCATCAG

CTCGCGTTGATTACGTCCCTGCCCTTTGTACACACCGCCCGTCGCTACTACCGATTGGATGGTTTA

GTGAGACCCTCGGATTGGTCCCGGCGGCGGGCAACCGCCGCCGCGGCGCCGAGAAGACGGTCAAAC

TTGACTATCTAGAGGAAGTAAAAGTTCACTAAGCGGAGGAAAAGAAACTAACCAGGATTCCCCTAG

TAACGGCGAGTGAAGCGGGAAGAGCCCAGCACCGAATCCCCTGCGT--AACGYAGCGGGAACTGTG

GTGTTTGGGAGAGCCTGTGGGGCGCCGGCGGCGTCCGAGTCCTCCTGATCGGGGTCACCCAGAGCG

GGTGTCAGGCCTCTACGGCTTCTGCCAGCGTCCCTCTGAGCCTCCTAGGAGTCGGGTTGTTTGTGA
ATGCAGCCCAAAGTGGGTGGTAAACTCCATCTAAGGCTAAATACTGGCACGAGTCCGATAGCGGAC
AAGTACCGTGAGGGAAAGTTGAAAAGAACTTTGAAGAGAGAGTTCAAGAGTACGTGAAACCGTATA
GAGGCAAACGGATGGGGCCGCAGTCGACCCGCGGAATTCAGCTTTGCCGGTTTGGCTCGGAGCCTC
TTACGGCGAGTGCACTTTCCGCGGGGAGAGCGCCACGACCGGTTCTCGGGCCGTGAAGCCCTCCGG
GAAGGTAGCTTGAGTGTTATAGCCCGGGGAGGTGTTCGCCGGGGGGACCGAGGAACCCGCGCCGGG
CAGCAATGGACTGGCAGGGACTGCTCGCAGTGCTTGCCGACCGCGGTCATCGCATAACCGAGGGTC
AGTGGCGAAGTCGGTTTCCCATCCGACC

>Prionospio_sp_C_sensu_Guggolz_et_al_2020

-GCATAATCATTTGCCTTTTAATTAGAGGCTTGTATGAACGGACACACGTAAGCTAAGCTGTCTCA
TTAATAATAAAAATTAACCTCTAGGTGAAGAGACCTAGATCATATAAAAAGACGAAAAGACCCTAT
GGAGCTTTACTTACGTTATAATTTTCTTATAAAGTTTAGTTGGGGCGACTAAGGAGCAAAAAAACC
CTCCTCTTAATAAAATAGATCCGAAGCGA-TAAAAAATCAGCTACCCTAGGGATAACAGGCTAATC
CTCCTTGAGAGCCCAAATTGACAGGAGGGGTTGGCACCTCGATGTTGGCTTAAGGTTCCTCCAAAG
TGCAGCAGCAAGTAGGTTTGTTCACCCTTTAAAACCTT--------------------------TG
CATGTCT-AGTACAAGCTTAACATGGTGAAACCGCGAATGGCTCATTAAATCAGTTATGGTTCCTT
AGATCGTACAATCCTACTTGGATAACTGTAGTAATTCTAGAGCTAATACATGCAAGCAAGCTCCGA
CCTTCGGGAAAGAGCGCTTTTATTAGACCAAAACCAAT--GGGCAACCTCT-GTTGGTGACTCTGG
ATAACTTTGAGCTGATCGCATGGCCGAGCCGGCGACGTATCTTTCAAATGTCTGCCCTATCAACTT
TCGACGGTACGTGATATGCCTACCGTGGTTGTAACGGGTAACGGGGAATCAGGGTTCGATTCCGGA
GAGGGAGCATGAGAAACGGCTACCACATCCAAGGAAGGCAGCAGGCGCGCAAATTACCCACTCCTG
GCACGGGGAGGTAGTGACGAAAAATAACAATACGAGACTCTTTCGAGGCCTCGTAATTGGAATGAG
TACACTTTAAATCCTTTAACGAGGATCTATTGGAGGGCAAGTCTGGTGCCAGCAGCCGCGGTAATT
CCAGCTCCAATAGCGTATATTAAAGTTGTTGCAGTTAAAAAGCTCGTAGTTGGATCTCGGGAGCAG
GCCGGCGGTCCGCCTCGCGGCGG-TTACTGCCTGTCCTGCCCCCTGCCGGTTTCCCCCGGTGCTCT
TCACTGGGGTGGCCGGAACGTTTACTTTGAAAAAATTAGAGTGTTCAAAGCAGGCCCTGAATAATG
GTGCATGGAATAATGGAATAGGACCTCGGATTTTGTTGGTTTTCGGAACTTGAGGTAATGATTAAG
AGGGACAGCGGGGGCATTCGTATTACGCTGTTAGAGGTGAAATTCTTGGATCGGCGTAAGACGAAC
TACTGCGAAAGCATTTGCCAAGAATGTTTTCATTAATCAAGAACGAAAGTCAGAGGTTCGAAGACG
ATCAGATACCGTCGTAGTTCTGACCATAAACGATGCCAGCTAGGGATTCGCCGGAGTTGTATGACT
CTGCGAGCACCTTCCGGGAAACCAAAGCTTTTGGGTTCCGGGGGAAGTATGGTTGCAAAGCTGAAA
CTTAAAGGAATTGACGGAAGGGCACCACCAGGAGTGGAGCCTGCGGCTTAATTTGACTCAACACGG
GGAAACTCACCCGGCCCGGACACTGTAAGGATTGACAGATTGAGAGCTCTTTCTTGATTCGGTGGG
TGGTGGTGCATGGCCGTTCTTAGTTGGTGGAGCGATTTGTCTGGTTAATTCCGATAACGAACGAGA

CTCTAGCCTATTAAATAGTTCGCCGATTCGTCGGCGCAACTTCTTAGAGGGACAAGTGGCGTTTAG
CCACGCGAGATTGAGCAATAACAGGTCTGTGATGCCCTTAGATGTTCGGGGCCGCACGCGCGCTAC
ACTGAAGGAATCAGCGTGTGTTCTCCTGGCCAGAAATGGTCGGGTAACCCGTTGAACCTCCTTCGT
GCTAGGGATTGGGGTTTGCAATTCTCCCCCATGAACGAGGAATTCCCAGTAAGCGCGAGTCATCAG
CTCGCGTTGATTACGTCCCTGCCCTTTGTACACACCGCCCGTCGCTACTACCGATTGGATGGTTTA
GTGAGACCCTCGGATTGGTCCCGACGGTGGGTAACCGCCGTCGTGGTGC-----------------
-------------------------???
??
??
??
??
??
??
??
??
??
????????????????????????????

>Prionospio_sp_E_sensu_Guggolz_et_al_2020
CGCCACTAGATTAGAGGTCCATCCTGCCCGGTGTAAACGGCCGCGGTATCCTGACCGTGCTAAGGT
AGCATAATAATTTGCCTTTTAATTGGAGGCTAGCATGAACGGACAAACGAGAATATGGCTGTCTCA
CTACCAATGAAAATTAACCTTCAAGTGAAGAGGCTTGAATCACATAAAAAGACGAAAAGACCCTAT
GGAGCTTTACTTACATAGCCAAACTCCTAAATAGTTCAGTTGGGGCGACTGAGGAGCAATTAAAGC
CCCCTCAACACGCAAAGAATCCAACGTGA-TAAAGAATCAGCTACCCTAGGGATAACAGGCTAATC
CTTCTCGAGAGCCCGAATTGACAGAAGGGGTTGGCACCTCGATGTTGGCTTAAGGTACCTCTAAAG
TGCAGCAGCTAGTAGGTTTGTTCAACCTTTAAAACCTTATATGCTTGTCTCAAAGACTAAGCCATG
CATGTCTAAGTACAAGCTTAACATGGTGAAACCGCGAATGGCTCATTAAATCAGTTATGGTTCCTT
AGATCGTACAATCCTACTTGGATAACTGTAGTAATTCTAGAGCTAATACATGCAAGCAAGCTCCGA
CCTTCGGGAAAGAGCGCTTTTATTAGACCAAAACCAAT--GGGCAACCTCGTGTTGGTGACTCTGG
ATAACTTTGAGCTGATCGCATGGCCGAGCCGGCGACGTATCTTTCAAATGTCTGCCCTATCATCTT
TCGACGGTATGTGATCTGCTTACCGTGGATATAACGGGTAACGGGGAATCAGGGTTCGATTCCGGA
GAGGGAGCATGAGAAACGGCTACCACATCCAAGGAAGGCAGCAGGCGCGCAAATTACCCACTCCTG
GCACGGGGAGGTAGTGACGAAAAATAACAATACGGGACTCTTTCGAGGCCCCGTAATTGGAATGAG
TACACTTTAAATCCTTTAACGAGGATCTATTGGAGGGCAAGTCTGGTGCCAGCAGCCGCGGTAATT
CCAGCTCCAATAGCGTATATTAAAGTTGTTGCAGTTAAAAAGCTCGTAGTTGGATCTCGGGAGCAG
GCCGGCGGTCCGCCTCGCGGCGG-TTACTGCCTGTCCTGCCCCCTGCCGGTTTCCCCCGGTGCTCT

TCGCTGGGGTGGCCGGAACGTTTACTTTGAAAAAATTAGAGTGTTCAAAGCAGGCCCTGAATAATG
GTGCATGGAATAATGGAATAGGACCTCGGATTTTGTTGGTTTTCGGAACTTGAGGTAATGATTAAG
AGGGACAGCGGGGGCATTCGTATTGCACTGTTAGAGGTGAAATTCTTGGATCGGTGCAAGACGAAC
TACTGCGAAAGCATTTGCCAAGAATGTTTTCATTAATCAAGAACGAAAGTCAGAGGTTCGAAGACG
ATCAGATACCGTCGTAGTTCTGACCATAAACGATGCCAGCTAGGGATTCGCCGGAGTTGTATGACT
CGGCGAGCACCTTCCGGGAAACCAAAGCTTTTGGGTTCCGGGGGAAGTATGGTTGCAAAGCTGAAA
CTTAAAGGAATTGACGGAAGGGCACCACCAGGAGTGGAGCCTGCGGCTTAATTTGACTCAACACGG
GGAAACTCACCCGGCCCGGACACTGTAAGGATTGACAGATTGAGAGCTCTTTCTTGATTCGGTGGG
TGGTGGTGCATGGCCGTTCTTAGTTGGTGGAGCGATTTGTCTGGTTAATTCCGATAACGAACGAGA
CTCTAGCCTATTAAATAGTTCGCCGATTCGTCGGCGCAACTTCTTAGAGGGACAAGTGGCGTTTAG
CCACGCGAGATTGAGCAATAACAGGTCTGTGATGCCCTTAGATGTTCGGGGCCGCACGCGCGCTAC
ACTGAAGGACTCAGCGTGTGTTCTCCTGGCCCGAAAGGGTCGGGTAACCCGTTGAACCTCCTTCGT
GCTAGGGATAGGGGTTTGCAATTATCCCCCTTGAACGAGGAATTCCCAGTAAGCGCGAGTCATCAG
CTCGCGTTGATTACGTCCCTGCCCTTTGTACACACCGCCCGTCGCTACTACCGATTGGATGGTTTA
GTGAGACCCTCGGATTGGTCCCGACGGTGGGCGACCGCCGTCGTGGTGCCGAAAAGACGGTCAAAC
TTGACTATCTAGAGGAAGTAAAAGT???
??
??
??
??
??
??
??
??
??
????????????????????????????

>Prionospio_sp_KJO_2005
??
??
??
??
??
??
??????????????????????????????????????----------------------------
---GTTATGGTTCCTT

AGATCGTACAATCCTACTTGGATAACTGTAGTAATTCTAGAGCTAATACATGCAAGCAAGCTCCGA
CCTTCGGGAAAGAGCGCTTTTATTAGACCAAAACCAAT--GGGCAACCTCACGTTGGTGACTCTGG
ATAACTTTGAGCTGATCGCATGGCCGAGCCGGCGACGTATCTTTCAAATGTCTGCCCTATCAACTT
TCGACTGGACGTGATATGCCTCCAGTGGTTGTAACGGGTAACGGGGAATCAGGGTTCGATTCCGGA
GAGGGAGCATGAGAAACGGCTACCACATCCAAGGAAGGCAGCAGGCGCGCAAATTACCCACTCCTG
GCACGGGGAGGTAGTGACGAAAAATAACAATACGGGACTCTTTCGAGGCCCCGTAATTGGAATGAG
TACACTTTAAATCCTTTAACGAGGATCTATTGGAGGGCAAGTCTGGTGCCAGCAGCCGCGGTAATT
CCAGCTCCAATAGCGTATATTAAAGTTGTTGCAGTTAAAAAGCTCGTAGTTGGATCTCGGGAGCAG
GCCGGCGGTCCGCCTCGCGGCGG-TTACTGCCTGTCCTGCCCCCTGCCAGTTTCCCCCGGTGCCCT
TCGCTGGGGTGGCTGGAACGTTTACTTTGAAAAAATTAGAGTGTTCAAAGCAGGCCCTGAATAATG
GTGCATGGAATAATGGAATAGGACCTCGGATTTTGTTGGTTTTCGGAACTTGAGGTAATGATTAAG
AGGGACAGCGGGGGCATTCGTATTACGGTGTTAGAGGTGAAATTCTTGGATCGCCGTAAGACGAAC
TACTGCGAAAGCATTTGCCAAGCATGTTTTCATTAATCAAGAACGAAAGTCAGAGGTTCGAAGACG
ATCAGATACCGTCGTAGTTCTGACCATAAACGATGCCAGCTAGGGATTCGCCGGAGTTGTATGACT
CGGCGAGCACCTTCCGGGAAACCAAAGCTTTTGGGTTCCGGGGGAAGTATGGTTGCAAAGCTGAAA
CTTAAAGGAATTGACGGAAGGGCACCACCAGGAGTGGAGCCTGCGGCTTAATTTGACTCAACACGG
GGAAACTCACCCGGCCCGGACACTGTAAGGATTGACAGATTGAGAGCTCTTTCTTGATTCGGTGGG
TGGTGGTGCATGGCCGTTCTTAGTTGGTGGAGCGATTTGTCTGGTTAATTCCGATAACGAACGAGA
CTCTAGCCTATTAAATAGTTCGCCGATTCGTCGGCGCAACTTCTTAGAGGGACAAGTGGCGTTTAG
CCACGCGAGATTGAGCAATAACAGGTCTGTGATGCCCTTAGATGTTCGGGGCCGCACGCGCGCTAC
ACTGAAGGAATCAGCGTGTGTTCTCCTGGCCCGAAAGGGTCGGGTAACCCAATGAACCTCCTTCGT
GCTAGGGATTGGGGTTTGCAATTATCCCCCATGAACGAGGAATTCCCAGTAAGCGCGAGTCATCAG
CTCGCGTTGATTACGTCCCTGCCCTTTGTACACACCGCCCGTCGCTACTACCGATTGGATGGTTTA
GTGAGACCCTCGGATTGGTCCCGACGGTGGGCAACCGCCGTCGTG-CGCCGAAAAGACGGTCAAAC
TTGACTATCTAGAGGNAGTAAAAGT---

-------------------------CGGCGGCGTCCGAGTCCTCCTGATCGGGGTTACCCATAGCG
GGTGTCAGGCCTCTACGGCTTCCGCCGTCGTCCCTCTGAGCCTCCTAGGAGTCGGGTTGTTTGGGA
ATGCAGCCCAAAGTGGGTGGTAAACTCCATCTAAGGCTAAATACTGGCATGAGTCCGATAGCGGAC
AAGTACCGTGAGGGAAAGTTGAAAAGAACTTTGAAGAGAGAGTTCAAGAGTACGTGAAACCGTATA
GAGGCAAACGGATGGGGCCGCAGTCGACCCGCGGAATTCATCTTGCGACGGGTGCAGTTCGGAGCC
TCTCGGCGAGTGCACTTTCCGCGGGGAGAGCGCCACGACCGGTTCTCTGGCAGTCAAGCCCGTCGG
GAAGGTAGCTCGAGTGTTATAGCCCAGCGAGGTGTTGGCCTGGGGGACCGAGGAACACCCGCCGGG
ACGCGGCGGACCTGCAAGGACTGCTCGCAGTGCTTGCTAACCGCGTCGCGGGGTTCACCCAGGGTC
AGTGGCGAAGTCGGTTTCCCATCCGACC

>Pseudopolydora_paucibranchiata

CGCCTCTAGATTGGAGGTCCATTCTGCCCCATGTAAATGGCCGCGGTAACCTGACCGTGCTAAGGT

AGCATAATCACTTGCCGTTTAATTGATGGCTAGAATGAATGAATATACGAAAGCTCCGCTGTCTCG

CCCTAATTAAAAATTGGCCTTCAAGTGAAGAGACTTGAATTAAACAAAAAGACGAAAAGACCCTAT

AGAGCTTAGCTTAACACACCCTACTGAAAGAAAGCTTAGTTGGGGCAACTAGAGAAAACTAAAACC

TCTCTT----------AGATCCAAACTGAAAATAGAAAAAGCTACCTTAGGGATAACAGGCTAATC

TTGTTTGAGAGACCATATTGACAACAAGGCTTGGCACCTCGATGTTGGCTCAGGATTTCACTTTGA

TGCAGCAGTATGAAGGTTTGTTCTCCCTTTAATATCCTATATGCTTGTCTCAAAGACTAAGCCATG

CATGTCTAAGTATAAGCTCTACATGGTGAAACCGCGAATGGCTCATTAAATCAGTTATGGTTCCTT

AGATCGCACTATCCTACTTGGATAACTGTAGTAATTCTAGAGCTAATACATGCATCGAAGCTCCGA

CCTCGGGGAAAGAGCGCTTTTATTAGACCAAAACCAATTCGGCCGTCCAACCTTTGGTGAATCTGG

ATAACTTG-AGCTGATCGCACGGCCGAGCCGGCGACGTATCTTTCAAATGTCTGCCCTATCAAGTC

TCGACGGTTGGTGACTTGCCAACCGTGCTTATTACGGGTAGCGGGGAATCAGGGTTCGATTCCGGA

GAGGGAGCATGAGAAACGGCTACCACATCCAAGGAAGGCAGCAGGCGCGCAAATTACCCACTCCTA

GCTCGGGGAGGTAGTGACGAAAAATAACAATACGGGACTCTTACGAGGCCCCGTAATTGGAATGAG

TACACTTTAAATCCTTTAACGAGGATCTATTGGAGGGCAAGTCTGGTGCCAGCAGCCGCGGTAATT

CCAGCTCCAATAGCGTATACTAAAGTTGTTGCAGTTAAAAAGCTCGTAGTTGGATCTCGAGACCGG

CCCGTCGGTCCTCCTCGCGGAGGTTTACTGGCCGCCGGCCTCCTCGCCGGCTTCTGCAGGTGCTCT

TTACTGCGGCGGCCGGAGCGTTTACTTTGAAAAAATTAGAGTGTTCAAAGCAGGCCCTGAATACTG

GTGCATGGAATAATGGAATAGGACCTCGGATTTTGTTGGTTTTCGGAAGTCGAGGTAATGATTAGT

AGGGACGGCGGGGGCATTCGTATTGCGCGGTTAGAGGTGAAATTCTTGGATCCGTGCAAGACGAAC

TACAGCGAAGGCGTTTGCCAAGTGCGTCTTCATTAATCAAGAGCGACAGTCAGAGGTTCGAAGACG

ATCAGATACCGTCGTAGTTCTGACCATAAACTCTGCCAGCTAGGGATGGGCGGTCGTTGAACGACT

CCGCCCGGACCTTCCGGGAAACCAAAGCTTTTGGGTTCCGGGGGAAGTATGGTTGCAAAGCTGAAA

CTTAAAGGAATTGACGGAAGGGCACCACCAGGAGTGGAGCCTGCGGCTTAATTTGACTCAACACGG

GGAAACTCACCCGGCCCGGACACCGTAAGGATTGACAGATTGAAAGCTCTTTCTTGATTCGGTGGG

TGGTGGTGCATGGCCGTTCTTAGTTCGTGGAGCGATTTGTCTGGTTAATTCCGATAACGAACGAGA

CTCTAGCCTGCTAAATAGTTCGCCGGTATGCCGGTGCGACTTCTTAGAGGGACAAGTGGCGTTTAG

CCACGCGAGATTGAGCAATAACAGGTCTGTGATGCCCTTAGATGTTCGGGGCCGCACGCGCGCTAC

ACTGAAGGAATCAGCTGGTGCTCTCCTGGTCTGGAAAGATCGGGCAACCCGTTGAACCTCCTTCGT

GCTAGGGATTGGGGCTTGCAATTGTTCCCCATGAACGAGGAATTCCCAGTAGGCGCGAGTCATCAG

CTCGCGTCGATTACGTCCCTGCCCTTTGTACACACCGCCCGTCGCTACTACCGATCGGATGGTTTA

GTGAGACCCTTGGACTGGTCCCGTTGGTGGGCAACCACTAACGTGTTGCCGGGAAGAAGGTCGAAC

TTGACTATCTAGAGGAAGTAAAAGTTCACTAAGCGGAGGAAAAGAAACTAACAAGGATTCCCTTAG

TAACGGCGAGTGAACCGGGAAGAGCCCAGCACCGAATCCCCTGCATCGACTGTAGCGGGAACTGTG

GTGTTTAGGAGAGCCTGTGGAGCGGTGG-AGCGTCCGAGTCCTCACGAACGGGGTTTGCCAGAGCG

GGTGTCAGTCCTCTAGGACGCACCATCCCGTTGCTCAGTACCTCCTAGGAGTCGGGTTGTTTGAGA
TTGCAGCCCAAAGCGGGTGGTAAACTCCATCTAAGGCTAAATACTGGCACGAGACCGATAGCAAAC
AAGTACCGTGAGGGAAAGTTGAAAAGAACTTTGAAGAGAGAGTTCAAGAGTACGTGAAACCGTATA
GAAGCAAACTGATGGAACCGCAGTGGACTGGCGGGATTCAGCTTTTCT-GGGCGGTGTGTCGACCC
TAACAGGGAGTGCACTTTCCGCCAGGACAGCGCCACGTCCGGTTCTTCTGCCGCCAGGCCCGGGAG
GAAGGTAGCTTAAGTATTATAGCCTCTCGGTGTTCCGGCCGGAGGGACCGTGGAATACCCGCCGGA
GACCGGTCGACTGGTGAGGACTGCACGCAGTGCTCGCCGACTGCGCTGTTCGGGCCCCCCAGGGAC
TGTGGCGAAGTTGGCTCTCCATCAGACC

>Pseudopolydora_tsubaki
CGCCTCTAGATTAGAGGTCCATTCTGCCCTATGTAAATGGCCGCGGTAACCTGACCGTGCTAAGGT
AGCATAATCATTTGCCATTTAATTGGTGGCTAGTATGAACGAATATACGAGATCAGAACTGTCTCT
TGTTTACTAAAAATTGACCTTCAAGTGAAGAGACTTGACTTAAATAAAAAGACGAAAAGACCCTAT
AGAGCTTTATTTTAACTACTCTCTTAAGTGAAAATTTAGTTGGGGCAACTAGAGGGAACTAAAACA
CCTCTAAAATAAAATAAGATCCACGCTGATAAAAGAAAAAGCTACCTTAGGGATAACAGGCTAATC
CTGTTTTAGAGACCATATTGACAACAGGGCTTGGCACCTCGATGTTGGCTCAAGGCAACCCCCCGA
TGCAGCAGTAGGTAGGTTTGTTCACCCTTTAATTCCTT----------------------------
------TAAGTATAAGCTCAACATGGTGAAACCGCGAATGGCTCATTAAATCAGTTATGGTTCCTT
AGATCGTATCATCCTACTTGGATAACTGTAGTAATTCTAGAGCTAATACATGCACCGAAGCTCCGA
CCCTGGGGAAAGAGCGCTTTTATTAGACCAAAACCAATGGCGGCGGCCACCCTTTGGTGAATCTGG
ATAACTTC-AGCCGATCGCACGGCCGTGCCGGCGACGTATCTTTCAAATGTCTGCCCTATCAAGTT
TCGACGGTTCGTGACATGCCAACCGTGCTTATAACGGGTAGCGGGGAATCAGGGTTCGATTCCGGA
GAGGGAGCATGAGAAACGGCTACCACATCCAAGGAAGGCAGCAGGCGCGCAAATTACCCACTCCTA
GCTCGGGGAGGTAGTGACGAAAAATAACAATACGGGACTCTTACGAGGCCCCGTAATTGGAATGAG
TACACTTTAAATCCTTTAACGAGGATCTATTGGAGGGCAAGTCTGGTGCCAGCAGCCGCGGTAATT
CCAGCTCCAATAGCGTATATTAAAGTTGTTGCAGTTAAAAAGCTCGTAGTTGGATCTCGGGAGCCG
TCCGGCGGTCCTCCTCGCGGAGGTTCACTGCCCGCGGCTCTCCTCGCCGGCTTCCCCGGGTGCTCT
TCACTGAGGCGGCCGGAGCGTTTACTTTGAAAAAATTAGAGTGTTCAAAGCAGGCCCTGAATATTG
GCGCATGGAATAATGGAATAGGACCTCGGATTTTGTTGGTTTTCGGAAGTCGAGGTAATGATTAGT
AGGGACGACGGGGGCATTCGTATTGCGCGGTTAGAGGTGAAATTCTTGGATCCGCGCAAGACGAAC
TACAGCGAAGGCGTTTGCCAAGTGCGTCTTCATTAATCAAGAGCGACAGTCAGAGGTTCGAAGACG
ATCAGATACCGTCGTAGTTCTGACCATAAACGATGCCAGCCAGGGACGGGCGGACGTTGAACGACT
CCGCCCGGACCTTCCGGGAAACCAAAGCTTTTGGGTTCCGGGGGAAGTATGGTTGCAAAGCTGAAA
CTTAAAGGAATTGACGGAAGGGCACCACCAGGAGTGGAGCCTGCGGCTTAATTTGACTCAACACGG
GGAAACTCACCCGGCCCGGACACCGTAAGGATTGACAGATTGAAAGCTCTTTCTCGATTCGGTGGG
TGGTGGTGCATGGCCGTTCTTAGTTGGTGGAGCGATTTGTCTGGTTAATTCCGATAACGAACGAGA

CTCTAGCCTGCTAAATAGTTCGCCTCTGTGCTCGGGATACTTCTTAGAGGGACAAGTGGCTTTTAG
CCACGCGAGATTGAGCAATAACAGGTCTGTGATGCCCTTAGATGTTCGGGGCCGCACGCGCGCTAC
ACTGAAGGAATCAGCTGGTC-CTTCCTGGCCTGGAAAGGTCGGGCAACCCGTTGAACCTCCTTCGT
GCTAGGGATTGGGGCTTGCAATTATTCCCCATGAACGAGGAATTCCCAGTAGGCGCGAGTCATCAG
CTCGCGTCGATTACGTCCCTGCCCTTTGTACACACCGCCCGTCGCTACTACCGATCGGATGGTTTA
GTGAGACCCTCGGACTGGTCCCGTTGGGGGGCAACTCCTTTCGCGTTGCCGGGAAGAAGG------
-------------------------TCACTAAGCGGAGGAAAAGAAACTAACCAGGATTCCCTTAG
TAACGGCGAGTGAAACGGGAAGAGCCCAGCACCGAATCCCCTGCATCGACTGTAGAGGGAACTGTG
GTGTTTGGGAGAGACCGTGGAGCGGCGG-GGCTTCCGAGTCCTCATGAACGGGGTTCGCCAGAGCG
GGTGTCAGTCCTATAGGAGGCACCGTCCCGTTGCTCGGTCACTCCTAGGAGTCGGGTTGTTTGAGA
TTGCAGCCCTAAGCGGGTGGTAAACTCCATCTAAGGCTAAATACTGGCACGAGACCGATAGCAAAC
AAGTACCGTGAGGGAAAGTTGAAAAGAACTTTGAAGAGAGAGTTCAAGAGTACGTGAAACCGTATA
GAAGTAAACCGATGGAACCGCAGTGGACGGGCGGGATTCAGCTTTCTCTCGGC-GTCCGGGGACCG
TTAAAAGGAGTGCACTTTCCGCCCGAACAGCGCCACGACCGGTTCTCCGGCCGCCAGGCCCGAGAG
GAAGGTAACTCGAGTGCTATAGCCTCTCGAAG--CCGGCCGGGAGGACCGAGGAGTATCCGCCGGC
TTCCAGTAGACTGACGTGGACTGCTCGCAGTGCTCGTCGACTGCGCTTTTCGGGTCCAACAGGGTC
TGTGGCGAAGTTGGCCATCCATCGGACC

>Pygospio_elegans
CGCCTCTAGATTAGAGGTCCATTCTGCCCTATGTAAATGGCCGCGGTAACCTGACCGTGCTAAGGT
AGCATAATCATTTGCCATTTAATTGGTGGCTAGAATAAATGAATACACGAAAGTTAAGCTGTCTCA
TTTTTAATAAAAATTGACCCCCTGGTGAAGAGACCAGTATTAAACAAAAAGACGAAAAGACCCTAT
AGAGCTTTATCCTATATAATTATCTTAAAAAGGATTTAGTTGGGGCAACTAGAGAGAACTAAATCC
TCTCTTACAAAAACACTGATCCGAATCGACAGAAGAGAAAGCTACCTTAGGGATAACAGGCCAATC
CTTTTTGAGAGACCATATTGACAAAAGGGATTAGCACCTCGATGTTGGCTCAAGGTTACAGCTTGA
TGCAGCAGTAAGCGGGTTTGTTCACCCTTTAACCCTT--------------------CTAGCCATG
CATGTCTAAGTATAAGCTCTATATGGTGAAACCGCGAATGGCTCATTAAATCAGTTATGGTTTATT
AGATCTTAAAATCCTACTTGGATAACTGTAGTAATTCTAGAGCTAATACATGCAAAAAAGCTCCGA
CCTCGCGGAAAGAGCGCTTTTATTAGACCAAAACCAAAGGGGCCGTCTAACCTCTGGTGACTCTGG
ATAACTTC-AGCTGATCGCACGTCTAAGACGGCGACGTATCTTTCAAATGTCTGCCCTATCAAGTT
TCGATGGTTGTTGCTTTGCCAACCATGCTTATAACGGGTAGCGGGGAATCAGGGTTCGATTCCGGA
GAGGGAGCATGAGAAACGGCTACCACATCCAAGGAAGGCAGCAGGCGCGCAAATTACCCACTCCCA
GCTCGGGGAGGTAGTGACGAAAAATAACAATACGGGACTCTTACGAGGCCCCGTAATTGGAATGAG
TACAATTTAAATCCTTTAACGAGGATCTATTGGAGGGCAAGTCTGGTGCCAGCAGCCGCGGTAATT
CCAGCTCCAATAGCGTATATTAAAGTTGTTGCAGTTAAAAAGCTCGTAGTTGGATCTCGGGGACGG
TCCGGCGGTGCTCCTCGCGGAGTTATACTGCCCATCGTTCTCCTCGCCGGCTGTCTAGGGTGGACT

TCGTTGAGTTGGCCGGGACGTTTACTTTGAAAAAATTAGAGTGTTCAAAGCAGGCCCTGAATACTG

GTGCATGGAATAATGGAATAGGATCTCGGATTTTGTTGGTTTTTGGAAGTCGAGGTAATGATAAAT

AGGGACGACGGGGGCATTCGTATTGCACGGTTAGAGGTGAAATTCTTGGATCCGTGCAAGACGAAC

TACAGCGAAGGCGTCTGCCAAGTGCGTCTTCATTAATCAAGAGCGACAGTCAGAGGTTCGAAGACG

ATCAGATACCGTCGTAGTTCTGACCATAAACGATGCCAGCTAGGGATGGGCGGACGTTGCACGACT

CCGCCCGGACCTTCCGGGAAACCAAAGCTTTTAGGTTCCGGGGGAAGTATGGTTGCAAAGCTGAAA

CTTAAAGGAATTGACGGAAGGGCACCACCAGGAGTGGAGCCTGCGGCTTAATTTGACTCAACACGG

GGAAACTCACCCGGCCCGGACACCGTTAGGATTGACAGATTGAAAGCTCTTTCTTGATTCGGTGGG

TGGTGGTGCATGGCCGTTCTTAGTTGGTGGAGCGATTTGTCTGGTTAATTCCGATAACGAACGAGA

CTCTAGCCTGCTAAATAGTTCACCGGTTTGCCGGTGCAACTTCTTAGAGGGACAAGTGGCGTTCAG

CCACGCGAGATTGAGCAATAACAGGTCTGTGATGCCCTTAGATGTCCGGGGCCGCACGCGCGCTAC

ACTGAAGGAATCAACTGGT--CCTCCTGTCCTGAAAAGGATGGGTAACCCGATGAACCTCCTTCGT

GCTAGGGATTGGGGTTTGTAATTCTTCCCCATGAACGAGGAATTCCCAGTAAGCGCGAGTCATCAA

CTCGCGTTGATTACGTCCCTGCCCTTTGTACACACCGCCCGTCGCTACTACCGATCGGATGGTTTA

GTGAGACCCTCGGACCGGTCCCGTCAGCGGTTCGCCGCAGACGCGCTGCTGGGAAGAAGTTCGAAC

TTG----------------------TCACTAAGCGGAGGAAAAGAAACTAACCAGCA-TCCCTCAG

TAACGGCGAGTGAACCGGGAGGAGCCCAGCACCGAATCCCCTGCCGCTA-GGCAGCGGGAACTGTG

GTGTTAAGGAGAGCCTGTGGAGCAGCGG-TGCGTCCGAGTCCCCATGAACGGGGTTTGCCAGAGCG

GGTGTAAGTCCTCTAAGGCGCCCCGCACTGTTGCTCAGTACCTCCTAGGAGTCGGATTGTTTGAGA

ATGCAGTCCAAAGCGGGTGGTAAACTCCATCTAAGGCTAAATACTGGCACGAGTCCGATAGCGAAC

AAGTACCATGAGGGAAAGTT--

>Pygospio_sp_VVP_2014

-GCATAATCATTTGCCATTTAATTGGTGGCTAGAATAAATGAATACACGAAAATTAAGCTGTCTCA

CTTTTAATAAAAATTGATCTCCTGGTGAAGAAACCAGGATTCTACAAGAAGACGAAAAGACCCTAT

AGAGCTTTACCCTATATAATATTCATAAAAAGGGTTTAGTTGGGGCAACTAGAGAGAACTAAATCC

TCTCTTTTAAGAAAAAAGATCCGAATCGATAGAAGAAAAAGCTACCTTAGGGATAACAGGCCAATC

CTTTTTGAGAGACCATATTGACAAAAGGA-------------------------------------

--TAAGCCTG

CATGTCTAAGTATAAGCTCTATATGGTGAAACCGCGAATGGCTCATTAATACAATTATGGTTTATT

AGATCTTAAAATCCTACTTGGATAACTGTAGTAATTCTAGAGCTAATACATGCAAAATAGCTCCGA

CTTCACGGAAAGAGCGCTTTTATTAGACCAAAACCAAAGGGGCCGTCTAACCTCTGGTGACTCTGG

ATAACTTC-AGCTGATCGCACGTCTAAGACGGCGACGTATCTTTCAAATGTCTGCCCTATCAAGTT

TCGATGGTTGTTGCTATGCCAACCATGCTTATAACGGGTAGCGGGGAATCAGGGTTCGATTCCGGA

GAGGGAGCATGAGAAACGGCTACCACATCCAAGGAAGGCAGCAGGCGCGCAAATTACCCACTCCCA

GCTCGGGGAGGTAGTGACGAAAAATAACAATACGGGACTCTTACGAGGCCCCGTAATTGGAATGAG

TACAATTTAAATCCTTTAACGAGGATCTATTGGAGGGCAAGTCTGGTGCCAGCAGCCGCGGTAATT

CCAGCTCCAATAGCGTATATTAAAGTTGTTGCAGTTAAAAAGCTCGTAGTTGGATCTCGGGAGCGG

TCCGGTGGTCCTCCTCGCGGAGGTTTACTGCC-ATCGTTCTCCTCGCCGGCTGTCTAGGGTGAGCT

TCGTTGAGTTGGCCGGGACGTTTACTTTGAAAAAATTAGAGTGTTCAAAGCAGGCCCTGTATAATG

GTGCATGGAATAATGGAATAGGATTTCGGATTTTGTTGGTTATTGGAAGTCGAAATAATGATAAAT

AGGGACGACGGGGGCATTCGTATTGCACGGTTAGAGGTGAAATTCTCGGATCCGTGCAAGACGAAC

TACAGCGAAGGCGTCTGCCAAGTGCGTTTTCATTAATCAAGAGCGACAGTCAGAGGTTCGAAGACG

ATCAGATACCGTCGTAGTTCTGACCATAAACGATGCCAGCTAGGGATGGGCGGACGTTGCACGACT

CCGCCCGGACCTTCCGGGAAACCAAAGCTTTTAGGTTCCGGGGGAAGTATGGTTGCAAAGCTGAAA

CTTAAAGGAATTGACGGAAGGGCACCACCAGGAGTGGAGCCTGCGGCTTAATTTGACTCAACACGG

GGAAACTCACCCGGCCCGGACACCGTTAGGATTGACAGATTGAAAGCTCTTTCTTGATTCGGTGGG

TGGTGGTGCATGGCCGTTCTTAGTTGGTGGAGCGATTTGTCTGGTTAATTCCGATAACGAACGAGA

CTCTAGCCTGCTAAATAGTTCATCGGTTCGCCGGTGCAACTTCTTAGAGGGACAAGTGGCGTTCAG

CCACGCGAGATTGAGCAATAACAGGTCTGTGATGCCCTTAGATGTCCGGGGCCGCACGCGCGCTAC

ACTGAAGGAATCAACTGGT--CCTCCTGTCCTGAAAAGGATGGGTAACCCGATGAACCTCCTTCGT

GCTAGGGATTGGGGTTTGTAATTCTTCCCCATGAACGAGGAATTCCCAGTAAGCGCGAGTCATCAA

CTCGCGTTGATTACGTCCCTGCCCTTTGTACACACCGCCCGTCGCTACTACCGATTGGATGGTTTA

GTGAGATCCTCGGACCGGTCCCGTCAGCGGTTCGCCGCAGACGCGCTGCTGGGAAGAAGCTCGAAC

TTGGTCATCTAGAGGAAGTAAAAGT------------------------CATTCCCTCAG

TAACGGCGAGTGAACCGGGAGGAGCCCAGCACCGAATCCCGTGCCGCTA-GGCACCGGGAACTGTG

GTGTTAAGGAGAGCCTGTGGAGCAGCGG-TGCGTCCGAGTCCCCATGAACGGGGTTTGCCAGAGCG

GGTGTTAGTCCTCTATGGCGCCCCGCACTGTTGCTCAGTACCTCCTAGGAGTCGGATTGTTTGAGA

ATGCAGTCCAAAGCGGGTGGTAAACTCCATCTAAGGCTAAATACTGGCACGAGTCCGATAGCGAAC

AAGTACCATGAGGGAAAGTT--

>Rhynchospio_arenincola

-GCATAATAATTTGCCTCTTAATTAGAGGCTGGCATGAATGAACCCACGAGAGTCTAACTGTTTCA

TCCTTGCTAAAAACTATCCTTAAAATGAAGAGATTTTAATAAAATAAAAAGACAAAAAGACCCTAT

TGAGCTTTATCTAAAACCTTCCCCCTGCCTAAGATTTTGTTGGGGCGACATAAGAAGATTAGATCT

TCTTACAAAATAAATATGATCCGCCGCGATAAAGAAACCAGCTACCATAGGGATAACAGGCTAATC

TTCCTTAAGAGCCCGAATTGACAGGAAGGT--

--TTAAGCCATG

CAAGTCTAAGTACGAGCCCAGCACGGCGAAACCGCGGATGGCTCATTAAATCAGTTATGGTTCCTT

AGATCGTCCCGTCCTACTCGGATAACTGTAGTAATTCTAGAGCTAATACGTGCCACGAAGCTCCGA

CCTTCGGGGAAGAGCGCATTTATTAGACCAAAGCCAATCGGGGCGTCT--GTCATGGTGACTCTGG

ATAACTACAAGTCGATCGCACGGCCGCGCCGGCGACAGATCTATCAAATGTCTGCCCTATCAAGTC

TCGACGGTTGTTTAGCCGGCAACCGTGCTGATAACGGGTGACGGGGAATCAGGGTTCGATTCCGGA

GAGGGAGCATGAGAAACGGCTACCACTTCCAAGGAAGGCAGCAGGCACGCAAATTACCCACTCCCG

GCTCGGGGAGGTAGTGACGAAAAATAACAATACGGGACTCTTACGAGGCCCCGTAATTGGAATGAG

TACACTTTAAATCCTTTAACGAGGATCCATTGGAGGGCAAGTCTGGTGCCAGCAGCCGCGGTAATT

CCAGCTCCAATAGCGTATATTAAAGTTGTTGCAGTTAAAAAGCTCGTAGTTGGACGTGGGGAGCAG

CCCGGCGGTCCGCTCGACGGCGG-TCACTGCCTCCTGCTCCTCTTGCCGGCGGTGCGGGGTGCTCT

TGGTTGGTGCTGCCGGGACGTTTACTTTGAAAAAATTAGAGTGTTCAAAGCAGGCCCTGAATACTG

GTGCATGGAATAATGGAACAGGACCTCGCGTTTCGTTGGTTTTACGAAGTCGAGGTAATGATTAAT

AGGGACCGCGGGGGCATTTGTATTGCGCCGTTAGAGGTGAAATTCTTGGATCGGCGCAAGACAAAC

TAGAGCGAAGGCGTTTGCCAAGCACGTCTTCATTAATCAAGAGCGACAGTCAGAGGTTCGAAGACG

ATCAGATACCGTCGTAGTTCTGACCATAAACGATGCCAGCTAGGGACTCGCGGCGGTTGCTTGACC

CCGCGAGGACCTCCCCGGAAACGAAAGCTTTCGGGTTCCGGGGGAAGTATGGTTGCAAAGCTGAAA

CTTAAAGGAATTGACGGAAGGGCACCACCAGGAGTGGAGCCTGCGGCTTAATTTGACTCAACACGG

GGAAACTCACCCGGCCCGGACACCGTGAGGATTGACAGATTGAGAGCTCTTTCTTGATTCGGTGGG

TGGTGGTGCATGGCCGTTCTTAGTTGGTGGAGCGATTTGTCTGGTTGATTCCGATAACGAACGAGA

CTCTAGCCTATTGAATAGTCGGCCTGCTAGCGGGCGTGACTTCTTAGAGGGACGAGCGGCTTTCAG

CCGTACGAGATTGAGCAATAACAGGTCTGTGATGCCCTTAGATGTCCGGGGCCGCACGCGCGCTAC

ACTGAAGGACTCAGCGTGTG-TCTCCTGCTCAGAAATGAGTGGGCAACCCGTTGAACCTCCTTCGT

GCTAGGGATTGGGGATTGCAATTGTTCCCCATGAACGAGGAATTCCCAGTAAGCGCGAGTCATCAG

CTCGCGTTGATTACGTCCCTGCCCTTTGTACACACCGCCCGTCGCTACTACCGATCGGATGGTTTA

GTGAGACCCTCGGACTGGTCCCGACGGCAGTTCTCTGTCGTCGCGGTGCCGGGAAGACGGTCGAAC

TTGACTATCTAGAGGAAGTAA----TCACTAAGCGGAGGAAAAGAAACTAACAAGGATTCCCCTAG

TAACGGCGAGTGAAGCGGGAAGAGCCCAGCACCGAATCCCCTGCGCTCG-CGCAGAGGGAGCTGTG

GTGTTTGGGAGAGCCTCTGGTGCGGTGCCACGGTCCGAGTCTCCTTGATCGGGGTTGCCCATAGCG

GGTGTCAGGCCTCTACGACCGGCGGCGCCGTGCCTCTGCGCCTCCCAGGAGTCGGGTTGTTTGAGA
ATGCAGCCCAAAGCGGGTGGTAAACTCCATCTAAGGCTAAATACCGGCACGAGACCGATAGTAGAC
AAGTACCGTGAGGGAAAGTT--

>Rhynchospio_foliosa
--AAAGGT
AGCATAATAATTTGCCTCCTAATTAGAGGCTCGCATGAATGAATGCACGAGAGTCTAGCTGTTTCA
CTTCTAATAAAAACTATCCTTTAAGTGAAGAGACTTAAATAAAATAAAAAGACAAAAAGACCCTAT
TGAGCTTTACTTAAATGCCACACCTACCCACAAGTTTTGTTGGGGCGACAGAGGAAGACTTAATCT
TCCTTCTTCAAAAGAGCGATCCACTATGATAAAGAAACCAGCTACCATAGGGATAACAGGCTAATC
TTCTTTGAGAGCCCAAATTGACAAGAAGGTTTGGCACCTCGATGTTGGCTTAAGGTACCTGACAGA
TGCAGAAGTAAGTGGGTTTGTTCAACCTTTAATACCTT-----------------ATTAAGCCATG
CACGTCTAAGTACAAGCCTCAGAAGGCGAAACCGCGAACGGT---ATAAATCAGTTGTGGTTCTGT
TTGATG-GTGACCCTAGTTGGACAACTGTGGCAATTCTAGAGCTAATACATGAAATTAAGCTCCGA
CCTTCGGGGACGAGCGCTTTTATTAGACCAAAACCAACCGTGCCGGCGGAATTATGGTGACTCTGG
ATGA-CACATGTGGATCGCAAGGCTAAGCAGGCGACAGATCTTTCGAACGTCTGCCCTATCAAGTC
TCGACGGTTGTAGAGGGGCCAACCGTGCTGATAACGGGTGACGGGGAATCAGGGTTCGATTCCGGA
GAGGGAGCCTGAGAAACGGCTACCACTTCCAAGGAAGGCAGCAGGCGCGCAAATTACCCACTCCTA
GCCCAGGGAGGTAGTGACGAAAAATAACAATGCTGGTCTCTCACGAGGCCCAGTAATTGGAATGAG
TACACTTTAAAGACTTTAACCAGGACCTATTGGAGGGCAAGTCTGGTGCCAGCAGCCGCGGTAATT
CCAGCTCCAATGGCGTATACTAAAGTTGTTGCAGTTAAAAAGCTCGTAGTTGGATCTCGGGGAGCG
AGCGTCGGTCCTCTTCGCGGAGGCTTACTGGCTCACGCTCTCTGCGCAGTCCGGCCCGGGTGCCCT
TGATTGTCGCCGCTGCGACGTTTACTTTGAAAAAATTAGAGTGTTCAAAGCAGGCCCTGGATAGTC
ATGCATGGAATAATGGAAGAGGGCCTCGAAGTTTGTTGGCCTTGAGATGTCGAGGCAATGACTAAG
AGGGACGACGGGGACATTTATATTGCGCCAGGAGAGGTGAAATTCTTGGATTGGCGCAAGATAAAC
TGGAGCGAAGGCGTTTGTCAAGCACGTCTTCCTTAATCAAGAGCGACAGTCAGAGGCTCGAAGACG
ATCAGATACCGTCGTAGTTCTGACCGTAAACGCTGCCAGCTAGGGATTCGCTGTGGTTGCTAGACC
CTGCGAGGACCTTCCCGGAAACGAAAGCTTTCGGGTTCCGGGGGTAGTATGGTTGCAAAGCTGAAA
CTTAAAGGAATTGACGGAAGGGCACCACCAGGAGTGGAGCCTGCGGCTT-ATTTGACTCAACACGG
GGAATCTCACCCGGCCCGGACACCGTGAGGATTGACAGATTGAGAGCTCTTTCTTGATTCGGTGGG
TGGTGGTGCATGGCCGTTCTTAGTTGGTGGAGTGATTTGTCTGGTTAATTCCGATAACGAACGAGA

CTCTAGCCTATTAACTAGCAAGCCCCCTAAGGGGAGTGACTTCTTAGAGGGACAAGTGGCTTTCAG

TCACACGAGATTGAGCAATAACAGGTCTGTGATGCCCTTAGATGTCCGGGGCCGCACGCGTGCTAC

ACTGAAGGACTCAGCGTGTG--CTCCTTGCCTGAAAAGGCTGGGCAACCCGTTGAACCTCCTTCGT

GCTAGGGATTGGAGCTTGAAATTGTTCCCCATGAACGAGGAATTCCCAGTACGCGCCAGTCATCAG

CTGGCGTTGATTACGTCCCTGCCCTTTGTACACACCGCCCGTCGCTACTACCGATCGGATGGTTTA

GTGAGACCCTCGGACTGGTCCGTACGGCACCTCGGTGCTGTCTCGGCGCCGGGAAGACGGTCAAAC

TTGACTATCTAGAGGAAGTAAAAGTTCACTAAGCGGAGGAAAAGAAACTAACCAGGATTCCCCTAG

TAACGGCGAGTGAAGCGGGAAGAGCCCAGCACCGAATCCCCTGCTTTACACGTAGCGGGAACTGTG

GTGTTAGGGAGAGCCTCTGGTCAGGGAGTTGCGGCCAAGTCCTTGTGAAGAAGGGCTCCCCTGGAG

GGTGTCAGGCCCGTACGCTGACAGCTCCTTGACCTCTGCGCCTCCTTGGAGTCGGGTTGTTTGAGA

ATGCAGCCCTAAGCGGGTGGTAAACTCCATCTAAGGCTAAATATCAGCACGAGACCGATAGCAGAC

AAGTACCGTGAGGGAAAGTTGAAAAGAACTTTGA------------------------------

----

>Sabella_pavonina

AGCCTGTAGATTACAGGTATCCCCTGCCCGGTGTTAACGGCCACAGTATCCTGACTGTGCAAAGGT

AGCATAATAATTTGCCATTTAATTGGTGGCTAGTATGAATGGGTAAACAGAGATTTATTTTATTCT

TATT--CTGTAAATTAGTAATTATGTGAAAAACCATAAATTTACCTGAAAGACAAAAAGACCCTAC

TGAGCTTAAAGATTCCC----------CACCTCTTTTAGCTGGGACGACTAAAGAGCAAACTAAAC

CTCTTAGATTATATACTGCCCCTTAATGATCCGAATATAAGTTACCATAGGGATAACAGGCTAATT

CTCCTTAGGAGCCCAAATCGACAGGAGAGCTTGGCACCTCGATGTTGGCTTAGGGTTACTCTTTCC

TGCAGAAGGAAGTTGGTTTGTTCAACCATTAAACCCCT--------------------------

------TAAGTACATACTTAACACGGTGAAACTGCGAATGGCTCATTAAATCATATGTTATTTCTT

AGATCGTACAATCCTACTTGGATAACTGTGGTAATTCTAGAGCTAATACATGCATCAAAGCTCCGA

CCTCCGGGGAAGAGCGCTTTTATTAGACCAAAACCAATGGGGGCGTCC---CGTTGGTGACTCTGA

ATAACTTTAAGCTGATCGCATGGCCGTGCCGGCGACGTATCTTTCAAGTGTATGCCCTATCAATTG

TCGAAGGTAAGTGACTTGCTTACCTTGATGATAACGGGTAGCGGGGAATCAGGGTTCGATTCCGGA

GAGGGAGCATGAGAAACGGCTACCACATCCAAGGAAGGCAGCAGGCGCGCAAATTACCCACTCCTG

ACACGGGGAGGTAGTGACGAAAAATAACAATACGGGACTCTTTCGAGGCCCCGTAATTGGAATGAG

TACACTTTAAATCCTTTAACGAGGATCTATTGGAGGGCAAGTCTGGTGCCAGCAGCCGCGGTAATT

CCAGCTCCAATAGCGTATATTAAAGTTGTTGCAGTTAAAAAGCTCGTAGTTGGATCTCGGGTCCGG

ACTGGCGGTCCTATTCACGTAGGT--ACTGCTTGTTCCGACCCCCGCCGGTTTCCCTTGGTGCTCT

TCACTGGGGTGGCCGGAACGTTTACTTTGAAAAAATTAGAGTGTTCAAGGCAGGCCCTGAATATTG
TTGCATGGAATAATGGAATAGGACCTCGGATTTTGTTGGTTTTCGGGACTGGAGGTAATGATTAAG
AGGGACAGCGGGGGCATTAGTATTACGGTGTTAGAGGTGAAATTCTTGGATCGCCGTAAGACTAAC
TACTGCGAAAGCATTTGCCAAGAATGTTTTCATTATTCAAGAACGAAAGTCAGAGGTTCGAAGACG
ATCAGATACCGTCGTAGTTCTGACCATAAACGATGCCAGCTAGGGATTGGCGGGAGTTGTGTGACT
CCGCCAGCACCTTCCGGGAAACCAAAGCTTTTGGGTTCCGGGGGAAGTATGGTTGCAAAGCTGAAA
CTTAAAGGAATTGACGGAAGGGCACCACCAGGAGTGGAGCCTGCGGCTTAATTTGACTCAACACGG
GAAAACTCACCCGGCCCGGACACTGTTAGGATTGACAGATTGAGAGCTCTTTCTTGATTCGGTGGG
TGGTGGTGCATGGCCGTTCTTAGTTGGTGGAGCGATTTGTCTGGTTAATTCCGATAACGAACGAGA
CTCTAGTCTATTAAATAGTTCACGGTTTAGACCGTGTAACTTCTTAGAGGGACAAGTGGCGTTTAG
CCAACCGAGATTGAGCAATAACAGGTCTGTGATGCCCTTAGATGTTCGGGGCCGCACGCGCGCTAC
ACTGAAGGAATCAACGTGTA-TGTTCTGGCCCGGAAGGTTGGGTAACCCGCTTGAACCTCCTTCGT
GCTAGGGATTGGGGCTTGTAATTATTCCCCATGAACGAGGAATTCCCAGTAAGCGCGAGTCATAAG
CTCGCGTTGATTACGTCCCTGCCCTTTGTACACACCGCCCGTCGCTACTACCGATTGGATGATTTA
GTGAGGCCCTCGGACTGGTCCGGACGGGGGTCGCCCCTCTTTAGG-CGCCGGGAAGACGGCCAAAC
TTGATTATCTAGAGGAAGTAAAAGT----------------AAGAAACTAACAAGGATTCCCCCAG
TAACGGCGAGTGAAGCGGGAAGAGCCCAGCACCGAATCCCCCGCCTCTG-GGCGGTGTGAACTGTG
GTGTTTGGGAGAGCCTCTGGAGTGTTGGCTGCGTCCGAGTCCTCCTGATTGGGGTCACCCAGAGCG
GGTGTCAGGCCTCTACGGCCCCGGCCTCCACTTCTCTGAGCCTCCCCGGAGTCGGGTTGTTTGAGA
ATGCAGCCCAAAGTGGGTGGTAAACTCCATCTAAGGCTAAATACTGACACGAGTCCGATAGCGGAC
AAGTACCGTGAGGGAAAGTTGAAAAGAACTTTGAAGAGAGAGTTCAAGAGTACGTGAAACCGTTTA
GAGGCAAACGGATGGGACCGCAGTCTGCCCGGGGAATTCAATTCACCGAGGTCGGTGTCCGGATCC
T--TTGGGGACGCACTTTCCCCGGGAAGAGCGCCGCGACCGGTTCCCCGGCCGCCGAGCCCCGGAG
GAGGGTAGCTCGAGTGGTTTAGCCTCCGGAGG--TGGGCCGGGGGGACCGAGGAT-CCCCGCCGGG
AAGCGTCGGACCTGGGAGGACTGCTTGCAGTGCTCCCTAACCGCGTCTTCTCGTCTATCCAGGGTC
TGCGGCGAAGTCGGCACCCCATCCGACC

>Scolelepis_squamata
??
??
??
??
??
??
?????????????????????????????????????---------------------------
----

-TGCGGGCGCCCTTCGGGAAACCAAAGCTTTTGGGTTCCGGGGGAAGTATGGTTGCAAAGCTGAAA
CTTAAAGGAATTGACGGAAGGGCACCACCAGGAGTGGAGCCTGCGGCTTAATTTGACTCAACACGG
GGAAACTCACCCGGCCCGGACACCGTAAGGATTGACAGATTGAGAGCTCTTTCTTGATTCGGTGGG
TGGTGGTGCATGGCCGTTCTTAGTTGGTGGAGCGATTTGTCTGGTTAATTCCGATAACGAACGAGA
CTCTMGCCTATTAAATAGTTGGCCGATGGCTCAGCGAAACTTCTTAGAGGGACAAGTGGCTTTTAG
CCACGCGAGATTGAGCAATAACAGGTCTGTGATGCCCTTAGATGTTCGGGGCCGCACGCGCGCTAC
ACTGAAGGACTCAACGTGTCTCCCCCTATCCCGAAAGGGACGGGCAACCCGTTGAACCTCCTTCGT
GCTAGGGATTGGGGTTTGCAATTATCCCCCATGAACGAGGAATTCCCAGTAAGCGCGAGTCATCAG
CTCGCGTTGATTACGTCCCTGCCCTTTGTACACACCGCCCGTCGCTACTACCGATTGGATGGTTTA
GTGAGACCCTCGGATCGGTCCCGACGGTGGTTCTCTGCCGTCGTGGCGCC----------------
----
---CTACGCGGGAACTGTG
GTGTGTGGGAGAGCCTCTGGGACGTAGCGGACGTCCGAGTCCTCCTGATCGGGGTTACCCAGAGCG
GGTGTCAGGCCTCTACGGCGGCTGCTGCCGTCCCTCTGCGCCTCCTCGGAGTCGGGTTGTTTGAGA
ATGCAGCCCAAAGTGGGTGGTAAACTCCATCTAAGGCTAAATACTGGCACGAGGCCGATAGCGGAC
AAGTACCGTGAGGGAAAGTTGAAAAGAACTTTGAAGAGAGAGTTCAAGAGTACGTGAAACCGTATA
GAGGTAAACGGACGGGACCGCAGTCAAACCGTGGGATTCAGCTTTGTGCTGTGTAGTCGTAGACCC
GATCAGGGAGTGCACTTTCCGCGGGGAGAAAGCCACGACCGCTTCTTGGGCCGTCAAGCTCTTGGG
GAAGGTAGCTCGAGTGTTATAGCCCCTTGAAGTGCAGGCCGGGGGGAGCGAGGAATGCCCGCCGGT
CCTCGCAGCACTGGTGCGGACTGCTTGCAGTGCGTGCCGACCGCGCAGTCTCGGGGTTCTAGGGTC
TGTGGCGAAGTCGGCTTCCCGTCCGACC

>Scolelepis_texana

CGCCTCTAGATTAAAGGTCCATTCTGCTCTATGTAAATGGCCGCGGTACCCTGACCGTGCTAAGGT
AGCATAATAATTTGCCTCTTAATTAGAGGCTTGTATGAAGGAACATACGTGAGTATAACTGTTTCA
TTTTTACTAAAAATTATCCTTCAGGTGAAGAGACCTGAGTACCATAAAAAGACAAAAAGACCCTAT
TGAGCTTTACTTAAAGTATCCCTATTTTCCTAAGTTCTGTTGGGGCGACAAAAGAAGACTAAATCT
TCTTTCTTAATAAAATTGATCCATTTTGATATCGGAACCAGCTACCCTAGGGATAACAGGCTAATT
TTCTTTGAGAGCCCAAATTGACAAGAAAGATTGGCACCTCGATGTTGGCTTAAGGTTCCTTATAAA
TGCAGAAGTAAGTAGGTTTGTTCAACCTTTAAAACCTTATATGCTTGTCTCAAAGATTAAGCCATG
CATGTCTAAGTACAAGCCTAGCACGGTGAAACCGCGAATGGCTCATTAAATCAGTTATGGTTCCTT
AGATCGTACAAAAYTACTTGGATAACTGTAGTAATTCTAGAGCTAATACATGCTTCAAAGCTCCGA
CCTCCAGGGACGAGCGCTTTTATTAGACCAAAACCAGTYGGCCCCTGTCCCCTTTGGTGACTCTGG
ATAACTTTGTGCTGATCGCACGGCCGAGCCGGCGACGAATCTTTCAAATGTCTGCCCTATCAAGTT
TCGACGGTTGGTGATTTGCCAACCGTGCTGATAACGGGTAGCGGGGAATCAGGGTTCGATTCCGGA
GAGGGAGCATGAGAAACGGCTACCACTTCCAAGGAAGGCAGCAGGCGCGCAAATTACCCACTCCTA
GCTCGGGGAGGTAGTGACGAAAAATAACAATACGGGACTCTTACGAGGCCCCGTAATTGGAATGAG
TACACTTTAAATCCTTTAACGAGGATCTATTGGAGGGCAAGTCTGGTGCCAGCAGCCGCGGTAATT
CCAGCTCCAATAGCGTATATTAAAGTTGTTGCAGTTAAAAAGCTCGTAGTTGGATCTCGGGTGCAG
GCTGGCGGTCCGCCTCGCGGCGGTTTACTGCCTCCTGCTCCTCCTCCCGGTTCCCCGCAGTGCTCT
TTACTGGGGTGGCCGGAACGTTTACTTTGAAAAAATTAGAGTGTTCAAAGCAGGCCCTGAATACTG
TTGCATGGAATAATGGAATAGGACCTCGGATTTTGTTGGTTTTCGGAAGTCGAGGTAATGATTAAT
AGGGACGGCGGGGGCATTTGTATTGCGGCGTTAGAGGTGAAATTCTTGGATCGTCGCAAGACAAAC
TACAGCGAAGGCGTTTGCCAAGAATGTCTTCATTAATCAAGAGCGACAGTCAGAGGTTCGAAGACG
ATCAGATACCGTCGTAGTTCTGACCATAAACGATGCCAGCTGGGAATCCGCGGGAGTTGCCTGACT
CTGCGGGCGCCCTTCGGGAAACCAAAGCTTTTGGGTTCCGGGGGAAGTATGGTTGCAAAGCTGAAA
CTTAAAGGAATTGACGGAAGGGCACCACCAGGAGTGGAGCCTGCGGCTTAATTTGACTCAACACGG
GGAAACTCACCCGGCCCGGACACCGTAAGGATTGACAGATTGAGAGCTCTTTCTTGATTCGGTGGG
TGGTGGTGCATGGCCGTTCTTAGTTGGTGGAGCGATTTGTCTGGTTAATTCCGATAACGAACGAGA
CTCTAGCCTATTAAATAGTTGGCCGATGGCTATATGAAACTTCTTAGAGGGACAAGTGGCTTTTAG
CCACGCGAGATTGAGCAATAACAGGTCTGTGATGCCCTTAGATGTTCGGGGCCGCACGCGCGCTAC
ACTGAAGGACTCAACGTGTCTCCCCCTATCCCGAAAGGGATGGGCAACCCGTTGAACCTCCTTCGT
GCTAGGGATTGGGGTTTGCAATTATCCCCCATGAACGAGGAATTCCCAGTAAGCGCGAGTCATCAG
CTCGCGTTGATTACGTCCCTGCCCTTTGTACACACCGCCCGTCGCTACTACCGATTGGATGGTTTA
GTGAGACCCTCGGATCGGTCCCGACGGTGGTTCTCTGCCGTCGTGGCGCCGAGAAGACGGTCAAAC
TTGACTATCTAGAGGAAGTAAAAGT???
??
??

??
??
??
??
??
??
??
?????????????????????????????

>Spio_filicornis
----------TTAAAGGTCCTCCCTGCCCAATGTCAATGGCCGCGGTACCTTGACCGTGCTAAGGT
AGCATAATAATTTGTCTTTTAATTAAAGACTGGAATGAATGGAACTACGAGAGTTAAGCTGTTTCC
TTCCTAATAAAAATTAACCTGTAAGTGAAGAGGCTTACATAAAATAAAAAGACAAAAAGACCCTAT
TGAGCTTTACTACTCACGTTTTTCCTACACGTAGTTTTGTTGGGGCAACAAGAGAGGACTTTATCT
TCTCTAATACTATAACAGATCCGATGCGATATTAGAAAAAGCTACCATAGGGATAACAGGCTAATC
CTTCTTAAGAGATCAAATTGACAGAAGGGTTTGGCACCTCGATGTTGGCTTAGGGGACCATTTAGA
TGCAGAAGTTTGTGGGTTTGTTCAACCTTTAAAACCCTATATGCTTGTCTCAAAGACTAAGCCATG
CATGTCTAAGTATAAGCTCGACACGGCGAAACCGCGAATGGCTCATTAAATCAGTTATGGTTCCTT
AGATCGTACTATCCTACTTGGATAACTGTAGTAATTCTAGAGCTAATACATGCAACGAAGCTCCGA
CCCTCGGGGAAGAGCGCTTTTATTAGATCAAAACCAGAGGTCTCGGCCAACCTTTGGTGACTCTGG
ATAACTTC-AGCCGATCGCACGGCCGAGCCGGCGACGTATCTTTCAAATGTCTGCCCTATCAACTT
TCGACGGTTGGTGACTTGCCAACCGTGGTTATTACGGGTAGCGGGGAATCAGGGTTCGATTCCGGA
GAGGGAGCATGAGAAACGGCTACCACATCCAAGGAAGGCAGCAGGCGCGCAAATTACCCACTCCTA
GCTCGGGGAGGTAGTGACGAAAAATAACAATACGGGACTCTTACGAGGCCCCGTAATTGGAATGAG
TACACTTTAAATCCTTTAACGAGGATCTATTGGAGGGCAAGTCTGGTGCCAGCAGCCGCGGTAATT
CCAGCTCCAATAGCGTATATTAAAGTTGTTGCAGTTAAAAAGCTCGTAGTTGGATCTCGGGAGAGG
GCCGGCGGTCCGCCTCGCGGCGGTTCACTGCCCCCTCCTCCCCTCGCCGGTCCTCCCCGGTGCCCT
TCGTTGGGTGGGCCGGAGCGTTTACTTTGAAAAAATTAGAGTGTTCAAAGCAGGCCTTGTATACCG
TTGCATGGAATAATGGAATAGGACCTCGGATTTTGTTGGTTTTCGGAACGCGAGGTAATGATTAAT
AGGGACCGCGGGGGCATTTGTATTGCGCGGTTAGAGGTGAAATTCTTGGATCCGTGCAAGACAGAC
TACAGCGAAGGCGTCTGCCAAGCACGTCTTCTTTAATCAAGAGCGACAGTCAGAGGTTCGAAGACG
ATCAGATACCGTCGTAGTTCTGACCATAAACTATGCCAGCTAGGGATTCGCGGGCGTTGTACGACT
CGGCGAGGACCTTCCCGGAAACGAAAGCTTTTGGGTTCCGGGGGAAGTATGGTTGCAAAGCTGAAA
CTTAAAGGAATTGACGGAAGGGCACCACCAGGAGTGGAGCCTGCGGCTTAATTTGACTCAACACGG
GGAAACTCACCCGGCCCGGACACCGTAAGGATTGACAGATTGAGAGCTCTTTCTTGATTCGGTGGG
TGGTGGTGCATGGCCGTTCTTAGTTGGTGGAGCGATTTGTCTGGTTAATTCCGATAACGAACGAGA

CTCTAGCCTATTAAATAGTTCGCCGGTTAGCCGGCGCAACTTCTTAGAGGGACAGGTGGCGTTCAG
CCACGCGAGATTGAGCAATAACAGGTCTGTGATGCCCTTAGATGTCCGGGGCCGCACGCGCGCTAC
ACTGAAGGACTCAGCGTGT--TCTCCTGGCCAGAAATGGTCGGGCAACCCGTTGAACCTCCTTCGT
GCTAGGGATTGGGGGTTGCAATTGTTCCCCATGAACGAGGAATTCCCAGTAAGCGCGAGTCATCAG
CTCGCGTTGATTACGTCCCTGCCCTTTGTACACACCGCCCGTCGCTACTACCGATCGGATGGTTTA
GTGAGACACTCGGACTGGTCCCGACGGCGGGTGACCGCCTTCGTGTTGCCGGGAAGAATGTCAAAC
TTGATTATCTAGAGGAAGTAAAAGT???
??
??
??
??
??
??
??
??
??
????????????????????????????

>Spio_sp_2573

-GCATAATAATTTGTCTTTTAATTAAAGACTGGAATGAATGGAACTACGAGAGTTAAGCTGTTTCC
CCCTTAATAAAAATTAACCTGTAAGTGAAGAGGCTTATATATAATAAAAAGACAAAAAGACCCTAT
TGAGCTTTACTACACCTGTTTTCCTTGTTAGTAGTTTTGTTGGGGCAACAAGAGAGGACTTTATCT
TCTCTAATATCTTAACAGATCCGATGCGATATTAGAAAAAGCTACCATAGGGATAACAGGCTAATC
CTTCTTAAGAGATCAAATTGACAGAAGGGT------------------------------------
----
--TAAATCAGTTATGGTTCCTT
AGATCGTACTATCCTACTTGGATAACTGTAGTAATTCTAGAGCTAATACATGCAACGAAGCTCCGA
CCCTCGGGGAAGAGCGCTTTTATTAGACCAAAACCAGAGGTCTCGGCCAACCTTTGGTGACTCTGG
ATAACTTC-AGCCGATCGCACGGCCGAGCCGGCGACGTATCTTTCAAATGTCTNCCCTATCAACTT
TCGACGGTTGGTGACTTGCCAACCGTGGTTATTACGGGTAGCGGGGAATCAGGGTTCGATTCCGGA
GAGGGAGCATGAGAAACGGCTACCACATCCAAGGAAGGCAGCAGGCGCGCAAATTACCCACTCCTA
GCTCGGGGAGGTAGTGACGAAAAATAACAATACGGGACTCTTACGAGGCCCCGTAATTGGAATGAG
TACACTTTAAATCCTTTAACGAGGATCTATTGGAGGGCAAGTCTGGTGCCAGCAGCCGCGGTAATT
CCAGCTCCAATAGCGTATATTAAAGTTGTTGCAGTTAAAAAGCTCGTAGTTGGATCTCGGGAGAGG
GCCGGCGGTCCGCCTCGCGGCGGTTCACTGCCCCCTCCTCCCCTCGCCGGTCCTCCCCGGTGCCCT

TCGTTGGGTGGGCCGGAGCGTTTACTTTGAAAAAATTAGAGTGTTCAAAGCAGGCCTTGTATACCG

TTGCATGGAATAATGGAATAGGACCTCGGATTTTGTTGGTTTTCGGAACGCGAGGTAATGATTAAT

AGGGACCGCGGGGGCATTTGTATTGCGCGGTTAGAGGTGAAATTCTTGGATCCGTGCAAGACAGAC

TACAGCGAAGGCGTCTGCCAAGCACGTCTTCTTTAATCAAGAGCGACAGTCAGAGGTTCGAAGACG

ATCAGATACCGTCGTAGTTCTGACCATAAACTATGCCAGCTAGGGATTCGCGGGCGTTGTACGACT

CGGCGAGGACCTTCCCGGAAACGAAAGCTTTTGGGTTCCGGGGGAAGTATGGTTGCAAAGCTGAAA

CTTAAAGGAATTGACGGAAGGGCACCACCAGGAGTGGAGCCTGCGGCTTAATTTGACTCAACACGG

GGAAACTCACCCGGCCCGGACACCGTAAGGATTGACAGATTGAGAGCTCTTTCTTGATTCGGTGGG

TGGTGGTGCATGGCCGTTCTTAGTTGGTGGAGCGATTTGTCTGGTTAATTCCGATAACGAACGAGA

CTCTAGCCTATTAAATAGTTCGCCGGTTAGCCGGCGCAACTTCTTAGAGGGACAGGTGGCGTTCAG

CCACGCGAGATTGAGCAATAACAGGTCTGTGATGCCCTTAGATGTCCGGGGCCGCACGCGCGCTAC

ACTGAAGGACTCAGCGTGT--TCTCCTGGCCAGAAATGGTCGGGCAACCCGTTGAACCTCCTTCGT

GCTAGGGATTGGGGGTTGCAATTGTTCCCCATGAACGAGGAATTCCCAGTAAGCGCGAGTCATCAG

CTCGCGTTGATTACGTCCCTGCCCTTTGTACACACCGCCCGTCGCTACTACCGATCGGATGGTTTA

GTGAGACACTCGGACTGGTCCCGACGGCGGGTGACCGCCTTCGTGTTGCCGGGAAGAATGTCAAAC

TTGATTATCTAGAGGAAGTAAAAGTTCACTAAGCGGAGGAAAAGAAACTAACCAGGATTCCCCTAG

TAACGGCGAGTGAAACGGGAAGAGCCCAGCACCGAATCCCCTGCATCGACTGTAGAGGGAACTGTG

GTGTTAAGGAGAGCCTGTGGAGCGGGAG-GGCGTCCGAGTCCTCCTGATTGGGGTTGCCCAGAGCG

GGTGTCAGGCCTCTACGGCGCCCCGCCCCGTTGCTCAGTACCTCCTAGGAGTCGGGTTGTTTGAGA

TTGCAGCCCAAAGTGGGTGGTAAACTCCATCTAAGGCTAAATACTGGCACGAGACCGATAGCAAAC

AAGTACCGTGAGGGAAAGTT---

>Spiophanes_cf_convexus

CGCCTCTAGATTAGAGGTTCATCCTGCCCTATGTAAATGGCCGCGGTACTTTGACCGTGTTAAGGT

AGCATAATAATTTGCCTTTTAATTGAAGGCTTGTATGAATGGATAAACGAGAATAAAGCTGTCTCA

ATTTTAATAAAAATTAATATTCAAATGAAGAAGTTTGAATAAAATAAAAAGACAAGAAGACCCTGT

TGAGCTTTATCTCAGATATTTTCTTGAAATGAGATTTAATTGGGGCGATTAAGGAAGAATAAAATC

TTCCTAAAAGTTAAATGTATCCATAGTGA-TAAATAATTAGCTACCACAGGGATAACAGGCTAATT

TCTTTTAAGAGTTCATATCGAAAAAGAAGTTTGGCACCTCGATGTTGGCTTAGGATAAGAATTAAA

TGCAGAAGTGACTGGGTTTGTTCAACCCTTAAATTCCT-------------AAGATTAAGCCATG

CATGTCTAAGTACAAGCTTAACACGGTGAAACCGCGAATGGCTCATTAAATCAGTTATGGTTCCTT

AGATCGTACAATCCTACTTGGATAACTGTAGTAATTCTAGAGCTAATACATGCAAGCAAGCTCCGA
CCTTCGGGGAAGAGCGCTTTTATTAGACCAAAACCAAT--GGGCGACCTTCCTTTGGTGACTCTGG
ATAACTTT--GCTGATCGAATGGCCGTGCCGTCGATGTATCTTTCAAATGTCTGCCCTATCAAGTT
TCGTAGGTAGTCGATATGACTACCTAGCTTGTAACGGGTAACGGGGAATCAGGGTTCGATTCCGGA
GAGGGAGCATGAGAAACGGCTACCACATCCAAGGAAGGCAGCAGGCGCGCAAATTACCCACTCCTG
GCACGGGGAGGTAGTGACGAAAAATAACAATACGGGACTCTTTCGAGGCCCCGTAATTGGAATGAG
TACACTTTAAATCCTTTAACGAGGATCTATTGGAGGGCAAGTCTGGTGCCAGCAGCCGCGGTAATT
CCAGCTCCAATAGCGTATATTAAAGTTGTTGCAGTTAAAAAGCTCGTAGTTGGATCTCGGGAGCAG
GCTGGCGGTCCGCCTCGCGGCGG-TTACTGCCCGTCCTGCCCCCTGCCGTCTTCCCTTGGTACTCT
TCATTGGGGTGGACGGAACGTTTACTTTGAAAAAATTAGAGTGTTCAAAGCAGGCCCTGAATAATG
GTGCATGGAATAATGGAATAGGACCTCGGATTTTGTTGGTTTTCGGAACTTGAGGTAATGATTAAG
AGGGACAGCGGGGGCATTAGTATTACGGTGTTAGAGGTGAAATTCTTGGATCGCCGTAAGACTAAC
TACTGCGAAAGCATTTGCCAAGCATGTTTTCTTTAATCAAGAACGAAAGTCAGAGGTTCGAAGACG
ATCAGATACCGTCGTAGTTCTGACCATAAACGATGCCAGCTAGGGATTCGCCGGCGTTGTATGACT
CGGCGAGCACCTTCCGGGAAACCAAAGCTTTTGGGTTCCGGGGGAAGTATGGTTGCAAAGCTGAAA
CTTAAAGGAATTGACGGAAGGGCACCACCAGGAGTGGAGCCTGCGGCTTAATTTGACTCAACACGG
GAAAACTCACCCGGCCCGGACACTGTAAGGATTGACAGATTGAGAGCTCTTTCTTGATTCGGTGGG
TGGTGGTGCATGGCCGTTCTTAGTTGGTGGAGCGATTTGTCTGGTTAATTCCGATAACGAACGAGA
CTCTAGCCTATTAAATAGTTCGCCGATTCGTCGGCGCAACTTCTTAGAGGGACAAGTGGCGTTTAG
CCACGCGAGATTGAGCAATAACAGGTCTGTGATGCCCTTAGATGTTCGGGGCCGCACGCGCGCTAC
ACTGAAGGGATCAACGTGTGTTCTCCTGGCCCGGAAGGGTCGGGTAACCCCTTGAACCCCCTTCGT
GCTTGGGATAGGGGCTTGCAATTACCCCCT-TGAACGAGGAATTCCCAGTAAGCGCGTGTCATCAG
CACGCGTTGATTACGTCCCTGCCCTTTGTACACACCGCCCGTCGCTACTACCGATTGGATGGTTTA
GTGAGACCCTCGGATTGGTCCCGGCGGTGGGCGACCGCTGCCGTGGCGCCG-AAAGACGGTCAAAC
TTGACTATCTAGAGGAAGTAAAAGTTCACTAAGCGGAGGAAAAGAAACTAACAAGGATTCCCCTAG
TAACGGCGAGTGAAGCGGGAAGAGCCCAGCACCGAATCCCCTGCATGAACTGTAGCGGGAACTGTG
GTGTTTGGGAGAGCCTCTGGAGCGCCGCCGGCGTCCGAGTCCTCCTGATCGGGGCTACCCATAGCG
GGTGTCAGGCCTCTACGGCCTCCGGCGTCGTTCCTCTGAGCCTCCCTGGAGTCGGGTTGTTTGGGA
ATGCAGCCCTAAGTGGGTGGTAAACTCCATCTAAGGCTAAATACTGGCACGAGTCCGATAGCGGAC
AAGTACCGTGAGGGAAAGTT--

>Spiophanes_uschakowi

-GCATAATAATTTGCTTTTTAATTGAAGGCTTGTATGAATGGATAAACGAGAATAAAGCTGTCTCA

ATTTTAGTATAAATTAATATTCAAATGAAGAAGTTTGAATAATATAAAAAGACAAGAAGACCCTGT

TGAGCTTTATTTATTATATTATTTTAAATATAAATTTAATTGGGGTGATTAAGGAAGAAAAAAATC

TTCCTAGAAATTTAGTGTATTCAAAATGA-TAAATAATAAGCTACCACAGGGATAACAGGCTAATT

TCTTTTGAGAGTTCATATCGAAAAAGAAGT------------------------------------

---AAAGATTAAGCCATG

CATGTCTAAGTACAAGCTTCACAAGGTGAAACCGCGAATGGCTCATTAAATCAGTTATGGTTCCTT

AGATCGTACAATCCTACTTGGATAACTGTAGTAATTCTAGAGCTAATACATGCAATCAAGCTCCTA

CCTTCGGGGAAGAGCGCTTTTATTAGACCAAAACCAAT--GGGCAACCGTCCTTTGGTGACTCTGG

ATAACTTC--GCTGATCGTATGGCCGCGCCGTCGATGTATCTTTCAAATGTCTGCCCTATCAAGTC

TCGTAGGTAGTCGACATGACTACCTAGCTGATTACGGGTAACGGGGAATCAGGGTTCGATTCCGGA

GAGGGAGCATGAGAAACGGCTACCACATCCAAGGAAGGCAGCAGGCGCGCAAATTACCCACTCCTG

GCACGGGGAGGTAGTGACGAAAAATAACAATACGGGACTCTTTCGAGGCCTCGTAATTGGAATGAG

TACACTTTAAATCCTTTAGCAAGGATCTATTGGAGGGCAAGTCTGGTGCCAGCAGCCGCGGTAATT

CCAGCTCCAATAGCGTATATTAAAGTTGTTGCAGTTAAAAAGCTCGTAGTTGGATCTCGGGAGCAG

GCTGGCGGTCCGCCTCGCGGCGG-CTACTGCCTGTCCTGCCCCCTGCCGTCATCCCAGGGTACTCT

TCGTTGGGCTGGGCGGTTCGTTTACTTTGAAAAAATTAGAGTGTTCAAAGCAGGCCCTGAATAATG

GTGCATGGAATAATGGAATAGGACCTCGGATTTTTGTTGGTTAAGGAACTGGAGGTAATGATTAAG

AGGGACTGCGGGGGCATTAGTATTACGGTGTTAGAGGTGAAATTCAAGGATCATCGTAAGACTAAC

TACAGCGAAAGCATTTGCCAAGAACGTTTTCTTTAATCAAGAACGAAAGTCAGAGGTTCGAAGACG

ATCAGATACCGTCGTAGTTCTGACCATAAACAATGCCAGCTAGGGATCCGCCTGTGTTGTACGACC

GGGCGAGGACCTTCCGGGAAACCAAAGCTTTTGGGTTCCGGGGGAAGTATGGTTGCAAAGCTGAAA

CTTAAAGGAATTGACGGAAGGGCACCACCAGGAGTGGAGCCTGCGGCTTAATTTGACTCAACACGG

GAAAACTCACCCGGCCCGGACACTGTAAGGATTGACAGATTGAGAGCTCTTTCTTGATTCGGTGGG

TGGTGGTGCATGGCCGTTCTTAGTTGGTGGAGCGATTTGTCTGGTTAATTCCGATAACGAACGAGA

CTCTAGCCTATTAAATAGTTCGCCGATTCGTCGGCGCAACTTCTTAGAGGGACAAGTGGCGTTTAG

CCACGCGAGATTGAGCAATAACAGGTCTGTGATGCCCTTAGATGTTCGGGGCCGCACGCGCGCTAC

ACTGAAGGGATCAGCGTGTGTTCTCCTGGCCCGGAAGGGTCGGGTAACCCGTTGAACCCCCTTCGT

GCTTGGGATAGGGGCTTGCAATTACCCCCT-TGAACGAGGAATTCCCAGTAAGCGCGTGTCATCAG

CACGCGTTGATTACGTCCCTGCCCTTTGTACACACCGCCCGTCGCTACTACCGATTGGATGGTTTA

GTGAGACCCTCGGATTGGTCCCGACGGCGGGCAACCGCCGTCGTGGCGCCGAAAAGACGGTCAAAC

TTGACTATCTAGAGGAAGTAAAAGTTCACTAAGCGGAGGAAAAGAAACTAACAAGGATTCCCCTAG

TAACGGCGAGTGAAGAGGGAAGAGCCCAGCACCGAATCCCCTGCATTGACTGTAGTGGGAACTGTG

GTGTTTGGGAGAGCCTCTGGAGTTGTGCCTGCGTCCGAGTCCTCCTGATCGGGGTTGCCCATAGCG

GGTGTCAGGCCTCTAGGACCTCGGGC-CCGCTTCTCTGAGCCTCCTAGGAGTCGGGTTGTTTGGGA
ATGCAGCCCTAAGTGGGTGGTAAACTCCATCTAAGGCTAAATACTGGCACGAGTCCGATAGCGAAC
AAGTACCGTGAGGGAAAGTT--

>Streblospio_sp
CGCCTCCAGATTGGAGGTCCATCCTGCCCAGTGTAAACGGCCGCGGTATCCTGACCGTGCTAAGGT
AGCATAATCATTTGCCTTTTAATTGAAGGCTAGTATAAACGGACAAACGAAAAAATATCTGTCTCA
ACAATAAAAAAAATTGATCATTAAGTGAAGAAACTTAAATTTTTCAAAAAGACGAAAAGACCCTAT
GGCGCTTTACTTTATCT--AATTATTAAGTACAGTTTAGTTGGGGCGACTAAGGAGAAAACAAAAC
CTCTTTATACAAAAATTTATCCATTATGA-TAAGAAATAAGTAACCCTAGGGATAACAGGCTAATC
TTTCTTGAGAGTTCACATTAACAGAAAGGGTTGGCACCTCGATGTTGGCTTAAAGTTCCTTTAAGG
TGCAGCAACAAGTGGGTTTGTTCAACCCTTAAAACTTTATATGCTTGTCTCAAAGACTAAGCCATG
CATGTCTAAGTACAAGCTCAACATGGTGAAACCGCGAATGGCTCATTAAATCAGTTATGGTTCCTT
AGATCGTACAATCCTACTTGGATAACTGTAGTAATTCTAGAGCTAATACATGCGAGCAAGCTCCGA
CCCTCGGGAAAGAGCGCTTTTATTAGACCAAAACCAAT--TGGCAACATCCTTTTGGTGACTCTGG
ATAACTTTGAGCAGATCGCACGGCCGCGCCGGCGACGTATCTTTCAAATGTCTGCCCTATCAACTT
TCGACGGTTAGTTATACGCTCACCGTGGTCGTAACGGGTAGCGGGGAATCAGGGTTCGATTCCGGA
GAGGGAGCATGAGAAACGGCTACCACATCCAAGGAAGGCAGCAGGCGCGCAAATTACCCACTCCCG
GCACGGGGAGGTAGTGACGAAAAATAACAATACGGGACTCTTTCGAGGCCCCGTAATTGGAATGAG
TACACTTTAAATCCTTTAACGAGGATCTATTGGAGGGCAAGTCTGGTGCCAGCAGCCGCGGTAATT
CC--

-------------------------TCACTAAGCGGAGGAAAAGAAACTAACAAGGATTCCCCTAG
TAACGGCGAGTGAAGCGGGAAGAGCCCAGCACCGAATCCCCGGCGGTCTCCGTCGCGGGAACTGTG
GTGTTTGGGAGCGCCAATGGGGCGCCGCCGGCGTCCGAGTCCTCCTGATCGGGGTCACCCAGAGCG
GGTGTCAGGCCTCTACGGCCTCCGGCGTCGTCCCTCTGAGCCTCCCCGGAGTCGGGTTGTTTGGGA
ATGCAGCCCAAAGTGGGTGGTAAACTCCATCTAAGGCTAAATACCGGCGTGAGTCCGATAGCGGAC
AAGTACCGTGAGGGAAAGTTGAAAAGAACTTTGAAGAGAGAGTTCA--------------------
----

>Trochochaeta_multisetosa

-GCATAATCATTTGCCTYTTAATTAGAGGCTGGAATGAATGGATAAACGAAATTACAACTGTCTCC
TCTCTGATAAAAATTAAGCTTCAAGTTAAAACGCTTGGATAAGATAAAAGGACAAAAAGACCCTAT
TGAGCTTTATTTTACTTCCCAATTGTAAATAAAATTTAGTTGGGGCGACGAAGGAAAAAATAAATC
TTCCTTATAACAAACATGATCCAAACTGATAAAAGAAAAAGCTACCATAGGGATAACAGACTAATC
TTTTTTAAGAGACCATATTGACAAAAAGGA------------------------------------
----
------TAAGTACAAGCTTAACACGGTGAAACCGCGAATGGCTCATTAAATCAGTTATGGTTCCTT
AGATCGTACAATCCTACTTGGATAACTGTAGTAATTCTAGAGCTAATACATGCAAGCAAGCTCCGA
CCTTCGGGGAAGAGCGCTTTTATTAGACCAAAACCAAT--GGGCAACCTTGATCTGGTGACTCTGG
ATAACTTTGAGCTGATCGCATGGCCGAGCCGGCGACGTATCTTTCAAATGTCTGCCCTATCAACTT
TCGTAGGTAGTTGATATGACTACCTAGGTTGTAACGGGTAACGGGGAATCAGGGTTCGATTCCGGA
GAGGGAGCATGAGAAACGGCTACCACATCCAAGGAAGGCAGCAGGCGCGCAAATTACCCACTCCTG
GCACGGGGAGGTAGTGACGAAAAATAACAATACGGGACTCTTTTGAGGCCCCGTAATTGGAATGAG
TACACTTTAAATCCTTTAACGAGGATCTATTGGAGGGCAAGTCTGGTGCCAGCAGCCGCGGTAATT
CCAGCTCCAATAGCGTATATTAAAGTTGTTGCAGTTAAAAAGCTCGTAGTTGGATCTCGGGTGCAG
GCTGGCGGTCCGCCTCGCGGCGG-TTACTGCCTGTCCTGCCCCCAGCCGGTCTCCCTTGGTGCTCT

TCACTGGGGTGGCCGGAACGTTTACTTTGAAAAAATTAGAGTGTTCAAAGCAGGCCCTGGATAATA

GTGCATGGAATAATGGAATAGGACCTCGGATTTTGTTGGTTTTCGGAGCTCGAGGTAATGATTAAG

AGGGACGGCGGGGGCATTTGTATTACGGTGTTAGAGGTGAAATTCTTGGATCGCCGTAAGACAAAC

TACTGCGAAAGCATTTGCCAAGCATGTTTTCATTAATCAAGAACGAAAGTCAGAGGTTCGAAGACG

ATCAGATACCGTCGTAGTTCTGACCATAAACGATGCCAGCTAGGGATTCGTCGGAGTTGTATGACT

CGACGAGCACCTTCCGGGAAACCAAAGCTTTTGGGTTCCGGGGGAAGTATGGTTGCAAAGCTGAAA

CTTAAAGGAATTGACGGAAGGGCACCACCAGGAGTGGAGCCTGCGGCTTAATTTGACTCAACACGG

GGAAACTCACCCGGCCCGGACACTGTAAGGATTGACAGATTGAGAGCTCTTTCTTGATTCGGTGGG

TGGTGGTGCATGGCCGTTCTTAGTTGGTGGAGCGATTTGTCTGGTTAATTCCGATAACGAACGAGA

CTCTAGCCTACTAAATAGTTCGCAGATTTGTCTGCGCAACTTCTTAGAGGGACAAGTGGCGTTTAG

CCACGCGAGATTGAGCAATAACAGGTCTGTGATGCCCTTAGATGTTCGGGGCCGCACGCGCGCTAC

ACTGAAGGAATCAGCGTGTGTTCTCCTGGCCAGAAATGGTTGGGTAACCCGTTGAACCTCCTTCGT

GCTAGGGATTGGGGCTTGCAATTCTTCCCCATGAACGAGGAATTCCCAGTAAGCGCGAGTCATCAG

CTCGCGTTGATTACGTCCCTGCCCTTTGTACACACCGCCCGTCGCTACTACCGATTGGATGGTTTA

GTGAGACCTTCGGATTGGTCCCGACGGTGGGCAACCACCGTCGAGGTGCCGAGAAGATGTTCAAAC

TTGACTATCTAGGAAGTAAAAGT???

??

??

??

??

??

??

??

??

??

????????????????????????????

>Trochochaeta_sp_THS_2006

??

??

??

??

??

??

??????????????????????????????????????ATATGCTTGTCTCAAAGACTAAGCCATG

CATGTCTAAGTACAAGCTTAACACGGTGAAACCGCGAATGGCTCATTAAATCAGTTATGGTTCCTT

AGATCGTACAATCCTACTTGGATAACTGTAGTAATTCTAGAGCTAATACATGCAAGCAAGCTCCGA
CCTTCGGGGAAGAGCGCTTTTATTAGACCAAAACCAAT--GGGCAACCTTGATCTGGTGACTCTGG
ATAACTTTGAGCTGATCGCATGGCCGAGCCGGCGACGTATCTTTCAAATGTCTGCCCTATCAACTT
TYGTAGGTAGTTGATATGACTACCTAGGTTGTAACGGGTAACGGGGAATCAGGGTTCGATTCCGGA
GAGGGAGCATGAGAAACGGCTACCACATCCAAGGAAGGCAGCAGGCGCGCAAATTACCCACTCCTG
GCACGGGGAGGTAGTGACGAAAAATAACAATACGGGACTCTTTTGAGGCCCCGTAATTGGAATGAG
TACACTTTAAATCCTTTAACGAGGATCTATTGGAGGGCAAGTCTGGTGCCAGCAGCCGCGGTAATT
CCAGCTCCAATAGCGTATATTAAAGTTGTTGCAGTTAAAAAGCTCGTAGTTGGATCTCGGGTGCAG
GCTGGCGGTCCGCCTCGCGGCGG-TTACTGCCTGTCCTGCCCCCAGCCGGTCTCCCTTGGTGCTCT
TCACTGGGGTGGCCGGAACGTTTACTTTGAAAAAATTAGAGTGTTCAAAGCAGGCCCTGGATAATA
GTGCATGGAATAATGGAATAGGACCTCGGATTTTGTTGGTTTTCGGAGCTCGAGGTAATGATTAAG
AGGGACGGCGGGGGCATTTGTATTACGGTGTTAGAGGTGAAATTCTTGGATCGCCGTAAGACAAAC
TACTGCGAAAGCATTTGCCAAGCATGTTTTCATTAATCAAGAACGAAAGTCAGAGGTTYGAAGACG
ATCAGATACCGTCGTAGTTCTGACCATAAACGATGCCAGCTAGGGATTCGTCGGAGTTGTATGACT
CGACGAGCACCTTCCGGGAAACCAAAGCTTTTGGGTTCCGGGGGAAGTATGGTTGCAAAGCTGAAA
CTTAAAGGAATTGACGGAAGGGCACCACCAGGAGTGGAGCCTGCGGCTTAATTTGACTCAACACGG
GGAAACTCACCCGGCCCGGACACTGTAAGGATTGACAGATTGAGAGCTCTTTCTTGATTCGGTGGG
TGGTGGTGCATGGCCGTTCTTAGTTGGTGGAGCGATTTGTCTGGTTAATTCCGATAACGAACGAGA
CTCTAGCCTACTAAATAGTTCGCAGATTTGTCTGCGCAACTTCTTAGAGGGACAAGTGGCGTTTAG
CCACGCGAGATTGAGCAATAACAGGTCTGTGATGCCCTTAGATGTTCGGGGCCGCACGCGCGCTAC
ACTGAAGGAATCAGCGTGTGTTCTCCTGGCCAGAAATGGTTGGGTAACCCGTTGAACCTCCTTCGT
GCTAGGGATTGGGGCTTGCAATTCTTCCCCATGAACGAGGAATTCCCAGTAAGCGCGAGTCATCAG
CTCGCGTTGATTACGTCCCTGCCCTTTGTACACACCGCCCGTCGCTACTACCGATTGGATGGTTTA
GTGAGACCTTCGGATTGGTCCCGACGGTGGGCAACCACCGTCGAGGTGCCGAGAAGATGTTCAAAC
TTGACTATCTAGAGGAAGTAAAAGTTCACTAAGCGGAGGAAAAGAAACTAACAAGGATTCCCCTAG
TAACGGCGAGTGAAGCGGGAAGAGCCCAGCACCGAATCCCCTGCGTTTG-CGTAGCGGGACCTGTG
GTGTTTGGGAGAGCCTCTGAGGCGTCGACGGCGTCCGAGTCCTCCTGATCGGGGTCACCCAGAGCG
GGTGTAAGGCCCATACGACTTCCGTCTTCGTCTCTCTGTGCCTCCCTGGAGTCGGGTTGTTTGTGA
ATGCAGCCCAAAGTGGGTGGTAAACTCCATCTAAGGCTAAATACTGGCACGAGTCCGATAGCGGAC
AAGTACCGTGAGGGAAAGTTGAAAAGAACTTTGAAGAGAGAGTTCAAGAGTACGTGAAACCGCATA
GAGGCAAACGGATGGGACCGCAGTCGACCTTCAGAATTCATCTGCCGAGGGGCTGGCGTCTACTCT
TCATCGACAGTGCACTTTCTGGAGGTGGAGCGCTACGACCGGTTTCTGGGCCGTCAAGCCCCGGGG
AAAGGTAGCTCGAGTGTTATAGTCCCCGGTG-TGTCGGCCGGGGAGACCGAGGAACACCCGCCTGA
GCCCTGCGCACCTGGAAGGACTGCTTGCAGTGCTTACTAACCGCGTTGTAGGGGCCAGCCAGGGTC
TGTAGCGAAGTCGGT-TTCCATCCGACC

>Boccardiella_hamata

CGCCTCTAGATTAGAGGTCCATTCTGCCCTATGTAAATGGCCGCGGTAGCCTGACCGTGCTAAGGT
AGCATAATCATTTGCCGTTTAATTGGTGGCTGGAATGAACGAATATACGAGAGTTTAGCTGTCTCC
TTTTTAATAAAAATTGACTTTCAGGTGAAGAGACCTGAATATTTAAAAAAGACGAAAAGACCCTAT
AGAGCTTCACCTTTACCACTTTACTAAATAAAGGTTTAGTTGGGGCAACTAGAGAGAACTAAAACC
TCTCTTTAATAAAATTTGATCCGATGCGATAACAGAAAAAGCTACCTTAGGGATAACAGGCTAATC
TTCTTTGAGAGACCATATTGACAAGAAGGATTGGCACCTCGATGTTGGCTCAAGGTTACAGTTTGA
TGCAGCAGTAAGTAGGTTTGTKCACCCTTTAAATCCTTATATGCTTGTCTCAAAGACTAAGCCATG
CATGTCTAAGTATAAGCTCTATATGGTGAAACCGCGAATGGCTCATTAAATCAGTTATGGTTCCTT
AGATCTTAAAATCCTACTTGGATAACTGTAGTAATTCTAGAGCTAATACATGCACCTGAGCTCCAA
CCCTCGGGGAAGAGCGCTTTTATTAGACCAAAACCAATGGTCCGTGCCAACCTTTGGTGACTCTGG
ATAACTTC-AGCTGATCGCACGGCCGCGCCGGCGACGTATCTTTCAAGTGTCTGCCCTATCAAGTT
TCGATGGTTGGTGACTTGCCAACCATGCTCGTAACGGGTAGCGGGGAATCAGGGTTCGATTCCGGA
GAGGGAGCATGAGAAACGGCTACCACATCCAAGGAAGGCAGCAGGCGCGCAAATTACCCACTCCTA
GCTCGGGGAGGTAGTGACGAAAAATAACAATACGGGACTCTTACGAGGCCCCGTAATTGGAATGAG
TACACTTTAAATCCTTTAACGAGGATCTATTGGAGGGCAAGTCTGGTGCCAGCAGCCGCGGTAATT
CCAGCTCCAATAGCGTATATTAAAGTTGTTGCAGTTAAAAAGCTCGTAGTTGGATCTCGGGGGCGG
TCCGGCGGTCCTCCTCGCGGAGGTTCACTGCCTGCCGCCCTCCTCACCGGCTTCCCCGGGTGCTCT
TCGCTGAGGCGGCCGGAATGTTTACTTTGAAAAAATTAGAGTGTTCAAAGCAGGCCCTGAATATTG
GCGCATGGAATAATGGAATAGGACCTCGGATTTTGTTGGTTTTCGGAAGTCGAGGTAATGATTAGT
AGGGACGACGGGGGCATTCGTATTGCGCGGTTAGAGGTGAAATTCTTGGATCCGCGCAAGACGAAC
TACAGCGAAGGCGTCTGCCAAGTGCGTCTTCATTAATCAAGAGCGACAGTCAGAGGTTCGAAGACG
ATCAGATACCGTCGTAGTTCTGACCATAAACGATGCCAGCTAGGGATGGGCGGACGTTGCACGACT
CCGCCCGGACCTTCCGGGAAACCAAAGCTTTTGGGTTCCGGGGGAAGTATGGTTGCAAAGCTGAAA
CTTAAAGGAATTGACGGAAGGGCACCACCAGGAGTGGAGCCTGCGGCTTAATTTGACTCAACACGG
GGAAACTCACCCGGCCCGGACACCGTAAGGATTGACAGATTGAAAGCTCTTTCTTGATTCGGTGGG
TGGTGGTGCATGGCCGTTCTTAGTTGGTGGAGCGATTTGTCTGGTTAATTCCGATAACGAACGAGA
CTCTAGCCTGCTAAATAGTTCGCCGGTAAGCCGGCGTAACTTCTTAGAGGGACAAGTGGCGTTCAG
CCACGCGAGATTGAGCAATAACAGGTCTGTGATGCCCTTAGATGTTCGGGGCCGCACGCGCGCTAC
ACTGAAGGACTCAGCTGGT--TCTCCTTGCCTGAGAAGGCTGGGTAACCCGTTGAACCTCCTTCGT
GCTAGGGATTGGGGTTTGCAATTATCCCCCATGAACGAGGAATTCCCAGTAGGCGCGAGTCATCAG
CTCGCGTCGATTACGTCCCTGCCCTTTGTACACACCGCCCGTCGCTACTACCGATCGGATGGTTTA
GTGAGACCTCTGGACTGGTCCCGTCAGCGGGCAACCGCCGACGCGTTGCCGGGAAGAAGGTCGAAC
TTGACTATCTAGAGGAAGTAAAAGTTCACTAAGCGGAGGAAAAGAAACTAACAAGGATTCCCTTAG
TAACGGCGAGTGAAACGGGATGAGCCCAGCACCGAATCCCCTGCCCAC--GGCAGCGGGAACTGTG
GTGTTTAGGAGAGCCTGTGGGGCGGCGG-GGCGTCCGAGTCCTTATGAAAGGGGGTTGCCAGAGCG

GGTGTCAGTCCTCTAGGACGCCCCGTCCCGTCGCTCAGTACCTCCTCGGAGTCGGGTTGTTTGAGA
TTGCAGCCCAAAGCGGGTGGTAAACTCCATCTAAGGCTAAATACTGGCACGAGACCGATAGCAAAC
AAGTACCGTGAGGGAAAGTTGAAAAGAACTTTGAAGAGAGAGTTCAAGAGTACGTGAAACCGTATA
GAAGTAAACGGATGGGACCGCAGTGGACCGGCGGGATTCAGTTCTCGC-GGGCGGATCGGAGACCT
TCACCGGGAATGCACTTTCCGCCGGGACAGCGCCACGACCTGTTCCGGGGCCGCCAGGCCCGGAGG
GAAGGTAGCTCGAGTGTTATAGCCCTTCGGTGTTCCGGCCGGGGGGACATAGGAATACCCGCCGGA
GACCGGTGGACTGTCGAGGACTGCACGCAGTGCTCGGCGACCGCGCCTCTCGGGCCCCCCAGGGTC
AGTGGCGAAGTTGGCTCCCCATCCGACC

>Boccardia_pseudonatrix
CGCCTCCAGATTGGAGGTCCA-TCTGCCCTATGTAAATGGCCGCGGTATCCTGACCGTGCTAAGGT
AGCATAATCATTTGCCGTTTAATTGGCGGCTAGTATGAAAGAATAGACGAGAGCAATGCTGTCTCT
TTTTTACTAAAAATTGATCTTCAGGTACAGTCACCTGAATTCCAAACCGGAACGAAAAGACCCTAT
AGAGCTTTACCTAAACTACTCTAAAGACGGAAGGTTTAGTTGGGGCAACTAGCGAGTACTAAAACC
TCCCTACCCATTTCTCTGATCCCCACAGATTAAAGAAATAGCTACCTTAGGGATAACAGGCTAATT
CCCCTTAAGAGACCATATTGACAGGGGAGTTTGGCACCTCGATGTTGGCTTAGGG-CGCAACTTGA
TGCAGCAGTCCGCGGGTTTGTTCACCCCTTAATACCC-ATATGCTTGTCTCAAAGACTAAGCCATG
CATGTCTAAGTATAAGCTCTACACGGTGAAACCGCGAATGGCTCATTAAATCAGTTATGGTTCCTT
AGATCGTAAAATCCTACTTGGATAACTGTAGTAATTCTAGAGCTAATACATGCACCTCAGCTCCGA
CCCTCGGGAAAGAGCGCTTTTATTAGACCAAAACCAATGGCTCGCGCCAACCTTTGGTGACTCTGG
ATAACTTC-AGCCGACCGCACGGCCGAGCCGGCGGCGTATCTTTCAAATGTCTGCCCTATCAAGTT
TCGATGGTTGGTGACTTGCCAACCATGCTCATAACGGGTAGCGGGGAATCAGGGTTCGATTCCGGA
GAGGGAGCATGAGAAACGGCTACCACATCCAAGGAAGGCAGCAGGCGCGCAAATTACCCACTCCTA
GCTCGGGGAGGTAGTGACGAAAAATAACAATACGGGACTCTTACGAGGCCCCGTAATTGGAATGAG
TACACTTTAAATCCTTTAACGAGGATCTATTGGAGGGCAAGTCTGGTGCCAGCAGCCGCGGTAATT
CCAGCTCCAATAGCGTATATTAAAGTTGTTGCAGTTAAAAAGCTCGTAGTTGGATCTCGGGGGCGG
TCCGGCGGTCCTCCTCGCGGAGGTTCACTGCCTGCCGCCCTCCTCACCGGCTTCCCCGGGTGCTCT
TCGCTGAGGCGGCCGGAATGTTTACTTTGAAAAAATTAGAGTGTTCAAGGCAGGCCCTGAATATTG
GCGCATGGAATAATGGAATAGGACCTCGGATTTTGTTGGTTTTCGGAAGTCGAGGTAATGATTAGT
AGGGACGACGGGGGCATTCGTATTGCGCGGTTAGAGGTGAAATTCTTGGATCCGCGCAAGACGAAC
TACAGCGAAGGCGTCTGCCAAGTGCGTCTTCATTAATCAAGAGCGACAGTCAGAGGTTCGAAGACG
ATCAGATACCGTCGTAGTTCTGACCATAAACGATGCCAGCTAGGGATGGGCGGACGTTGCACGACT
CCGCCCGGACCTTTCGGGAAACCAAAGCTTTTGGGTTCCGGGGGAAGTATGGTTGCAAAGCTGAAA
CTTAAAGGAATTGACGGAAGGGCACCACCAGGAGTGGAGCCTGCGGCTTAATTTGACTCAACACGG
GGAAACTCACCCGGCCCGGACACCGTAAGGATTGACAGATTGAAAGCTCTTTCTTGATTCGGTGGG
TGGTGGTGCATGGCCGTTCTTAGTTGGTGGAGCGATTTGTCTGGTTAATTCCGATAACGAACGAGA

CTCTAGCCTGCTAAATAGTTCGCCGGTAAGCCGGCGCAACTTCTTAGAGGGACAAGTGGCTTTCAG
CCACGCGAGATTGAGCAATAACAGGTCTGTGATGCCCTTAGATGTTCGGGGCCGCACGCGCGCTAC
ACTGAAGGACTCAGCTGGT--TCTCCTGGCCTGAAAAGGTCGGGCAACCCGTTGAACCTCCTTCGT
GCTAGGGATTGGGGGTTGCAATTTTTCCCCATGAACGAGGAATTCCCAGTAGGCGCGAGTCATCAG
CTCGCGTCGATTACGTCCCTGCCCTTTGTACACACCGCCCGTCGCTACTACCGATCGGATGGTTTA
GTGAGACCCTCGGACTGGTCCCGTCAGTGGGCAACTGCTGACGCGTTGCCGGGAAGAAGGTCGAAC
TTGACTATCTAGAGGAAGTAAAAGT-CACTAAGCGGAGGAAAAGAAACTAACCAGGATTCCCTGAG
TAACGGCGAGTGAAACGGGAAGAGCCCAGCACCGAATCCCCTGGCTTTTAGCTAGCGGGAACTGTG
GTGTTTAGGAGAGCCTGTGGAGCGGCGG-AGCGTCCGAGTCCCTATGAACGGGGGCAGCCAGAGCG
GGTGTCAGTCCTCTAGGACGCCCCGTCCCGTTGCTCAGTACCTCCTCGGAGTCGGGTTGTTTGAGA
TTGCAGCCCAAAGTGGGTGGTAAACTCCATYTAAGGCTAAATACTGGCACGAGACCGATAGCAAAC
AAGTACCGTGAGGGAAAGTTGAAAAGAACTTTGAAGAGAGAGTTCAAGAGTACGTGAAACCGTATA
GAAGTAAACGGATGGGACCGCAGTGTACCGGCGGGATTCAGTTCGCTC-GGGCGGCTCGGAGACCT
TTACCGGGAATGCACTTTCCGTCGGGACAGCGCCACGACCGGTTCTCGAGCCGCCAGGCCCGGAGG
TAAGGTAGCTCGAGTATTATAGACCTCCGGTGTTCCGGCCGGGGGGACCGAGGAATACCCGCCGGA
GACCGGTGGACTGGTGAGGACTGCTCGCAGTGCTCGCCGACCGCGCCGTTCGGGCCCCCCAGGGTC
TGTGGCGAAGTTGGC-CCCCATCCAACC

>Dipolydora_giardi
----------TTAAGAGGTCATTCTGCCCTATGTAAATGGCCGCGGTAGCCTGACCGTGCTAAGGT
AGCATAATCATTTGCCGTTTAATTGGTGGCTGGAATGAATGAATATACGAGAGCTCTACTGTCTCA
CCTTTAATAAAAATTGACCCTCAAGTGAAGAGACTTGAATTTCCCAAAAAGACGAAAAGACCCTAT
AGAGCTTTACCTAAAACATTCTACAAGTCTAAGGTTTAGTTGGGGCAACTAGAGAGAACTAAAACC
TCTCTTCCTAAAAAATTGATCCGTTACGACATCAGAAAAAGCTACCTTAGGGATAACAGGCTAATC
CTCTTTAAGAGACCATATTGACAAGAGGGTTTGGCACCTCGATGTTGGCTCAAGGTTACAGTCTGA
TGCAGCAGTAAGTAGGTTTGTTCACCCTTTAAATCCTTATATGCTTGTCTCAAAGACTAAGCCATG
CATGTCTAAGTATAAGCTCTACATGGTGAAACCGCGAATGGCTCATTAAATCAGTTATGGTTCCTT
AGATCTTAAAATCCTACTTGGATAACTGTAGTAATTCTAGAGCTAATACATGCAATCAAGCTCCGA
CCCGAGGGAAAGAGCGCTTTTATTAGACCAAAACCAATGGTTCGCGCCAACCTTTGGTGACTCTGG
ATAACTTC-AGCTGATCGCAAGGCCGAGCCGGCGACGTATCTTTCAAATGTCTGCCCTATCAAGTT
TCGATGGTTGGTGACTTGCCAACCATGCTCGTAACGGGTAGCGGGGAATCAGGGTTCGATTCCGGA
GAGGGAGCATGAGAAACGGCTACCACATCCAAGGAAGGCAGCAGGCGCGCAAATTACCCACTCCTG
GATCGGGGAGGTAGTGACGAAAAATAACAATACGGGACTCTTACGAGGCCCCGTAATTGGAATGAG
TACACTTTAAATCCTTTAACGAGGATCTATTGGAGGGCAAGTCTGGTGCCAGCAGCCGCGGTAATT
CCAGCTCCAATAGCGTATATTAAAGTTGTTGCAGTTAAAAAGCTCGTAGTTGGATCTCGGGAGCGG
TCCGGCGGTCCTCCGCAAGGAGGTTCACTGCCCGCCGCTCTCCTCACCGGCTTGCCCGGGTGCTCT

TCGTTGGGTTGGCCGGAATGTTTACTTTGAAAAAATTAGAGTGTTCAAAGCAGGCCCTGAATATTG

GCGCATGGAATAATGGAATAGGACCTCGGATTTTGTTGGTTTTCGGAAGTCGAGGTAATGATTAGT

AGGGACGACGGGGGCATTCGTATTGCACGGTTAGAGGTGAAATTCTTGGATCCGTGCAAGACGAAC

TACAGCGAAGGCGTCTGCCAAGTGCGTCTTCATTAATCAAGAGCGACAGTCAGAGGTTCGAAGACG

ATCAGATACCGTCGTAGTTCTGACCATAAACGATGCCAGCTAGGGATGGGCGGACGTTGTTCGACT

CCGCCCGGACCTTCCGGGAAACCAAAGCTTTTGGGTTCCGGGGGAAGTATGGTTGCAAAGCTGAAA

CTTAAAGGAATTGACGGAAGGGCACCACCAGGAGTGGAGCCTGCGGCTTAATTTGACTCAACACGG

GGAAACTCACCCGGCCCGGACACCGTAAGGATTGACAGATTGAGAGCTCTTTCTTGATTCGGTGGG

TGGTGGTGCATGGCCGTTCTTAGTTGGTGGAGCGATTTGTCTGGTTAATTCCGATAACGAACGAGA

CTCTAGCCTGCTAAATAGTTCGTCGGTAAGCCGGCGCAACTTCTTAGAGGGACAAGTGGCGTTTAG

CCACGCGAGATTGAGCAATAACAGGTCTGTGATGCCCTTAGATGTTCGGGGCCGCACGCGCGCTAC

ACTGAAGGAATCAGCTGGT--TCTCCTGGCCTGAAAAGGTCGGGCAACCCGTTGAACCTCCTTCGT

GCTAGGGATTGGGGGTTGCAATTCTTCCCCATGAACGAGGAATTCCCAGTAGGCGCGAGTCATCAG

CTCGCGTCGATTACGTCCCTGCCCTTTGTACACACCGCCCGTCGCTACTACCGATCGGATGGTTTA

GTGAGACCCTCGGACTGGTCCCGTCAGCGGGCAACCGCCGACGCGTTGCCGGGAAGAAGGTCGAAC

TTGACTATCTAGAGGAAGTAAAAGT-CACTAAGCGGAGGAAAAGAAACTAACCAGGATTCCCTTAG

TAACGGCGAGTGAAACGGGAAGTGCCCAGCACCGAATCCCCTGCCGTTT-GGCAGCGGGAAATGTG

GTGTTTAGGAGAGCCTGTGGAGCGGCGG-AGCGTCCGAGTCCTTATGAAAGGGGGCAGCCAGAGCG

GGTGTTAGTCCTCTAGGACGTCCCGTCCCGTTGCTCAGTACCTCCTCGGAGTCGGGTTGTTTGTGA

ATGCAGCCCTAAGTGGGTGGTAAACTCCATCTAAGGCTAAATACTGGCACGAGACCGATAGCAAAC

AAGTACCGTGAGGGAAAGTTGAAAAGAACTTTGAAGAGAGAGTTCAAGAGTACGTGAAACCGTATA

GAAGTAAACGGATGGGACCGCAGTGGACCGACGGGATTCAATTCTTTCGGGGCGGATCAGAGACCT

TAACCGGGAATGCACTTTCCGTCGGGACAGCGCCAGGACCGGTTCTCCGGCCGCCAGGCCCGTTGG

GAAGGTAGCTCGAGTGTTATAGCCCTACGGTGTTCCGGCCGGGAGGACCGAGGAATACCCGCCGGA

GACCGTTGGACTGGCGAGGACTGCTCGCAGTGCTCGTCGACCGCGGCGTTCAGGCCCCCCAGGGTC

GCTGGCGAAGTTGGCTCCCCATCCGAC-
